# Synthesis and Characterization of Methoxylated Oligosilyl Group 4 Metallocenes

**DOI:** 10.1021/acs.inorgchem.2c02112

**Published:** 2022-09-01

**Authors:** Aileen Sauermoser, Thomas Lainer, Gabriel Glotz, Frank Czerny, Bettina Schweda, Roland C. Fischer, Michael Haas

**Affiliations:** † Institute of Inorganic Chemistry, Graz University of Technology, Stremayrgasse 9/V, A-8010 Graz, Austria; ‡ Institute of Physical and Theoretical Chemistry, Graz University of Technology, Stremayrgasse 9/II, 8010 Graz, Austria; § Department of Chemistry: Metalorganics and Inorganic Materials, Technische Universität Berlin, 10623 Berlin, Germany; ∥ Institute for Chemistry and Technology of Materials, Graz University of Technology, Stremayrgasse 9/IV, A-8010 Graz, Austria

## Abstract

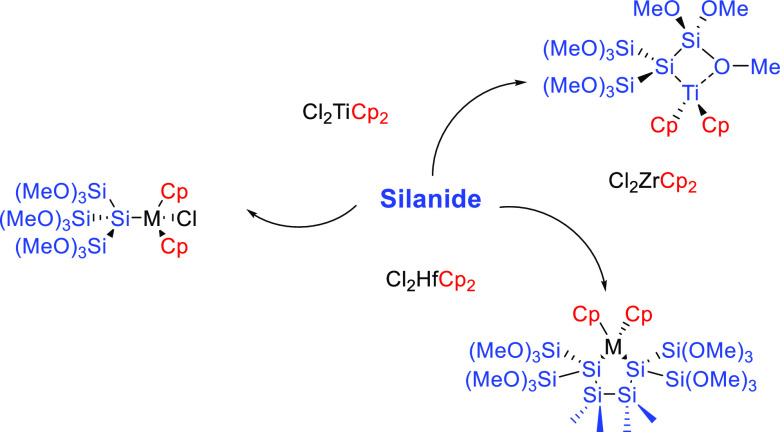

New methoxylated oligosilyl-substituted metallocenes were synthesized by the reaction of two oligosilanides with different metallocene dichlorides (M = Ti, Zr, and Hf). The first investigated tris(trimethoxysilyl)silanide [(MeO)_3_Si]_3_SiK (**1**) underwent a selective monosubstitution to the respective oligosilyl-decorated metallocenes [(MeO)_3_Si]_3_SiMClCp_2_ (**2**–**4**). Surprisingly, the attempted disilylation with this silanide was not possible. However, in the case of titanocene dichloride, a stable radical [(MeO)_3_Si]_3_SiTiCp_2_ (**5**) was formed. The unsuccessful isolation of bisilylated metallocenes encouraged us to investigate the reactivity of another silanide. Therefore, we synthesized a hitherto unknown disilanide K[(MeO)_3_Si]_2_Si(SiMe_2_)_2_Si[(MeO)_3_Si]_2_K (**8**), which was accessible in good yields. The reaction of compound **8** and different metallocene dichlorides (M = Ti, Zr, and Hf) gave rise to the formation of heterocyclic compounds **9**–**11** in good yields.

## Abstract 2

New methoxylated oligosilyl-substituted metallocenes were synthesized by the reaction of two silanides with metallocene dichlorides (M = Ti, Zr, and Hf).

## Introduction

**Chart 1:**
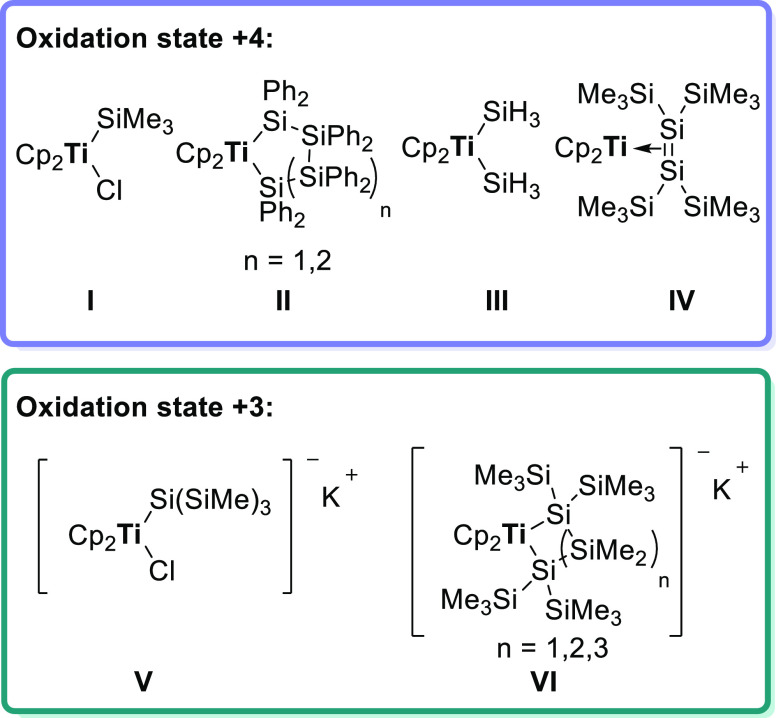
Reported Silyl-Substituted Titanocenes

The synthesis of silyl-substituted group 4 metallocenes continues to be a comprehensively researched field in contemporary metalorganic chemistry. The reasons for these studies are the multiple applications of these compounds as catalysts or precatalysts for a number of chemical transformations. Among these, the dehydrogenative polymerization of hydrosilanes stands out.^1−4^ A pioneer in this research field is the group of Harrod, who explored the catalytic reactivity of Cp_2_TiMe_2_ in the presence of PhSiH_3_.^5−7^^5−7^ Subsequently, Tilley and co-workers showed that zirconocenes and hafnocenes (**VII**) also have the ability to polymerize phenylsilanes.^1,8−10^ Here, Tilley proposed a σ-bond metathesis mechanism for the formation of polymers.^1,9^ Although these metallocenes were heavily investigated as catalysts, so far only a few silyl-substituted titanocenes in the oxidation state +4 have been reported. Rösch et al. synthesized Cp_2_Ti(Cl)SiMe_3_ (**I**) by the reaction of Cp_2_TiCl_2_ with Al(SiMe_3_)_3_·Et_2_O.^11^ Additionally, Cp_2_Ti(SiPh_2_)*_n_* (*n* = 4 and 5 (**II**)) was successfully reported by Holtman et al. and Igonin et al. by the reaction of Cp_2_TiCl_2_ with Li(Ph_2_Si)*_n_*Li.^12−15^ Cp_2_Ti(SiH_3_)_2_ (**III**) was achieved by Harrod and co-workers by reacting Cp_2_TiCl_2_ with H_3_SiK.^16^ Also, Marschner and co-workers successfully synthesized a titanocene disilene complex (**IV**).^17^ Furthermore, the same group reported on the synthesis of different titanocenes (**V** and **VI**) in the oxidation state +3 (Chart 1).^18,19^ In contrast to this, a wide range of oligosilyl-substituted zircono- and hafnocenes are known. Tilley, as well as Marschner and co-workers, have extensively studied these compounds.^8,10,20−33^

**Chart 2:**
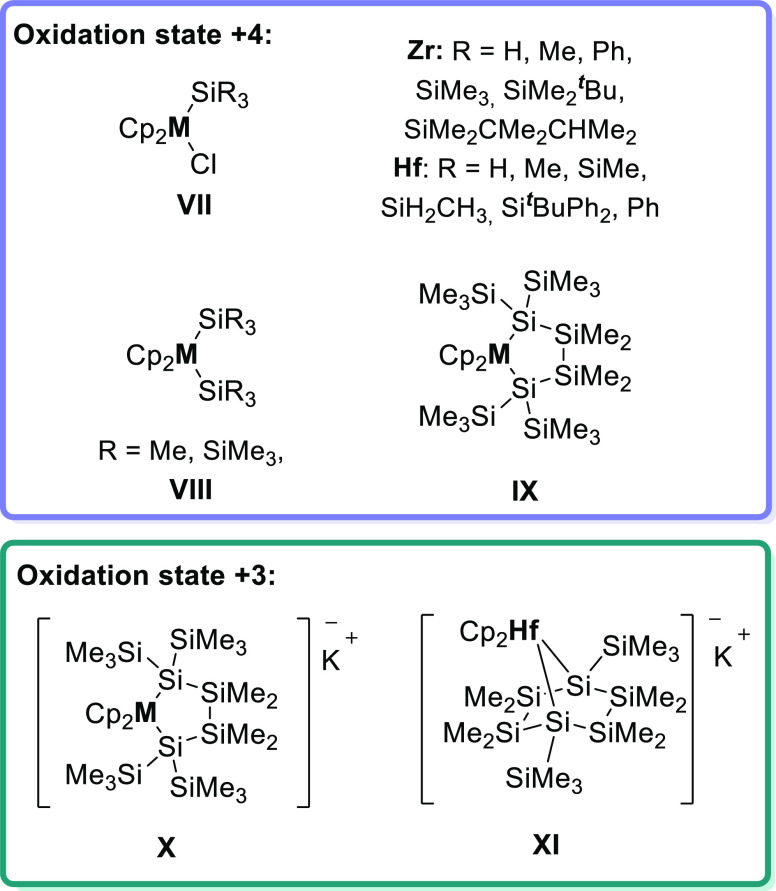
Reported Silyl-Substituted Zirconocenes and Hafnocenes (M = Zr and Hf)

Some selected examples of different zirconocenes and hafnocenes are shown in Chart 2. Tilley et al. synthesized mono- and disubstituted hafno- and zirconocenes with different silyl substituents (**VII** and **VIII**).^10,22,25−27^ Moreover, Marschner and co-workers successfully published a series of hafno- and zirconocenes with different silyl backbones (**VII**, **VIII**, and **IX**).^19,28,30,32^ Additionally, they also synthesized two different hafnocenes and one zirconocene in the oxidation state +3 (**X** and **XI**).^19,31^

**Scheme 1:**
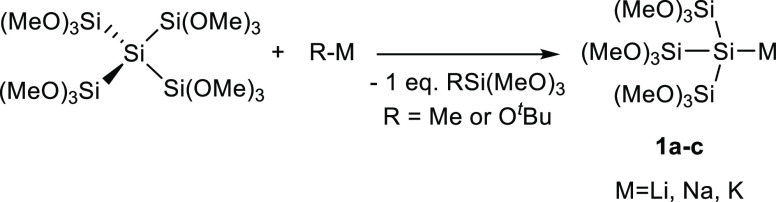
Reaction of Dodecamethoxyneopentasilane with Suitable Bases Forming **1a**–**c**

On the basis of the limited availability of different substituted silyl anions, many groups focused on the use of simple silyl- or tris(trimethylsilyl)silyl ligands, which gave rise to only a moderate variability of the isolated molecules. Recently, we introduced the straightforward synthesis of tris(trimethoxysilyl)silanides (M = Li, Na, K) **1a**–**c** by reacting dodecamethoxyneopentasilane with equimolar amounts of a suitable base (see Scheme 1).

The reactivity of **1a**–**c** toward different carbon and silicon electrophiles has already been successfully demonstrated. Moreover, we were able to show that these permethoxy-substituted silanides react more selectively than other known silanides. Therefore, we want to implement this new silyl substituent as a ligand for group 4 metallocenes. Due to the fact that no significant correlation between the used silanide and the yield of the expected product was observed, only **1c** was used as nucleophile.^34^

## Results and Discussion

### Synthesis and Characterization

**Scheme 2:**
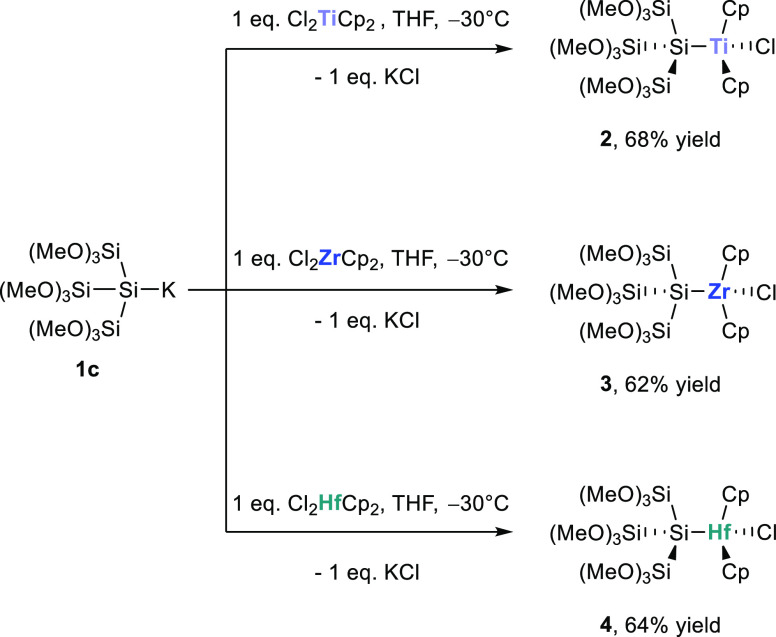
Reaction of **1c** with Cl_2_MCp_2_ (M = Ti, Zr, Hf) to Synthesize the Oligosilyl-Substituted Metallocenes **2**–**4**

The entry into this chemistry is provided by the selective reaction of **1c** with equimolar amounts of Cl_2_MCp_2_ (M = Ti, Zr, Hf). The desired product formation to oligosilyl-substituted metallocenes **2**–**4** with all central atoms in an oxidation state of +4 was observable in good to excellent yields (Scheme 2). Especially, the formation of **2** was surprising to us, as this is in stark contrast to all previously reported reactions of silanides with titanocene dichlorides. Marschner and co-workers reported that oligosilyl anions with alkyl or aryl groups do not react to the silylated titanocene; instead, an ate-complex was formed.^18,19^ Recently, Scheschkewitz and co-workers reported on zirconocene- and hafnocene-substituted siliconoid derivatives, while titanocene dichloride as an electrophile gave rise to a complex product mixture.^35^ Therefore, compound **2** can be seen as a new interesting compound for further investigations including its catalytic activity.

NMR data are consistent with the proposed structures, exhibiting one resonance line in the ^29^Si NMR spectrum for the three trimethoxysilyl groups and one signal for the quaternary silicon atom (compare Table 1). The quaternary silicon signal for compound **2** shows a significant low-field shift when compared to **3** and **4**, whereas the signals for the −*Si*(OMe)_3_ groups adopt only a slight change in the shifts. By comparison of the ^29^Si NMR signals of compounds **1c**(34) and **2**–**4**, we found a significant high-field shift for central silicon signals, indicating the deshielding of the silicon atoms based on the formation of a Si–M (M = Ti, Zr, Hf) bond. All other analytical and spectroscopic data that support the structural assignments are given in the Experimental Section together with experimental details.

**Table 1:** Comparison of ^29^Si NMR of **1c** with **2**–**4**

	**1c**	**2**	**3**	**4**
–*Si*(OMe)_3_	–4.6	–33.1	–29.3	–27.2
–*Si*(Si(OMe)_3_)_3_	–269.1	–88.1	–128.0	–117.4

**Figure 1:**
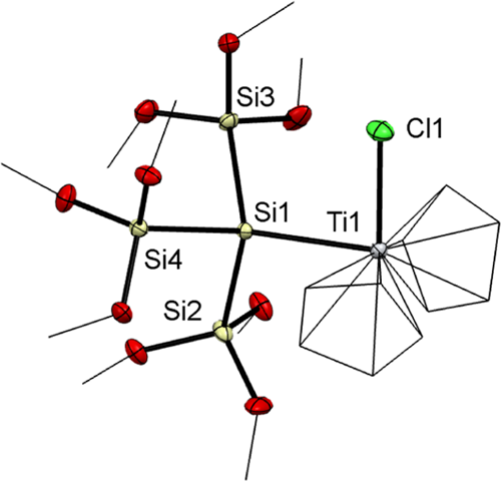
Oak Ridge thermal-ellipsoid plot (ORTEP) for compound **2**. Thermal ellipsoids are depicted at the 50% probability level. Hydrogen atoms are omitted and carbon atoms are wireframed for clarity. Selected bond lengths (Å) and bond angles (deg) with estimated standard deviations: Ti(1)–Cl(1) 2.3388(7), Ti(1)–Si(1) 2.7037(7), Si(1)–Si(2) 2.3449(9), Si(1)–Si(3) 2.3589(8), Si(1)–Si(4) 2.3485(9), Cl(1)–Ti(1)–Si(1) 94.40(3), Si(2)Si(1)–Si(4) 104.50(3), Si(2)–Si(1)–Si(3) 105.61(3), Si(4)–Si(1)–Si(3) 101.98(3), Si(2)–Si(1)–Ti(1) 109.43(3), Si(4)–Si(1)–Ti(1) 116.42(3), Si(3)–Si(1)–Ti(1) 117.61(3).

For **2**–**4**, single crystals suitable for X-ray structure analysis could be grown in *n*-pentane at −30 °C. The structure of **2** is depicted in Figure 1. The X-ray structures of **3** and **4** can be found in the Supporting Information in Figures S30 and S31. Compounds **2**–**4** crystallized in the monoclinic space group *P*2(1)*c*. Table 2 summarizes selected average bond lengths alongside selected angles.

**Table 2:** Selected Average Bond Lengths (Å) and Bond Angles (deg) of **2**–**4**^a^

	M–Cl	M–Si	Si–Si	Cl–M–Si	Si–Si–M	Si–Si–Si
**2**	2.3388(7)	2.7037(7)	2.3508(7)	94.40(3)	116.42(3)	104.50(3)
**3**	2.4310(7)	2.8118(7)	2.3413(3)	98.91(2)	114.54(3)	106.33(4)
**4**	2.4049(14)	2.7770(16)	2.3387(4)	97.94(5)	115.40(7)	105.77(8)

a M = Ti, Zr, Hf.

**Scheme 3:**
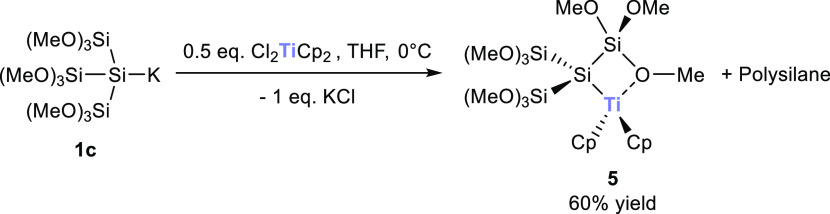
Reaction of **1c** with 0.5 equiv of Cl_2_TiCp_2_

The observed bond lengths of the metal–chlorine (M–Cl) bond for **2**–**4** are in the same region as the known distances of Cl_2_MCp_2_ (Ti–Cl 2.364(3) Å; Zr–Cl 2.441(2) Å; Hf–Cl 2.423(3) Å).^36^ Compound **2** adopts a Ti(1)–Si(1) bond length of 2.3388(7) Å, which is in the range of other reported Ti–Si bond lengths (2.159(13)–2.8347(18) Å).^11,18,37^ Compound **3** shows a Zr(1)–Si(1) bond length of 2.8118(7) Å, which is consistent with the distance found in the literature (2.8503(11)–2.8950(10) Å).^19^ However, the hafnium derivative **4** adopts a bond length of 2.7770(16) Å for Hf(1)–Si(1) that is significantly decreased in comparison to the known values of 2.849(2)–2.863(2) Å.^19^ The bond lengths of all Si–Si bonds show no deviations from published values (2.385 Å).^38^ The straightforward synthesis of compounds **2**–**4** encouraged us to systematically investigate the reactions of our silanides with metallocenes. The next logical synthetic target was the bis-silylation of the respective metallocenes. Therefore, we reacted **1c** with 0.5 equiv of Cl_2_MCp_2_ (M = Ti, Zr, and Hf). However, in the case of zirconocene dichloride and hafnocene dichloride, no characterizable products were observed via NMR and electron paramagnetic resonance (EPR) spectroscopies, whereas the reaction of **1c** with titanocene dichloride gave rise to a selective radical formation, which was determined by EPR spectroscopy and X-ray crystallography (Scheme 3). Moreover, this radical (compound **5**) is persistent at room temperature (RT) and does not show any degradation products over time.

**Figure 2:**
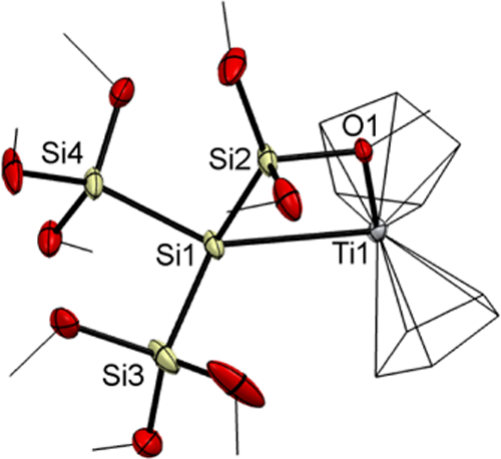
ORTEP for compound **5**. Thermal ellipsoids are depicted at the 50% probability level. Hydrogen atoms are omitted and carbon atoms are wireframed for clarity. Selected bond lengths (Å) and bond angles (deg) with estimated standard deviations: Ti(1)–O(1) 2.2150(17), Ti(1)–Si(1) 2.7432(7), Si(1)–Si(2) 2.3133(10), Si(1)–Si(3) 2.3322(10), Si(1)–Si(4) 2.3319(11), O(1)–Ti(1)–Si(1) 73.61(4), Si(2)–Si(1)–Si(4) 109.21 (4), Si(2)–Si(1)–Si(3) 100.95, Si(4)–Si(1)–Si(3) 104.42(4), Si(2)–Si(1)–Ti(1) 78.75(3), Si(4)–Si(1)–Ti(1) 129.92(4), Si(3)–Si(1)–Ti(1) 122.97(4).

Compound **5** was isolable in good yield and single crystals suitable for X-ray structure analysis could be grown in *n*-pentane at −30 °C (see Figure 2). Compound **5** crystallized in the triclinic space group *P*1̅ with a unit cell containing two molecules (see the Supporting Information).

**Figure 3:**
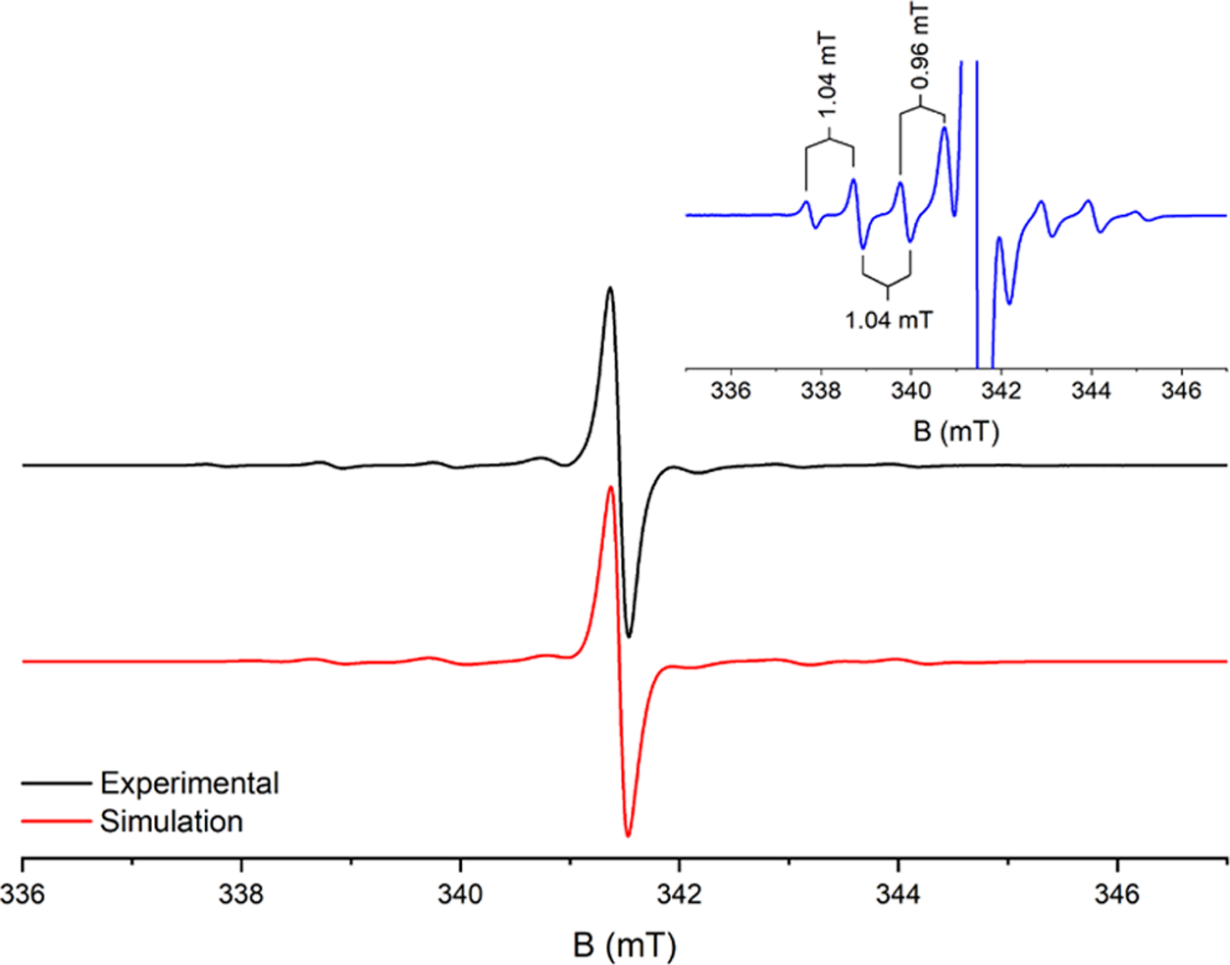
Experimental (black line) and simulated (red line) X-band EPR spectra of **5** at 280 K in C_6_D_6_, and the inset presents the experimental spectrum with increased instrument gain to better visualize small intensity signals.

As depicted in Figure 2, one oxygen of a methoxy group donates a lone pair and consequently stabilizes the titanium radical. This donation significantly influences the orientation of one trimethoxysilyl group. Accordingly, the Si(2)–Si(1)–Ti(1) angle with 78.75(3)° of compound **5** is highly decreased in comparison to the respective angle of 109.43(3)° in compound **2**. Additionally, the Si(1)–Si(2) bond length is slightly decreased in comparison to compound **2**. Moreover, the Ti(1)–Si(1) bond length is significantly increased compared to compound **2**. Interestingly, also the Ti(1)–O(1) bond length is increased in comparison to literature values (1.914–2.099 Å).^39^ The d^1^ configuration of **5** allows EPR spectroscopy to characterize this compound. The metalate **5** exhibited an isotropic EPR signal presented as one sharp singlet with satellites due to the ^47^Ti with *I* = 5/2 and ^49^Ti with *I* = 7/2 in their respective natural abundances (Figure 3). The *g*-factor of 1.9809 was determined using DPPH in C_6_D_6_ as an external reference, which is similar to the values found in the literature for Ti(III) complexes.^40^ Analytical and spectroscopic data that well support the structural assignment are given in the Experimental Section.

**Scheme 4:**
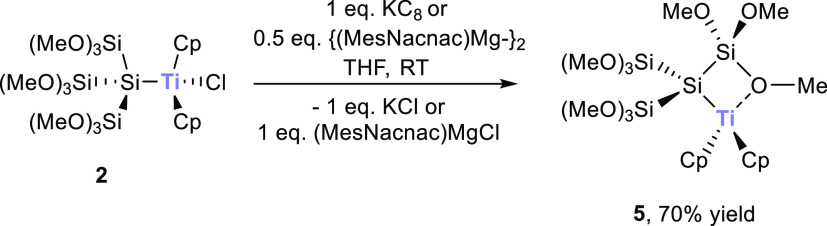
Reduction of **2** with KC_8_ or [{(MesNacnac)Mg}_2_] (MesNacnac = [(MesNCMe)_2_CH]^−^, Mes = Mesityl)

The formation of **5** intrigued us to further investigate the reactivity of **2**. Consequently, we reacted **2** with the moderate reducing reagent [{(MesNacnac)Mg}_2_] as well as KC_8_. As expected, the same product was observed, and the yields were increased by 10% (Scheme 4). Following this result, we also tested the reduction of **3** and **4** with the same reaction conditions, but again, no characterizable products were formed.

**Scheme 5:**
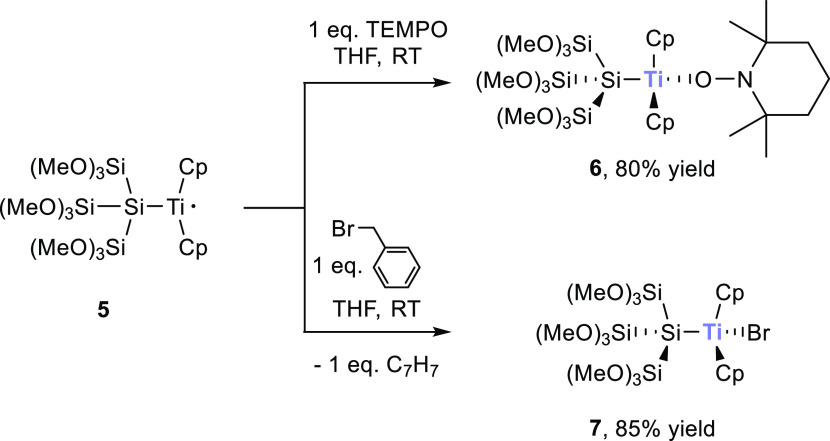
Reaction of **5** with TEMPO or (Bromomethyl)benzene to Form Compounds **6** and **7**

To determine the reactivity of the radical, **5** was reacted with two standard radical quenching reagents. Therefore, we reacted **5** with equimolar amounts of (2,2,6,6-tetramethylpiperidin-1-yl)oxyl (TEMPO) or equimolar amounts of (bromomethyl)benzene and found the selective formation of the corresponding trapping products **6** and **7** in good yields (see Scheme 5). NMR data are consistent with the proposed structures and are depicted in the Experimental Section.

**Scheme 6:**
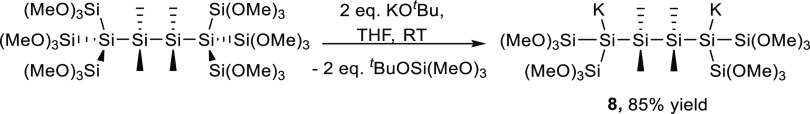
Reaction of 1,1,1,6,6,6-Hexamethoxy-3,3,4,4-tetramethyl-2,2,5,5-tetrakis-(trimethoxysilyl)hexasilane with 2 equiv of KO^*t*^Bu to the Respective Dianion **8**(34)

The unsuccessful isolation of the bis-silylated metallocenes with the usage of 2 equiv of **1c** encouraged us to investigate the reactivity of another silanide. Therefore, 1,1,1,6,6,6-hexamethoxy-3,3,4,4-tetramethyl-2,2,5,5-tetrakis(trimethoxysilyl)-hexasilane was synthesized according to published procedures^34^ and used for the synthesis of the dianion **8** shown in Scheme 6.

**Figure 4:**
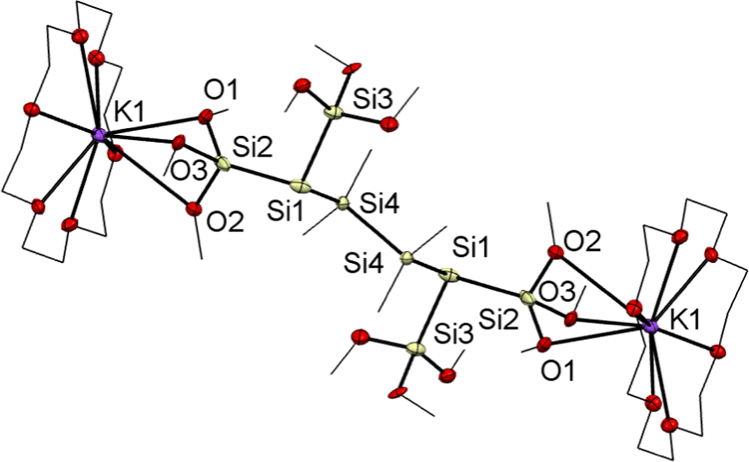
ORTEP for compound **8** stabilized by 18-crown-6. Thermal ellipsoids are depicted at the 50% probability level. Hydrogen atoms are omitted and carbon atoms are wireframed for clarity. Selected bond lengths (Å) and bond angles (deg) with estimated standard deviations: K(1)–O(1) 2.941(2), K(1)–O(2) 3.289(2), K(1)–O(3) 3.040(11), O(1)–Si(2) 1.660(3), Si(2)–O(2) 1.670(2), Si(2)–O(3) 1.662(5), Si(1)–Si(2) 2.3023(14), Si(1)–Si(3) 2.3100(12), Si(1)–Si(4) 2.3568(12), O(1)–K(1)–Si(2) 26.87(5), O(2)–K(1)–Si(2) 27.47(4), O(3)–K(1)–Si(2) 27.20(8), O(1)–K(1)–O(2) 47.74(5), O(1)–K(1)–O(3) 43.54(10), O(3)–K(1)–O(2) 48.26(10), Si(2)–Si(1)–Si(3) 102.24(5), Si(2)–Si(1)–Si(4) 105.83(5), Si(3)–Si(1)–Si(4) 103.32(4).

For **8**, single crystals suitable for X-ray structure analysis could be grown in tetrahydrofuran (THF) at −30 °C after the addition of 2 equiv of 18-crown-6. The structure is depicted in Figure 4. Compound **8** crystallized in the triclinic space group *P*1̅ with a unit cell containing one molecule.

All Si–O bond lengths of compound **8** (1.660(3), 1.670(2), 1.662(5)) are slightly increased when compared to reported values (1.576–1.632 Å).^41,42^ All Si–Si bonds are comparable to known distances.^11,37^ The potassium atoms are coordinated by the crown ether ligands. Additionally, the oxygen atoms of three methoxy groups on each site are attached to potassium in a chelating coordination mode. The interesting structural features are the different K–O bond lengths with 2.941(2) Å for K(1)–O(1), 3.040(11) Å for K(1)–O(2), and 3.289(2) Å for K(1)–O(3). Here, only the K(1)–O(1) distance has a bond length of 2.941(2) Å, which is consistent with the already reported distances.^43^

**Scheme 7:**
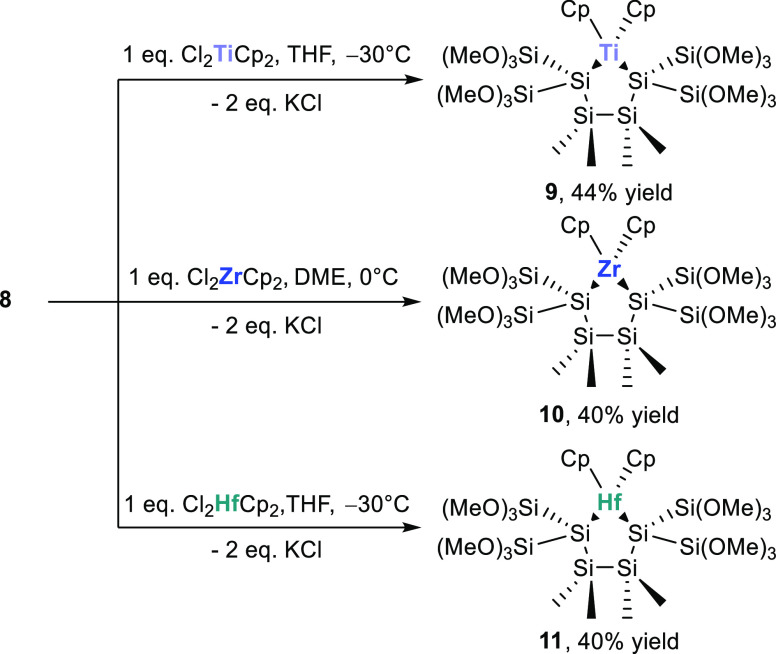
Reaction of **8** with Cl_2_MCp_2_ (M = Ti, Zr, Hf) to the Heterocyclic Compounds **9**–**11**

Subsequently, we systematically investigated the reactions of our new silanides with metallocenes to obtain heterocyclic compounds. Therefore, we reacted **8** with equimolar amounts of Cl_2_MCp_2_ (M = Ti, Zr, Hf) and found the selective formation of compounds **9**–**11** in moderate yields as shown in Scheme 7. Analytical and spectroscopic data that well support the structural assignment are given in the Experimental Section.

Table 3 compares the ^29^Si NMR spectra of the dianion **8** and the heterocycles **9**–**11**. Again, the ^29^Si NMR signals of compounds **8** and **9**–**11** showed a significant high-field shift for central silicon signals, indicating the deshielding of the silicon atoms based on the formation of Si–M (M = Ti, Zr, Hf) bonds. The quaternary silicon signal for compound **10** shows a significant high-field shift as compared to **9** and **11**. Even between **9** and **11**, a significant shift was observed. Comparing compounds **9**–**11** to **2**–**4**, a similar trend of the ^29^Si NMR shifts could be detected. Further experimental details are depicted in the Experimental Section.

**Table 3:** ^29^Si NMR Data for **8**–**11**

	**8**	**9**	**10**		**11**
–*Si*(OMe)_3_	0.5	–30.7	–26.6		–23.8
–*Si*(Si(OMe)_3_)_2_	–243.4	–57.6	–94.6	–75.7	
–*Si*(CH_3_)_2_	–26.1	–7.8	–15.4		–16.4

**Figure 5:**
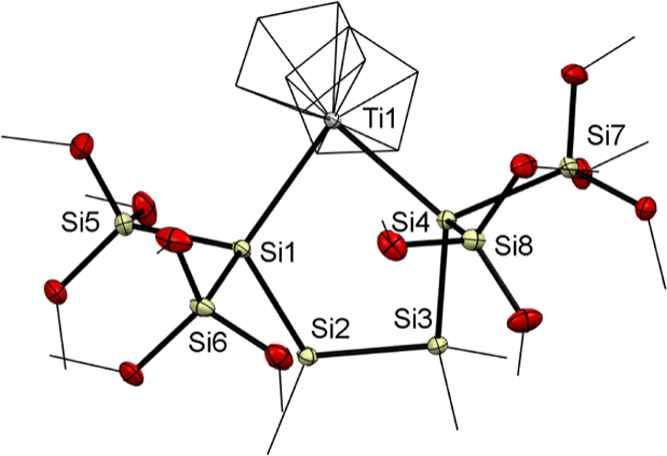
ORTEP for compound **9**. Thermal ellipsoids are depicted at the 50% probability level. Hydrogen atoms are omitted and carbon atoms are wireframed for clarity. Selected bond lengths (Å) and bond angles (deg) with estimated standard deviations: Ti(1)–Si(1) 2.6697(9), Ti(1)–Si(4) 2.6884(9), Si(1)–Si(2) 2.3624(11), Si(1)–Si(5) 2.3261(11), Si(1)–Si(6) 2.3355(11), Si(2)–Si(3) 2.3531(11), Si(3)–Si(4) 2.3755(10), Si(4)–Si(8) 2.3399(11), Si(4)–Si(7) 2.3282(11), Si(1)–Ti(1)–Si(4) 84.63(3), Si(2)–Si(1)–Ti(1) 111.29(3), Si(5)–Si(1)–Ti(1) 110.85(4), Si(6)–Si(1)–Ti(1) 120.12(4), Si(3)-Si(4)–Ti(1) 112.80(4), Si(8)–Si(4)–Ti(1) 120.90(4), Si(7)–Si(4)–Ti(1) 109.49(4), Si(5), Si(1), Si(2) 109.84(4), Si(5)–Si(1)–Si(6) 103.98(4), Si(6)–Si(1)–Si(2) 99.95(4), Si(3)–Si(2)–Si(1) 103.92(4), Si(2)–Si(3)–Si(4) 106.11(4), Si(8)–Si(4)–Si(3) 108.11(4), Si(7)–Si(4)–Si(3) 107.40(4), Si(7)–Si(4)–Si(8) 96.31(4).

Crystals of **9** of sufficient quality for single-crystal X-ray crystallography were obtained by crystallization at −30 °C from an *n*-pentane solution. The molecular structure is depicted in Figure 5 along with selected bond distances and dihedral angles. Compound **9** crystallized in the triclinic space group *P*1̅. The unit cell contains six molecules. The average Ti–Si bond length (2.6791(2) Å) of **9** is significantly shorter compared to that of compound **2** (2.7037(7) Å) and moreover in the lower end of similar molecules.^18^ All Si–Si bonds are comparable to known distances.^11,37^ In addition, the five-membered ring adopts envelope conformation. The titanium atom (Ti1) lies 1.215(2) Å outside the plane formed by the silicon atoms.

**Figure 6:**
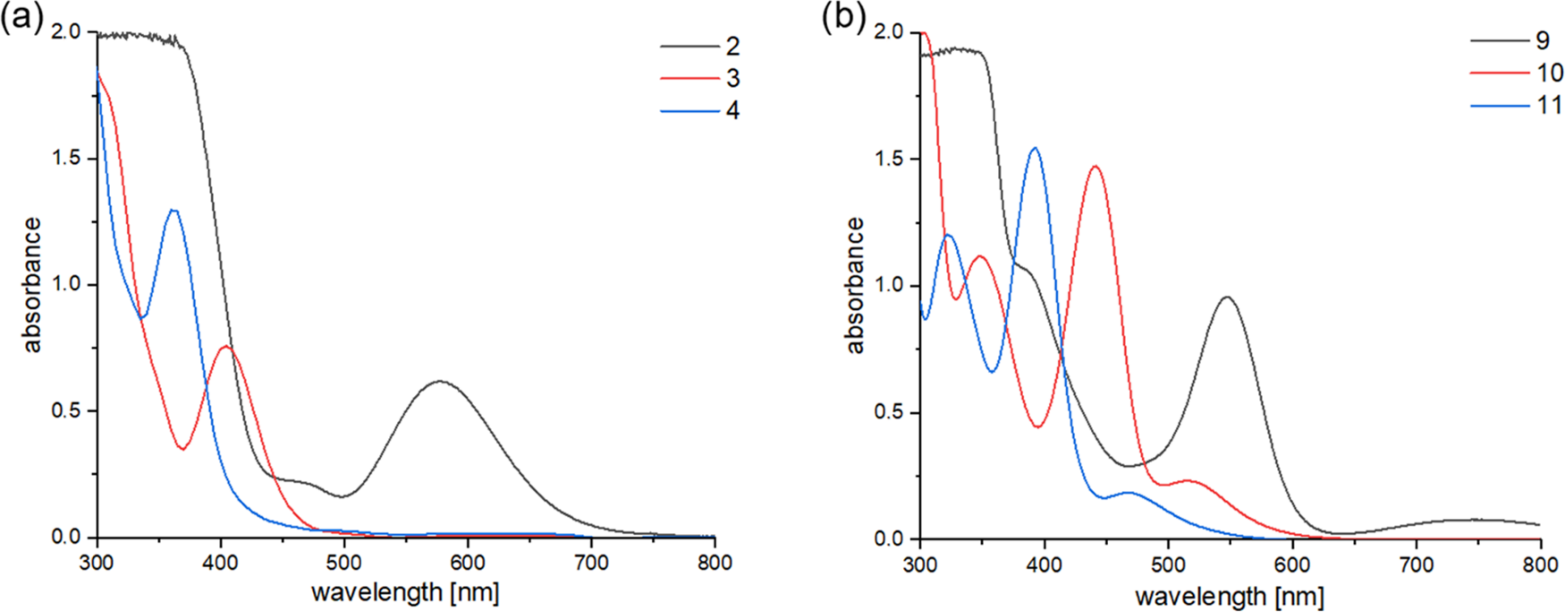
UV–vis spectra of compounds **2**–**4** (a) and **9**–**11** (b) (*c* = 1 × 10^–3^ mol/L; solvent: *n*-hexane).

**Figure 7:**
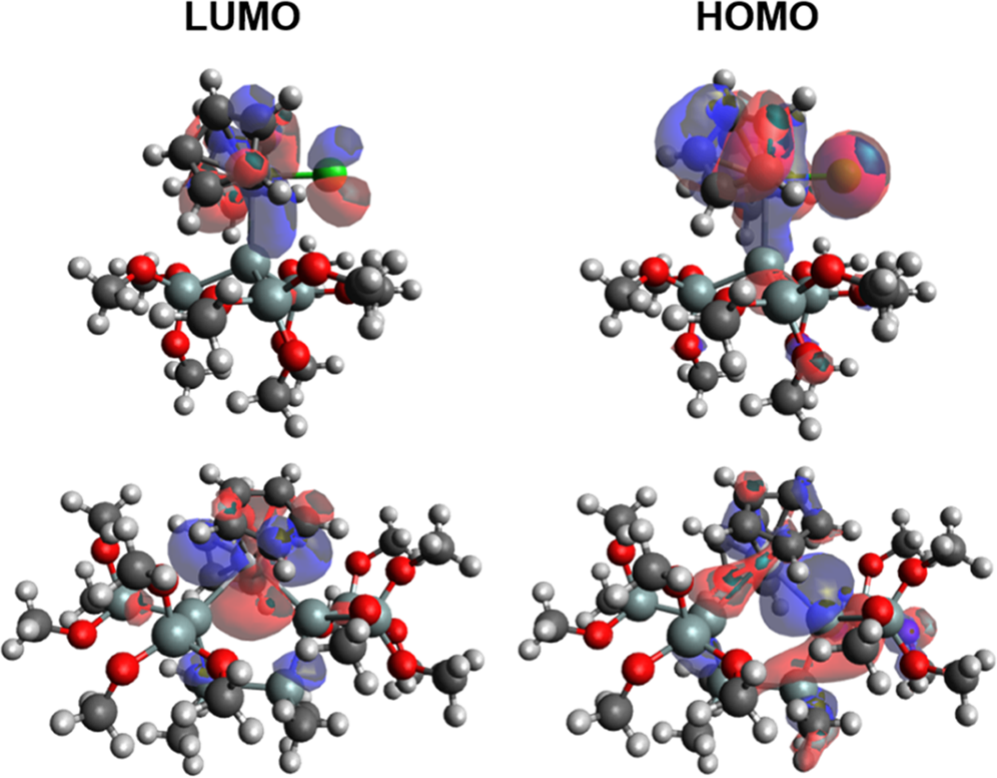
LUMO (left) and HOMO (right) of compounds **2** (above) and **9** (below).

To complete the characterization of the isolable silyl-substituted metallocenes **2**–**4** and **9**–**11**, we recorded their UV–vis absorption spectra and assigned them computationally. Figure 6a shows compounds **2**–**4**, while Figure 6b depicts compounds **9**–**11**. A qualitative agreement between calculated and experimental absorption maxima could be achieved for all bands (compare Supporting Information). All longest wavelength absorptions are simple highest occupied molecular orbital–lowest unoccupied molecular orbital (HOMO–LUMO) transitions. As shown in Figure 7, the HOMO orbitals for compounds **2**–**4** are delocalized over the organic as well as inorganic substituents. The LUMO of **2**–**4** has predominantly d orbital character. Interestingly, the HOMO orbitals for compounds **9**–**11** are mainly delocalized over the silicon heterocycles, while the LUMO orbitals still have a high d character.

## Conclusions

In conclusion, new methoxylated oligosilyl-substituted metallocenes were synthesized by the reaction of two oligosilanides with metallocene dichlorides (M = Ti, Zr, and Hf) in good to excellent yields. The monosubstituted metallocenes **2**–**4** were characterized via NMR, UV–vis spectroscopy, and X-ray crystallography. Especially interesting was the radical compound **5**, which was obtained by the failed attempt to bis-silylate titanocene dichloride. Compound **5** was characterized via EPR spectroscopy and X-ray crystallography. By comparing the crystal structure of **2** and **5**, changes in bond lengths and angles were observable. Particularly interesting was the coordination of one methoxy group to the metal, which is the reason for the high stability of this radical compound. By investigating the reactivity of **5**, two quenching experiments (with TEMPO and (bromomethyl)benzene) were performed. Since the bis-silylation with **1c** was unsuccessful, another silanide **8** was synthesized. By the reaction of **8** with the same metallocene dichlorides, the formation of the heterocyclic compounds **9**–**11** was observed. Compounds **9**–**11** were fully characterized via NMR and UV–vis spectroscopies. Additionally, for compound **9**, crystals of sufficient quality for X-ray crystallography were obtained. Further studies to probe the scope for these molecules as catalysts are currently in progress.

## Experimental Section

### General Procedures

All experiments were performed under a nitrogen atmosphere using standard Schlenk techniques. Solvents were dried using a column solvent purification system.^44^ Commercial reagents were used as purchased, unless otherwise noted. ^1^H (299.95 MHz), ^13^C (75.43 MHz), and ^29^Si (59.59 MHz) NMR spectra were recorded on either a Varian INOVA 300 MHz spectrometer, Varian Mercury 300 MHz spectrometer, or a Bruker Avance III 300 MHz with an autosampler. Additionally, ^1^H (199.97 MHz) and ^29^Si (39.73 MHz) NMR spectra were also recorded on a 200 MHz Bruker AVANCE DPX spectrometer in C_6_D_6_ solution (99.5 atom %, D) using the internal ^2^H-lock signal of the solvent. (Si(OMe)_3_)_4_Si^45,46^ and 1,1,1,6,6,6-hexamethoxy-3,3,4,4-tetramethyl-2,2,5,5-tetrakis(trimethoxysilyl)hexasilane^34^ were synthesized according to published procedures. Mass spectra were acquired either with a Q-TOF Premier from Waters, Manchester, England. Therefore, the original ESI source of the instrument was replaced by a standard LIFDI source from Linden CMS, Weyhe, Germany. Infrared spectra were obtained on a Bruker Alpha-P Diamond ATR spectrometer from the solid sample. Elemental analyses were carried out on a Hanau Vario Element EL apparatus. Continuous-wave time-resolved EPR (TR-EPR) experiments were performed on a Bruker ESP 300E X-band spectrometer (unmodulated static magnetic field) equipped with a 125 MHz dual-channel digital oscilloscope (Le Croy 9400). UV absorption spectra were recorded on a PerkinElmer Lambda 5 spectrometer.

### X-ray Crystallography

All crystals suitable for single-crystal X-ray diffractometry were removed from a vial or Schlenk flask and immediately covered with a layer of silicone oil. A single crystal was selected, mounted on a glass rod on a copper pin, and placed in a cold N_2_ stream. X-ray diffraction (XRD) data collections for compounds **2**–**5**, **8**, and **9** were performed on a Bruker APEX II diffractometer with the use of an Incoatec microfocus sealed tube of Mo Kα radiation (λ = 0.71073 Å) and a charge-coupled device (CCD) area detector. Empirical absorption corrections were applied using SADABS or TWINABS.^47,48^ The structures were solved with either the use of direct methods or the intrinsic phasing option in SHELXT and refined by the full-matrix least-squares procedures in SHELXL^49−51^ or Olex2.^52^ The space group assignments and structural solutions were evaluated using PLATON.^53,54^ Nonhydrogen atoms were refined anisotropically. Hydrogen atoms were either located in a difference map or in calculated positions corresponding to standard bond lengths and angles. The disorder was handled by modeling the occupancies of the individual orientations using free variables to refine the respective occupancy of the affected fragments (PART).^55^Table S1 in the Supporting Information contains crystallographic data and details of measurements and refinement for all compounds. Crystallographic data (excluding structure factors) have been deposited with the Cambridge Crystallographic Data Centre (CCDC) under the following numbers: **2**, 2176007; **3**, 2176008; **4**, 2176009; **5**, 2176010; **8**, 2176011; **9**, 2176012.

### Density Functional Theory (DFT) Computations

The computations were performed in the gas phase with the Gaussian 16 revision C.01 program package at the B3LYP level and LANL2DZ basis set.^56^

### Synthesis of (η^5^-Dicyclopentadienyl)(1,1,1,3,3,3-hexamethoxy-2-(trimethoxysilyl)-tri-silan-2-yl)titanium(IV) Chloride (**2**)

Briefly, 0.23 g of KO^*t*^Bu (2.1 mmol) was added to a solution of 1.0 g of 1,1,1,3,3,3-hexamethoxy-2,2-bis(trimethoxysilyl)trisilane (2.0 mmol) in 25 mL of THF at 0 °C. After additional stirring for 45 min at RT, the reaction mixture was slowly added to a −30 °C solution of 0.51 g of [Cl_2_TiCp_2_] (2.1 mmol) in 25 mL of THF. The reaction mixture was brought to room temperature and stirred for an hour. Subsequently, the solvent was removed in vacuum. Fifty milliliters of *n*-pentane was added, and the salts were removed by filtration. A blue solid precipitate formed in *n*-pentane at −70 °C to obtain 0.80 g of **2** (68%). Anal. Calcd for C_19_H_37_ClO_9_Si_4_Ti: C, 37.71%; H, 6.16%. Found: C, 37.76%; H, 6.21%. ^1^H NMR (C_6_D_6_, ppm): 6.38 (s, 10H, C_5_*H*_5_); 3.68 (s, 27H, Si(OC*H*_3_)_3_). ^29^Si NMR (C_6_D_6_, ppm): −33.1 (s, *Si*(OCH_3_)_3_); −88.0 (s, *Si*(Si(OCH_3_)_3_)_3_). ^13^C NMR (C_6_D_6_, ppm): 114.98 (s, *C*_5_H_5_); 50.50 (s, Si(O*C*H_3_)_3_). IR (v [cm^–1^]): 3116, 2940 (m, C_5_H_5_); 2834 (s, OCH_3_); 774, 711, 693 (m, Si–Si). UV–vis measured in *n*-hexane with *c* = 1 × 10^–3^ mol/L (γ [nm], ε [L mol^–1^ cm^–1^]): 3820; 617. High-resolution mass spectrometry (HRMS) (LIFDI^+^) calcd for [C_19_H_37_ClO_9_Si_4_Ti]^+•^ (M^+^): 604.0686. Found: 604.1290.

### Synthesis of (η^5^-Dicyclopentadienyl)(1,1,1,3,3,3-hexamethoxy-2-(trimethoxysilyl)tri-silan-2-yl)zirconium(IV) Chloride (**3**)

Briefly, 0.23 g of KO^*t*^Bu (2.1 mmol) was added to a solution of 1.0 g of 1,1,1,3,3,3-hexamethoxy-2,2-bis(trimethoxysilyl)trisilane (2.0 mmol) in 25 mL of THF at 0 °C. After additional stirring for 45 min at RT, the reaction mixture was slowly added to a −30 °C solution of 0.60 g of [Cl_2_ZrCp_2_] (2.1 mmol) in 25 mL of 1,2-dimethoxyethane (DME). The reaction mixture was brought to room temperature and stirred for an hour. The solvent was removed in vacuum. Fifty milliliters of *n*-pentane was added, and the salts were removed by filtration. A yellow solid precipitate formed in *n*-pentane at −70 °C to obtain 0.78 g of **3** (62%). Anal. Calcd for C_19_H_37_ClO_9_Si_4_Zr: C, 35.19%; H, 5.75%, found: C, 35.24%; H, 5.72%. ^1^H NMR (C_6_D_6_, ppm): 6.36 (s, 10H, C_5_*H*_5_); 3.67 (s, 27H, Si(OC*H*_3_)_3_). ^29^Si NMR (C_6_D_6_, ppm): −29.3 (s, *Si*(OCH_3_)_3_); −128.0 (s, *Si*(Si(OCH_3_)_3_)_3_). ^13^C NMR (C_6_D_6_, ppm): 112.86 (s, *C*_5_H_5_); 50.36 (s, Si(O*C*H_3_)_3_). IR (v [cm^–1^]) 3109, 2943 (m, C5H5); 2834 (s, OCH_3_); 792, 711, 683 (m, Si–Si). UV–vis measured in *n*-hexane with *c* = 1 × 10^–3^ mol/L (γ [nm], ε [L mol^–1^ cm^–1^]): 12267; 759. HRMS (LIFDI^+^) calcd for [C_18_H_34_ClO_9_Si_4_Zr + H]^+^ (M–CH_2_): 632.0094. Found: 632.0720.

### Synthesis of (η^5^-Dicyclopentadienyl)(1,1,1,3,3,3-hexamethoxy-2-(trimethoxysilyl)tri-silan-2-yl)hafnium(IV) Chloride (**4**)

Briefly, 0.23 g of KO^*t*^Bu (2.1 mmol) was added to a solution of 1.0 g of 1,1,1,3,3,3-hexamethoxy-2,2-bis(trimethoxysilyl)trisilane (2.0 mmol) in 25 mL of THF at 0 °C. After additional stirring for 45 min at RT, the reaction mixture was slowly added to a −30 °C solution of 0.78 g of [Cl_2_HfCp_2_] (2.1 mmol) in 25 mL of DME. The reaction mixture was brought to room temperature and stirred for an hour. Fifty milliliters of *n*-pentane was added, and the salts were removed by filtration. An orange solid precipitate formed in *n*-pentane at −70 °C to obtain 0.92 g of **4** (64%). Anal. Calcd for C_19_H_37_ClHfO_9_Si_4_: C, 31.02%; H, 5.07%. Found: C, 31.24%; H, 5.24%. ^1^H NMR (C_6_D_6_, ppm): 6.28 (s, 10H, C_5_*H*_5_); 3.70 (s, 27H, Si(OC*H*_3_)_3_). ^29^Si NMR (C_6_D_6_, ppm): −27.2 (s, *Si*(OCH_3_)_3_); −117.4 (s, *Si*(Si(OCH_3_)_3_)_3_). ^13^C NMR (C_6_D_6_, ppm): 111.76 (s, *C*_5_H_5_); 50.43 (s, Si(O*C*H_3_)_3_). IR (v [cm^–1^]): 3108, 2939 (m, C_5_H_5_); 2836 (s, OCH_3_); 778, 711, 686 (m, Si–Si). UV–vis measured in *n*-hexane with *c* = 1 × 10^–3^ mol/L (γ [nm], ε [L mol^–1^ cm^–1^]): 11737; 1296. HRMS (LIFDI^+^) calcd for [C_18_H_34_ClO_9_Si_4_Hf + H]^+^ (M–CH_2_): 722.0512. Found: 722.1219.

### Synthesis of (η^5^-Dicyclopentadienyl)(1,1,1,3,3,3-hexamethoxy-2-(trimethoxysilyl)tri-silan-2-yl)titanium(III) (**5**)

#### Method a

Briefly, 0.23 g of KO^*t*^Bu (2.1 mmol) was added to a solution of 1.0 g of 1,1,1,3,3,3-hexamethoxy-2,2-bis(trimethoxysilyl)trisilane (2.0 mmol) in 25 mL of THF at 0 °C. After additional stirring for 45 min at RT, the reaction mixture was slowly added to a 0 °C solution of 0.26 g [Cl_2_TiCp_2_] (1.0 mmol) in 25 mL of THF. The reaction mixture was brought to room temperature and stirred for an hour. The color changed from blue to green. The solvent was removed in vacuum. Fifty milliliters of *n*-pentane was added, and the salts were removed by filtration. A green solid precipitate was formed in *n*-pentane at −30 °C to obtain 0.33 g of **5** (60%).

#### Method b

Briefly, 0.50 g of (η^5^-dicyclopentadienyl)(1,1,1,3,3,3-hexamethoxy-2-(trimethoxysilyl)-tri-silan-2-yl)titanium(IV) chloride (0.83 mmol), 0.12 g of KC_8_ (0.87 mmol), or 0.33 g of the magnesium(I)dimer [{(MesNacnac)Mg−}_2_] (0.45 mmol) was stirred in 25 mL of THF overnight. The solvent was removed in vacuum. Thirty milliliters of *n*-pentane was added, and the salts were removed by filtration. A green solid precipitate formed in *n*-pentane at −30 °C to obtain 0.18 g of **5** (70%, identical for both reduction agents). Anal. Calcd for C_19_H_37_O_9_Si_4_Ti^•^: C, 40.06%; H, 6.55%. Found: C, 39.88%; H, 6.78%. UV–vis measured in *n*-hexane with *c* = 1 × 10^–4^ mol/L (γ [nm], ε [L mol^–1^ cm^–1^]): 16 754; 12 945; 357.

### Synthesis of (η^5^-Dicyclopentadienyl)(1,1,1,3,3,3-hexamethoxy-2-(trimethoxysilyl)tri-silan-2-yl)((2,2,6,6-tetramethylpiperidin-1-yl)oxy)titanium (**6**)

Briefly, 1.0 g of **5** (1.8 mmol) and 0.29 g of TEMPO (1.8 mmol) were stirred in 25 mL of THF for 1 h at RT. The solvent was removed in vacuum. The red product precipitates in *n*-pentane at −30 °C to obtain 1.0 g of **6** (80%). Anal. Calcd for C_28_H_55_NO_10_Si_4_Ti: C, 46.33%; H, 7.64%. Found: C, 46.44%; H, 7.76%. ^1^H NMR (C_6_D_6_, ppm): 6.33 (s, 10H, C_5_*H*_5_), 3.71 (s, 27H, Si(OC*H*_3_)_3_); 1.46 (s, 6 H; C*H*_2_); 1.09 (s, 12 H; C*H*_3_). ^29^Si NMR (C_6_D_6_, ppm): −30.3 (s, *Si*(OCH_3_)_3_); −113.7 (s, *Si*(Si(OCH_3_)_3_)_3_). ^13^C NMR (C_6_D_6_, ppm): 112.11 (s, *C*_5_H_5_); 60.51 (s, *C*(CH_3_)_2_); 50.49 (s, Si(O*C*H_3_)_3_); 41.01 (s, *C*H_2_); 32.10 (s, *C*H_3_); 17.24 (s, *C*H_2_). IR (v [cm^–1^]): 3116, 2933 (m, C_5_H_5_); 2838 (s, OCH_3_); 1450 (m, CH_2_); 1373 (m, CH_3_); 771, 683, 658 (m, Si–Si). UV–vis measured in *n*-hexane with *c* = 1 × 10^–4^ mol/L (γ [nm], ε [L mol^–1^ cm^–1^]): 25 206; 18 312; 1545.

### Synthesis of (η^5^-Dicyclopentadienyl)(1,1,1,3,3,3-hexamethoxy-2-(trimethoxysilyl)tri-silan-2-yl)titanium(IV) Bromide (**7**)

Briefly, 1.0 g of **5** (1.8 mmol) and 0.22 mL (bromomethyl)benzene (1.8 mmol) were stirred in 25 mL of THF for 15 min at RT. The solvent was removed in vacuum. The blue product precipitates in *n*-pentane at −30 °C to obtain 0.97 g of **7** (85%). Anal. Calcd for C_19_H_37_BrO_9_Si_4_Ti: C, 35.13%; H, 5.74%. Found: C, 35.12%; H, 5.75%. ^1^H NMR (C_6_D_6_, ppm): 6.43 (s, 10H, C_5_*H*_5_). 3.67 (s, 27H, Si(OC*H*_3_)_3_). ^29^Si NMR (C_6_D_6_, ppm): −34.2 (s, *Si*(OCH_3_)_3_); −81.2 (s, *Si*(Si(OCH_3_)_3_)_3_). ^13^C NMR (C_6_D_6_, ppm): 115.05 (s, *C*_5_H_5_); 50.54 (s, Si(O*C*H_3_)_3_). IR (v [cm^–1^]): 3114, 2941 (m, C_5_H_5_); 2832 (s, OCH_3_); 775, 711, 691 (m, Si–Si). UV–vis measured in *n*-hexane with *c* = 1 × 10^–4^ mol/L (γ [nm], ε [L mol^–1^ cm^–1^]): 15 201; 434.

### Synthesis of (η^5^-Dicyclopentadienyl)-2,2,5,5-tetrakis(trimethoxysilyl)tetramethoxy-1-titana-cyclopentasilane (**9**)

Briefly, 0.13 g of KO^*t*^Bu (1.1 mmol) was added to a solution of 0.50 g of 1,1,1,6,6,6-hexamethoxy-3,3,4,4-tetramethyl-2,2,5,5-tetrakis(trimethoxysilyl)hexasilane (0.56 mmol) in 20 mL of THF at 0 °C. After additional stirring for 45 min at RT, the reaction mixture was slowly added to a −30 °C solution of 0.15 g of [Cl_2_TiCp_2_] (0.58 mmol) in 15 mL of *n*-pentane. The mixture was stirred for an hour at RT. The solvent was removed in vacuum. Thirty milliliters of *n*-pentane was added, and the salts were removed by filtration. A dark red solid precipitates in *n*-pentane at −70 °C to obtain 0.18 g of **9** (44%). Anal. Calcd for C_26_H_58_O_4_Si_8_Ti: C, 44.15%; H, 8.27%. Found: C, 44.36%; H, 8.35%. ^1^H NMR (C_6_D_6_, ppm): 6.99 (s, 10H, C_5_*H*_5_); 3.59 (s, 36H, Si(OC*H*_3_)_3_); 0.76 (s, 12H, Si(C*H*_3_)_2_). ^29^Si NMR (C_6_D_6_, ppm): −7.8 (s, *Si*(CH_3_)_2_); −30.7 (s, *Si*(OCH_3_)_3_); −57.6 (s, *Si*(Si(OCH_3_)_3_)_2_). ^13^C NMR (C_6_D_6_, ppm): 114.82 (s, *C*_5_H_5_); 50.21 (s, Si(O*C*H_3_)_3_); 1.29 (s, Si(*C*H_3_)_2_). IR (v [cm^–1^]): 3116, 2929 (m, C_5_H_5_); 2835 (s, OCH_3_), 1450 (m, CH_3_); 1059 (s, CH_3_); 771, 683, 650 (m, Si–Si). UV–vis measured in *n*-hexane with *c* = 1 × 10^–4^ mol/L (γ [nm], ε [L mol^–1^ cm^–1^]): 17972; 2486; 2539. HRMS (LIFDI^+^) calcd for [C_26_H_58_O_12_Si_8_Ti]^+•^ (M^+•^): 834.1567. Found: 834.1490.

### Synthesis of (η^5^-Dicyclopentadienyl)-2,2,5,5-tetrakis(trimethoxysilyl)tetramethyl-1-zirconacyclopentasilane (**10**)

Briefly, 0.13 g of KO^*t*^Bu (1.1 mmol) was added to a solution of 0.50 g of 1,1,1,6,6,6-hexamethoxy-3,3,4,4-tetramethyl-2,2,5,5-tetrakis(trimethoxysilyl)hexasilane (0.56 mmol) in 20 mL of THF at 0 °C. After additional stirring for 45 min at RT, the reaction mixture was slowly added to a 0 °C solution of 0.17 g of [Cl_2_ZrCp_2_] (0.58 mmol) in 15 mL of DME. The mixture was stirred for an hour at RT. The solvent was removed in vacuum. Thirty milliliters of *n*-pentane was added, and the salts were removed by filtration. A dark orange solid precipitates in *n*-pentane at −70 °C to obtain 0.17 g of **10** (40%). Anal. Calcd for C_26_H_58_O_4_Si_8_Zr: C, 41.60%; H, 7.79% Found: C, 41.96%; H, 7.86%. ^1^H NMR (C_6_D_6_, ppm): 6.82 (s, 10H, C_5_*H*_5_); 3.63 (s, 36H, Si(OC*H*_3_)_3_); 0.75 (s, 12H, Si(C*H*_3_)_2_). ^29^Si NMR (C_6_D_6_, ppm): −15.4 (s, *Si*(CH_3_)_2_); −26.6 (s, *Si*(OCH_3_)_3_); −94.6 (s, *Si*(Si(OCH_3_)_3_)_2_). ^13^C NMR (C_6_D_6_, ppm): 111.16 (s, *C*_5_H_5_); 50.18 (s, Si(O*C*H_3_)_3_); 0.85 (s, Si(*C*H_3_)_2_). IR (v [cm^–1^]): 3112, 2935 (m, C_5_H_5_); 2833 (s, OCH_3_), 1454 (m, CH_3_); 1058 (s, CH_3_); 770, 678, 637 (m, Si–Si). UV–vis measured in *n*-hexane with *c* = 1 × 10^–4^ mol/L (γ [nm], ε [L mol^–1^ cm^–1^]): 1091; 1507; 228. HRMS (LIFDI^+^) calcd for [C_26_H_58_O_12_Si_8_Zr]^+•^ (M^+•^): 876.1130. Found: 876.0469.

### Synthesis of (η^5^-Dicyclopentadienyl)-2,2,5,5-tetrakis(trimethoxysilyl)tetramethyl-1-hafna-cyclopentasilane (**11**)

Briefly, 0.13 g of KO^*t*^Bu (1.1 mmol) was added to a solution of 0.50 g of 1,1,1,6,6,6-hexamethoxy-3,3,4,4-tetramethyl-2,2,5,5-tetrakis-(trimethoxysilyl)hexasilane (0.56 mmol) in 20 mL of THF at 0 °C. After additional stirring for 45 min at RT, the reaction mixture was slowly added to a −30 °C solution of 0.22 g [Cl_2_HfCp_2_] (0.58 mmol) in 15 mL of *n-*pentane. The mixture was stirred for an hour at RT. The solvent was removed via vacuum. Fifty milliliters of *n*-pentane was added, and the salts were removed by filtration. An orange-red solid precipitates in *n*-pentane at −70 °C to obtain 0.17 g of **11** (40%). Anal. Calcd for C_26_H_58_HfO_4_Si_8_: C, 37.27%; H, 6.98%. Found: C, 37.39%; H, 7.21%. ^1^H NMR (C_6_D_6_, ppm): 6.68 (s, 10H, C_5_*H*_5_); 3.61 (s, 36H, Si(OC*H*_3_)_3_); 0.71 (s, 12H, Si(C*H*_3_)_2_). ^29^Si NMR (C_6_D_6_, ppm): −16.4 (s, *Si*(CH_3_)_2_); −23.8 (s, *Si*(OCH_3_)_3_); −75.7 (s, *Si*(Si(OCH_3_)_3_)_2_). ^13^C NMR (C_6_D_6_, ppm): 110.34 (s, *C*_5_H_5_); 50.21 (s, Si(O*C*H_3_)_3_); 1.11 (s, Si(*C*H_3_)_2_). IR (v [cm^–1^]): 3123, 2940 (m, C_5_H_5_); 2838 (s, OCH_3_), 1457 (m, CH_3_); 1054 (s, CH_3_); 770, 675, 642 (m, Si–Si). UV–vis measured in *n*-hexane with *c* = 1 × 10^–4^ mol/L (γ [nm], ε [L mol^–1^ cm^–1^]): 1316; 1707; 185. HRMS (LIFDI^+^) calcd for [C_26_H_58_O_12_Si_8_Hf]^+•^ (M^+•^): 966.1539. Found: 966.0867.

## Supporting Information Available

^1^H, ^13^C, and ^29^Si NMR spectra for all isolated compounds; UV–vis spectra of **5**–**7**; X-ray data of all structural characterized compounds; and density functional theory computations (PDF)
