# Supplementary material for: Synthesis and Characterization of Methoxylated Oligosilyl Group 4 Metallocenes
Source: Inorg Chem. 2022 Sep 1;61(37):14742–51. doi: 10.1021/acs.inorgchem.2c02112 (PMC9490756; doi:10.1021/acs.inorgchem.2c02112)
Supplement: Supplementary file 1 — ic2c02112_si_001.pdf [file ic2c02112_si_001.pdf]

Supporting Information

Synthesis and Characterization of Methoxylated Oligosilyl Group 4 Metallocenes

Aileen Sauermoser,[a] Thomas Lainer,[a] Gabriel Glotz,[b] Frank Czerny,[c] Bettina Schweda,[d] Roland C. Fischer,[a] Michael Haas[a]*

[a] Institute of Inorganic Chemistry, Graz University of Technology; Stremayrgasse 9/V, 8010 Graz (Austria); E-mail: michael.haas@tugraz.at

[b] Institute of Physical and Theoretical Chemistry, Graz University of Technology; Stremayrgasse 9/II, 8010 Graz (Austria)

[c] Department of Chemistry: Metalorganics and Inorganic Materials, Berlin University of Technology, 10623 Berlin, Germany

[d] Institute for Chemistry and Technology of Materials, Graz University of Technology; Stremayrgasse 9/IV, 8010 Graz (Austria)

Table of Content

Analytical Section 4

NMR-Spectroscopy 4

Figure S1: ^{1}H-NMR spectra of (η^{5}-dicyclopentadienyl)(1,1,1,3,3,3-hexamethoxy-2-(trimethoxysilyl)trisilan-2-yl)titanium(IV) chloride (**2**) (C_6D_6 solution, ppm, 299.95 MHz)......................4

Figure S2: ^{29}Si-NMR spectra of (η^{5}-dicyclopentadienyl)(1,1,1,3,3,3-hexamethoxy-2-(trimethoxysilyl)trisilan-2-yl)titanium(IV) chloride (**2**) (C_6D_6 solution, 39.73 MHz, RT, ppm).................5

Figure S3: ^{13}C-NMR spectra of (η^{5}-dicyclopentadienyl)(1,1,1,3,3,3-hexamethoxy-2-(trimethoxysilyl)trisilan-2-yl)titanium(IV) chloride (**2**) (C_6D_6 solution, 75.43 MHz, RT, ppm).................6

Figure S4: ^{1}H-NMR spectra of (η^{5}-dicyclopentadienyl)(1,1,1,3,3,3-hexamethoxy-2-(trimethoxysilyl)trisilan-2-yl)zirconium(IV) chloride (**3**) (C_6D_6 solution, 299.95 MHz, RT, ppm).............7

Figure S5: ^{29}Si-NMR spectra of (η^{5}-dicyclopentadienyl)(1,1,1,3,3,3-hexamethoxy-2-(trimethoxysilyl)trisilan-2-yl)zirconium(IV) chloride (**3**) (C_6D_6 solution, 39.73 MHz, RT, ppm)................8

Figure S6: ^{13}C-NMR spectra of (η^{5}-dicyclopentadienyl)(1,1,1,3,3,3-hexamethoxy-2-(trimethoxysilyl)trisilan-2-yl)zirconium(IV) chloride (**3**) (C_6D_6 solution, 75.43 MHz, RT, ppm)................9

Figure S7: ^{1}H-NMR spectra of (η^{5}-dicyclopentadienyl)(1,1,1,3,3,3-hexamethoxy-2-(trimethoxyysilyl)trisilan-2-yl)hafnium(IV) chloride (**4**) (C_6D_6 solution, 299.95 MHz, RT, ppm) 10

Figure S8: ^{29}Si-NMR spectra of (η^{5}-dicyclopentadienyl)(1,1,1,3,3,3-hexamethoxy-2-(trimethoxyysilyl)trisilan-2-yl)hafnium(IV) chloride (**4**) (C_6D_6 solution, 39.73 MHz, RT, ppm) 11

Figure S9: ^{13}C-NMR spectra of (η^{5}-dicyclopentadienyl)(1,1,1,3,3,3-hexamethoxy-2-(trimethoxyysilyl)trisilan-2-yl)hafnium(IV) chloride (**4**) (C_6D_6 solution, 75.43 MHz, RT, ppm) 12

Figure S10: ^{1}H-NMR spectra of (η^{5}-dicyclopentadienyl)(1,1,1,3,3,3-hexamethoxy-2-(trimethoxysilyl)trisilan-2-yl)((2,2,6,6-tetramethylpiperidin-1-yl)oxy)titanium (**6**) (C_6D_6 solution, 299.95 MHz, RT, ppm) 13

Figure S11: ^{29}Si-NMR spectra of (η^{5}-dicyclopentadienyl)(1,1,1,3,3,3-hexamethoxy-2-(trimethoxysilyl)trisilan-2-yl)((2,2,6,6-tetramethylpiperidin-1-yl)oxy)titanium (**6**) (C_6D_6 solution, 39.73 MHz, RT, ppm) 14

Figure S12: ^{13}C-NMR spectra of (η^{5}-dicyclopentadienyl)(1,1,1,3,3,3-hexamethoxy-2-(trimethoxysilyl)trisilan-2-yl)((2,2,6,6-tetramethylpiperidin-1-yl)oxy)titanium (**6**) (C_6D_6 solution, 75.43 MHz, RT, ppm) 15

Figure S13: ^{1}H-NMR spectra of (η^{5}-dicyclopentadienyl)(1,1,1,3,3,3-hexamethoxy-2-(trimethoxysilyl)trisilan-2-yl)titanium(IV) bromide (**7**) (C_6D_6 solution, 299.95 MHz, RT, ppm) 16

Figure S14: ^{29}Si-NMR spectra of (η^{5}-dicyclopentadienyl)(1,1,1,3,3,3-hexamethoxy-2-(trimethoxysilyl)trisilan-2-yl)titanium(IV) bromide (**7**) (C_6D_6 solution, 39.73 MHz, RT, ppm) 17

Figure S15: ^{13}C-NMR spectra of (η^{5}-dicyclopentadienyl)(1,1,1,3,3,3-hexamethoxy-2-(trimethoxysilyl)trisilan-2-yl)titanium(IV) bromide (**7**) (C_6D_6 solution, 75.43 MHz, RT, ppm) 18

Figure S16: ^{1}H-spectra of 2,2,3,3-tetramethyl-1,1,4,4-tetra(trimethoxysilyl)-1,4-tetradisilanide (**8**) (C_6D_6 solution, 199.97 MHz, RT, ppm)..19

Figure S17: ^{29}Si-NMR spectra of 2,2,3,3-tetramethyl-1,1,4,4-tetra(trimethoxysilyl)-1,4-tetradisilanide (**8**) (C_6D_6 solution, 39.73 MHz, RT, ppm)..20

Figure S18: ^{1}H-NMR spectra of (η^5-dicyclopentadienyl)-2,2,5,5-tetrakis (trimethoxysilyl)tetramethoxy-1-titanacyclopentasilane (**9**) (C_6D_6 solution, 299.95 MHz, RT, ppm) 21

Figure S19: ^{29}Si-NMR spectra of (η^5-dicyclopentadienyl)-2,2,5,5-tetrakis (trimethoxysilyl)tetramethoxy-1-titanacyclopentasilane (**9**) (C_6D_6 solution, 39.73 MHz, RT, ppm)......22

Figure S20: ^{13}C-NMR spectra of (η^5-dicyclopentadienyl)-2,2,5,5-tetrakis (trimethoxysilyl)tetramethoxy-1-titanacyclopentasilane (**9**) (C_6D_6 solution, , 75.43 MHz, RT, ppm)....23

Figure S24: ^{1}H-NMR spectra of (η^5-dicyclopentadienyl)-2,2,5,5-tetrakis (trimethoxysilyl)tetramethoxy-1-hafnacyclopentasilane (C_6D_6 solution, 299.95 MHz, RT, ppm) 27

Figure S25: ^{29}Si-NMR spectra of (η^5-dicyclopentadienyl)-2,2,5,5-tetrakis (trimethoxysilyl)tetramethoxy-1-hafnacyclopentasilane (C_6D_6 solution, 39.73 MHz, RT, ppm) 28

Figure S26: ^{13}C-NMR spectra of (η^5-dicyclopentadienyl)-2,2,5,5-tetrakis (trimethoxysilyl)tetramethoxy-1-hafnacyclopentasilane (C_6D_6 solution, 75.43 MHz, RT, ppm) 29

UV-Vis-Spectroscopy 30

Figure S27: UV-Vis spectra of compound **5** (c = 1*10^{-4} mol/L; solvent: *n*-hexane) 30

Figure S28 UV-Vis spectra of compound **6** (c = 1*10^{-4} mol/L; solvent: *n*-hexane) 31

Figure S29: UV-Vis spectra of compounds **7** (c = 1*10^{-4} mol/L; solvent: *n*-hexane)....32

Crystal Structures 33

Figure S30: ORTEP for compound **3**. 33

Figure S31: ORTEP for compound **4**. 34

Table S1: Crystallographic data of compounds **2**, **3**, **4**, **5, 8,** and **9**....35

Density Functional Theory Computations 36

Figure S32: Calculated absorption spectrum for compound **2**....36

Figure S33: HOMO and LUMO for compound **3**....37

Figure S34: Calculated absorption spectrum for compound **3**....37

Figure S35: HOMO and LUMO for compound **4**....38

Figure S36: Calculated absorption spectrum for compound **4**....38

Figure S37 Calculated absorption spectrum for compound **9**....39

Figure S38: HOMO and LUMO for compound **10**....39

Figure S39: Calculated absorption spectrum for compound **10**....40

Figure S40: HOMO and LUMO for compound **11**....40

Figure S41: Calculated absorption spectrum for compound **11**....41

Analytical Section

NMR-Spectroscopy

Figure S1: ^{1}H-NMR spectra of (η^{5}-dicyclopentadienyl)(1,1,1,3,3,3-hexamethoxy-2-(trimethoxysilyl)trisilan-2-yl)titanium(IV) chloride (**2**) (C_6D_6 solution, ppm, 299.95 MHz)

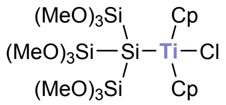

2

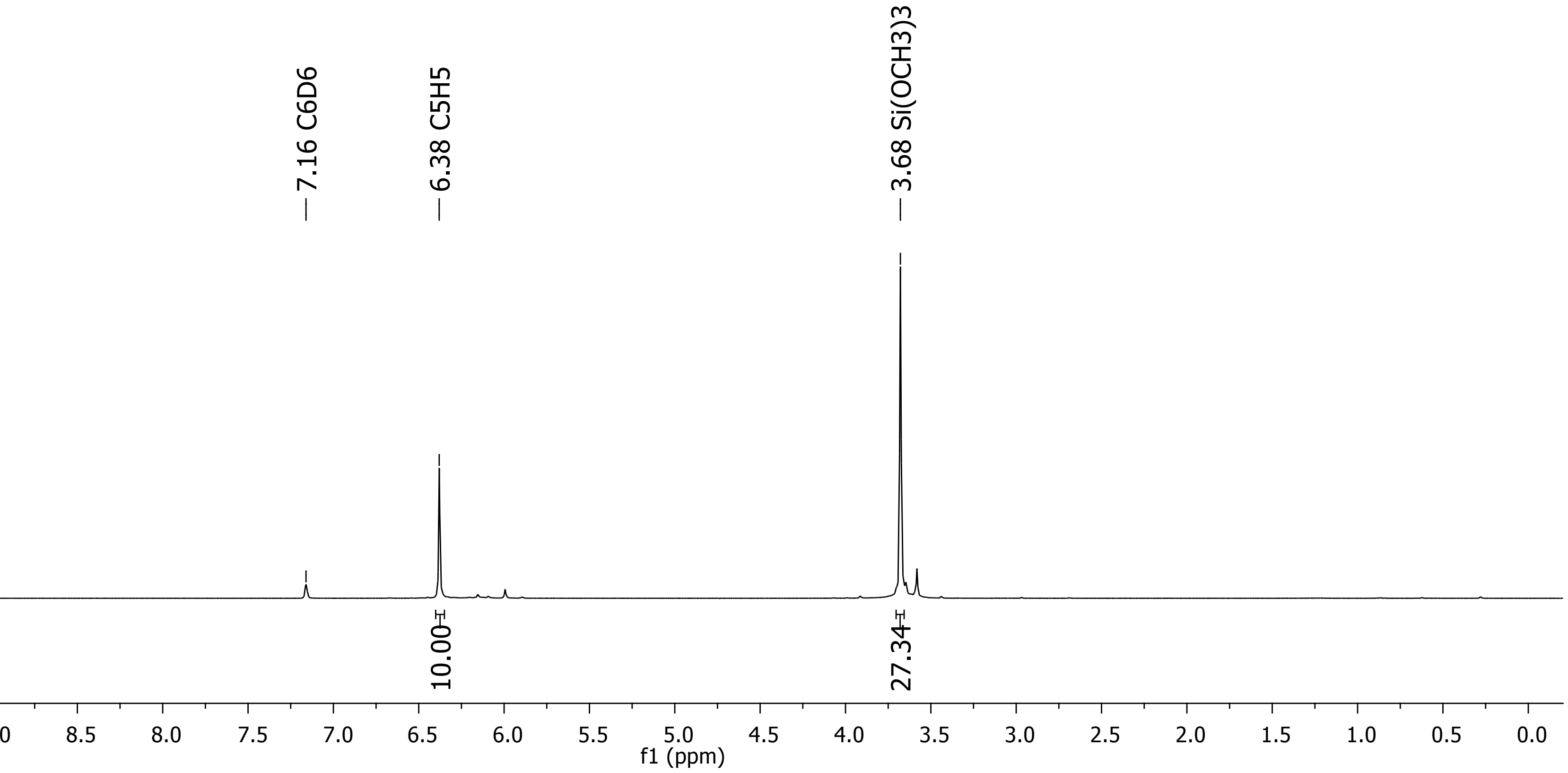

Figure S2: ^{29}Si-NMR spectra of (η^5-dicyclopentadienyl)(1,1,1,3,3,3-hexamethoxy-2-(trimethoxysilyl)trisilan-2-yl)titanium(IV) chloride (**2**) (C_6D_6 solution, 39.73 MHz, RT, ppm)

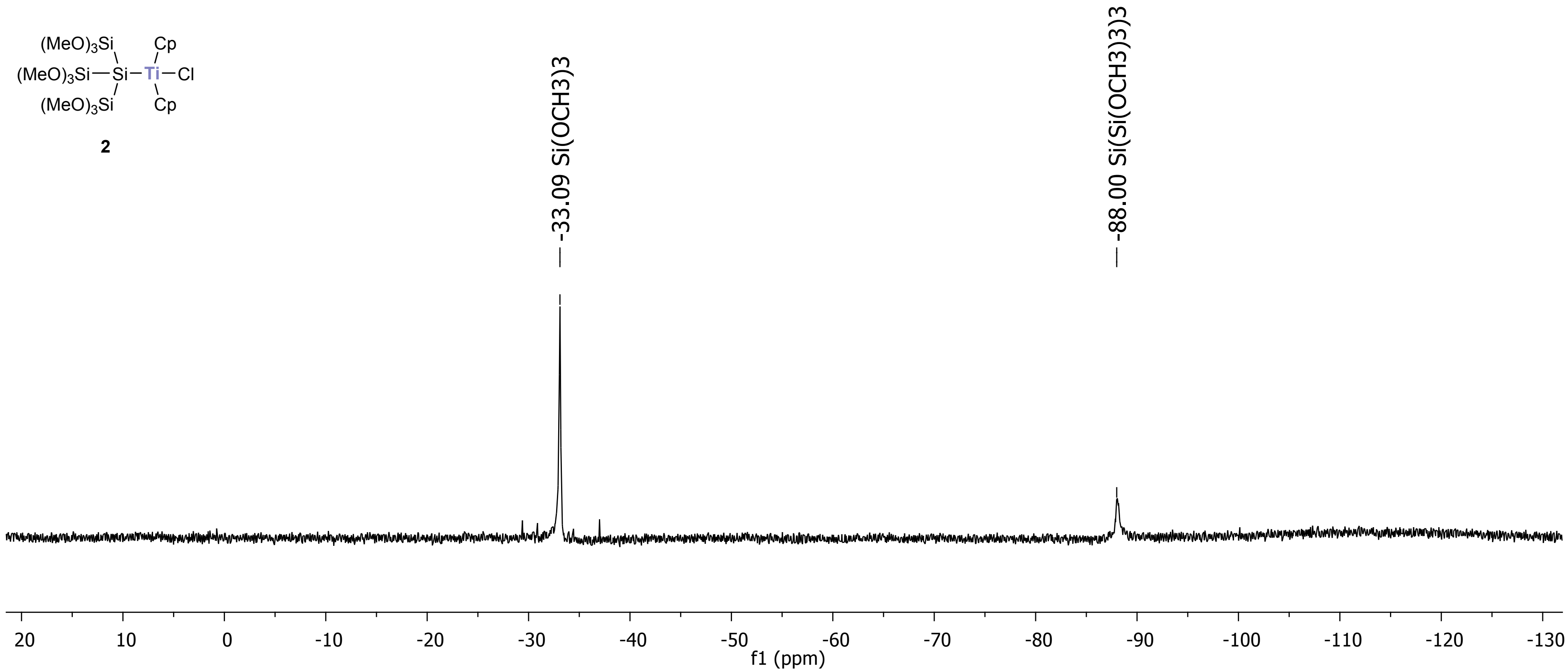

Figure S3: ^{13}C-NMR spectra of (η^5-dicyclopentadienyl)(1,1,1,3,3,3-hexamethoxy-2-(trimethoxysilyl)trisilan-2-yl)titanium(IV) chloride (**2**) (C_6D_6 solution, 75.43 MHz, RT, ppm)

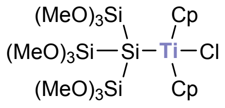

2

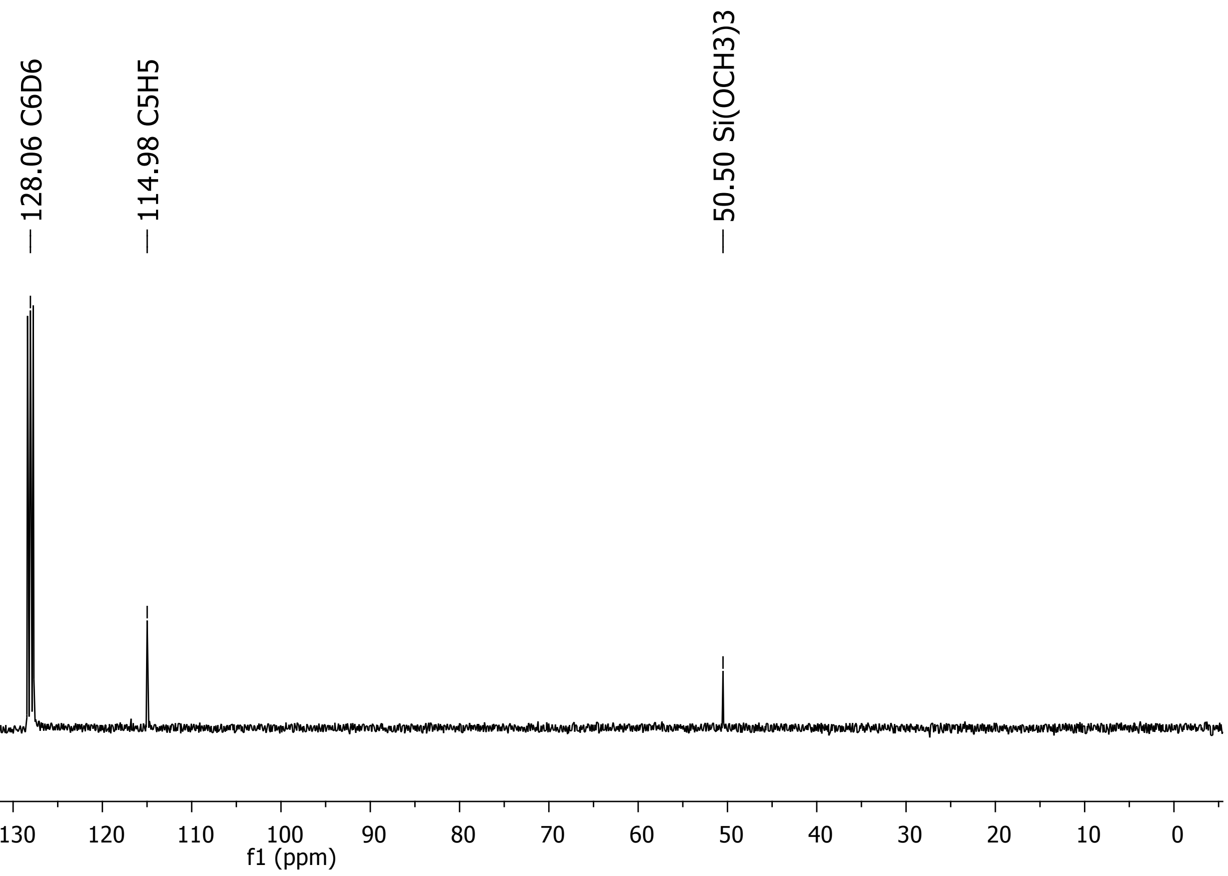

Figure S4: ^{1}H-NMR spectra of (η^{5}-dicyclopentadienyl)(1,1,1,3,3,3-hexamethoxy-2-(trimethoxysilyl)trisilan-2-yl)zirconium(IV) chloride (**3**) (C_6D_6 solution, 299.95 MHz, RT, ppm)

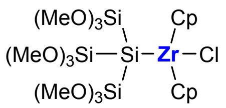

3

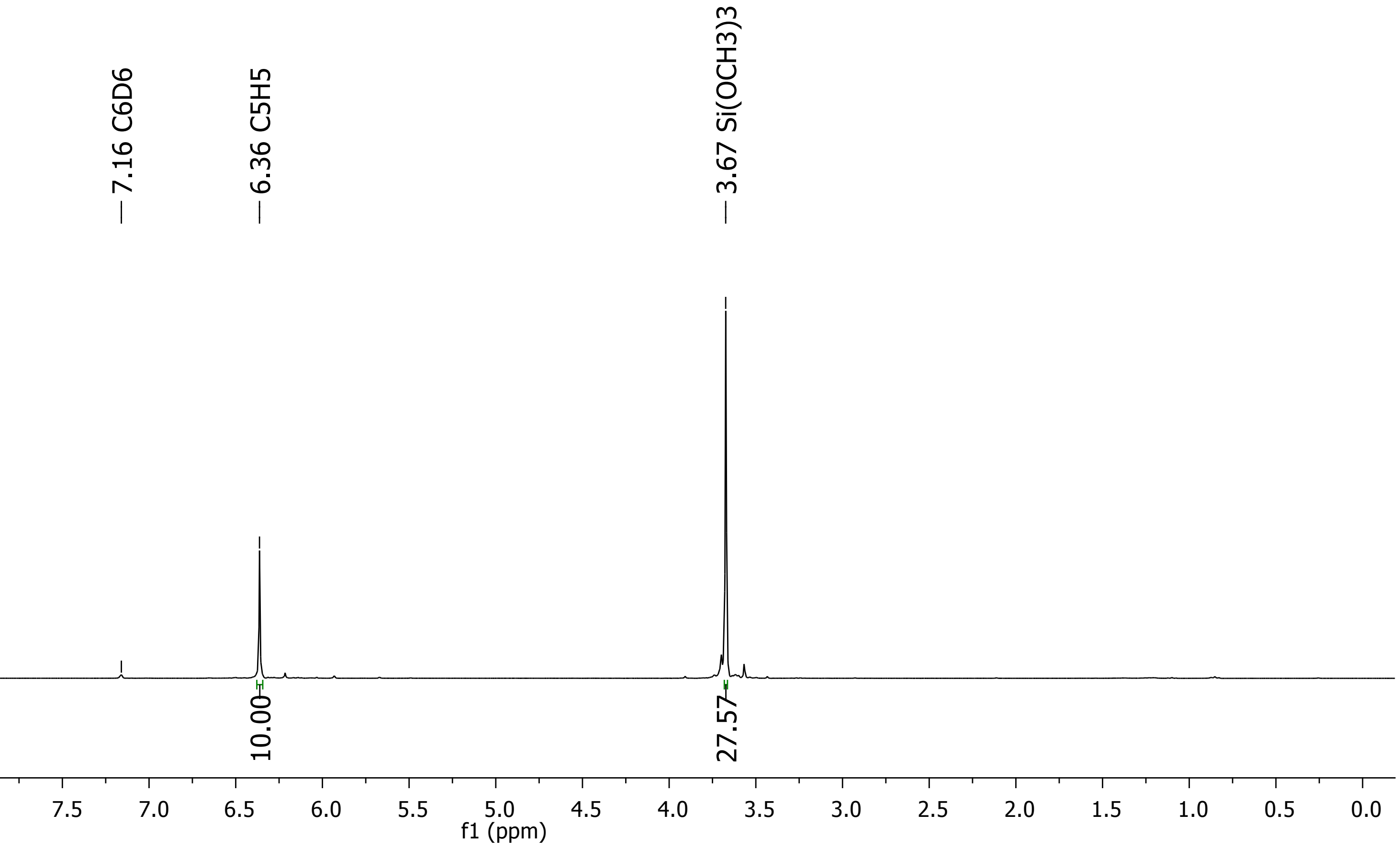

Figure S5: ^{29}Si-NMR spectra of (η^5-dicyclopentadienyl)(1,1,1,3,3,3-hexamethoxy-2-(trimethoxysilyl)trisilan-2-yl)zirconium(IV) chloride (**3**) (C_6D_6 solution, 39.73 MHz, RT, ppm)

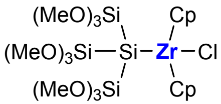

3

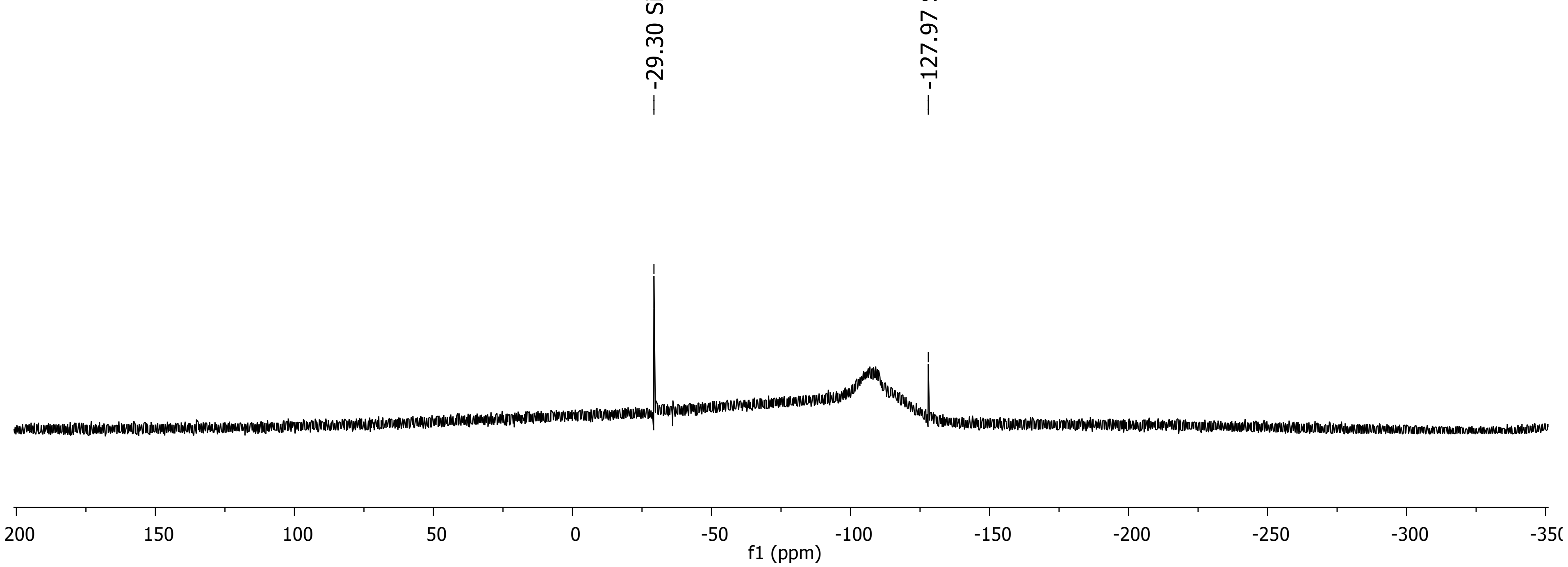

Figure S6: ^{13}C-NMR spectra of (η^5-dicyclopentadienyl)(1,1,1,3,3,3-hexamethoxy-2-(trimethoxysilyl)trisilan-2-yl)zirconium(IV) chloride (**3**) (C_6D_6 solution, 75.43 MHz, RT, ppm)

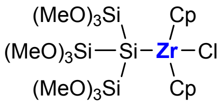

3

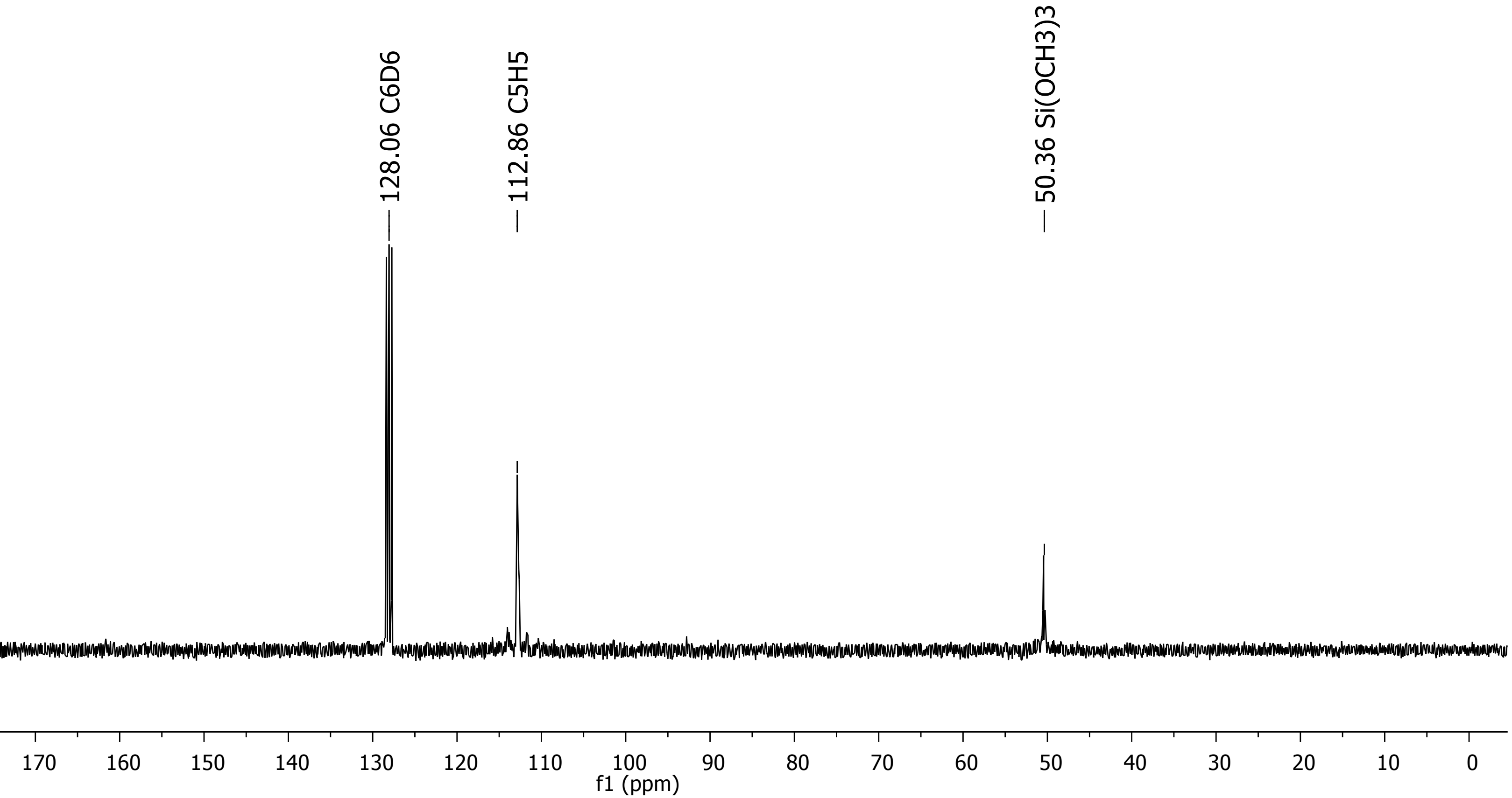

Figure S7: ^{1}H-NMR spectra of (η^5-dicyclopentadienyl)(1,1,1,3,3,3-hexamethoxy-2-(trimethoxyysilyl)trisilan-2-yl)hafnium(IV) chloride (**4**) (C_6D_6 solution, 299.95 MHz, RT, ppm)

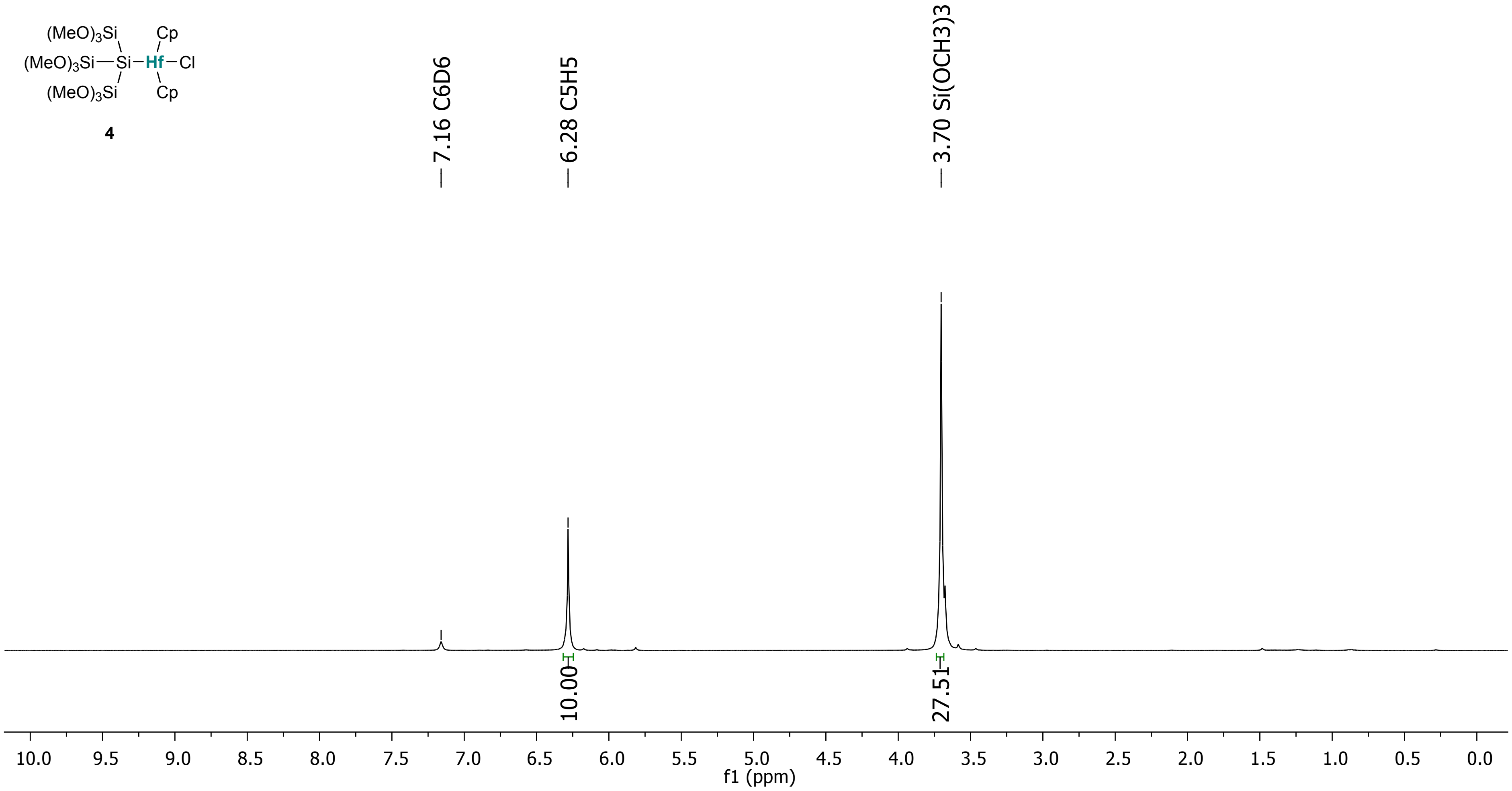

Figure S8: ^{29}Si-NMR spectra of (η^5-dicyclopentadienyl)(1,1,1,3,3,3-hexamethoxy-2-(trimethoxyysilyl)trisilan-2-yl)hafnium(IV) chloride (**4**) (C_6D_6 solution, 39.73 MHz, RT, ppm)

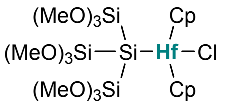

4

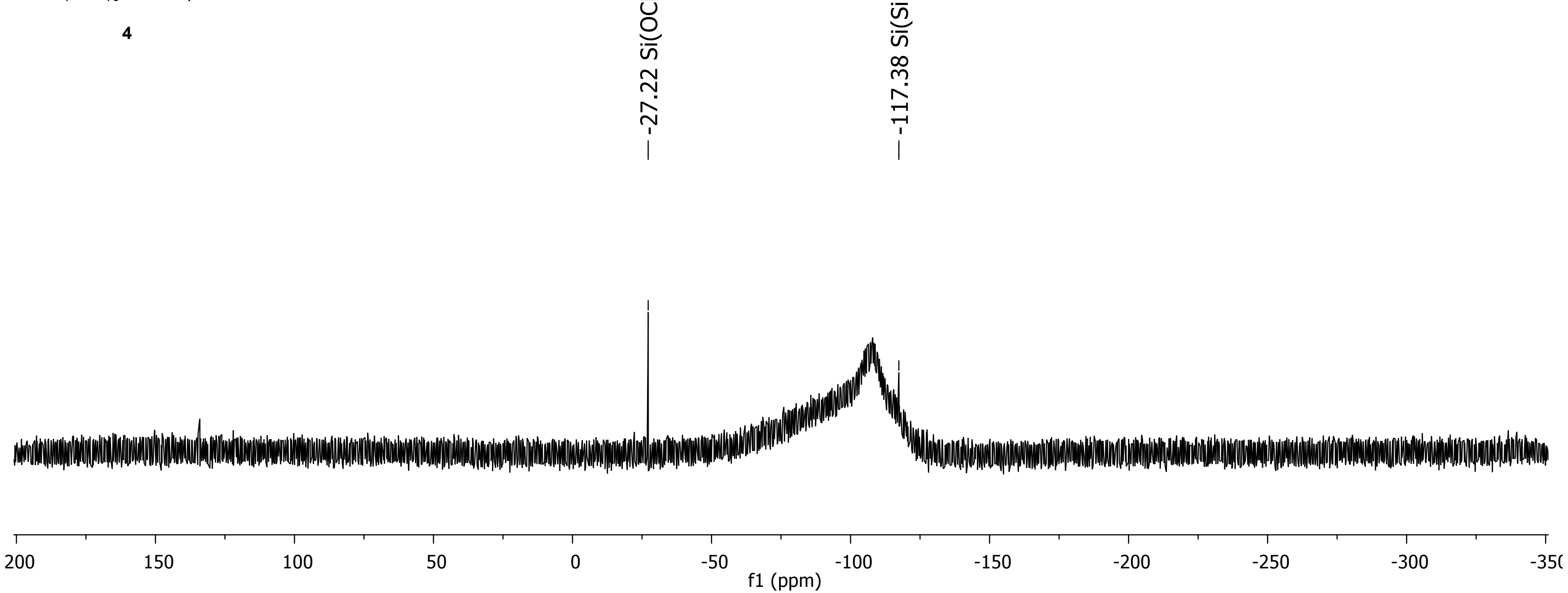

Figure S9: ^{13}C-NMR spectra of (η^5-dicyclopentadienyl)(1,1,1,3,3,3-hexamethoxy-2-(trimethoxyysilyl)trisilan-2-yl)hafnium(IV) chloride (**4**) (C_6D_6 solution, 75.43 MHz, RT, ppm)

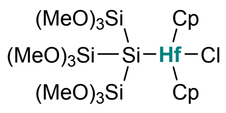

4

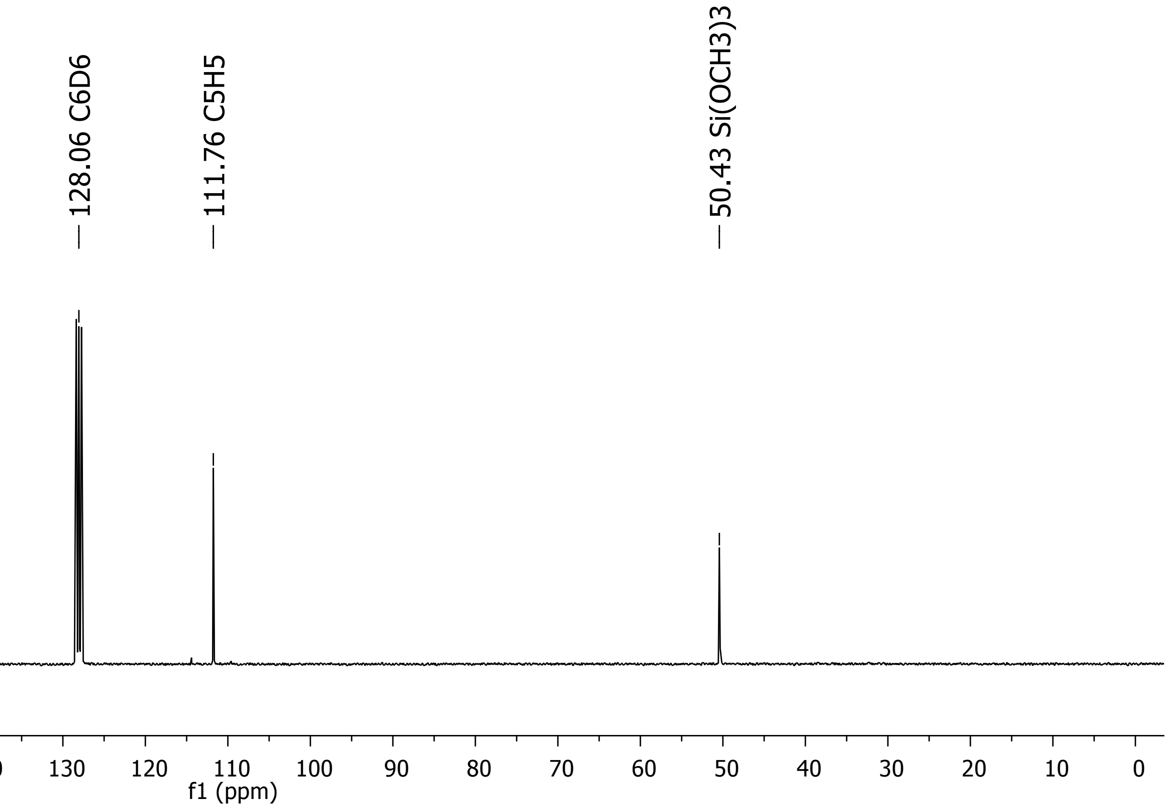

Figure S10: ^{1}H-NMR spectra of (η^{5}-dicyclopentadienyl)(1,1,1,3,3,3-hexamethoxy-2-(trimethoxysilyl)trisilan-2-yl)((2,2,6,6-tetramethylpiperidin-1-yl)oxy)titanium (**6**) (C_6D_6 solution, 299.95 MHz, RT, ppm)

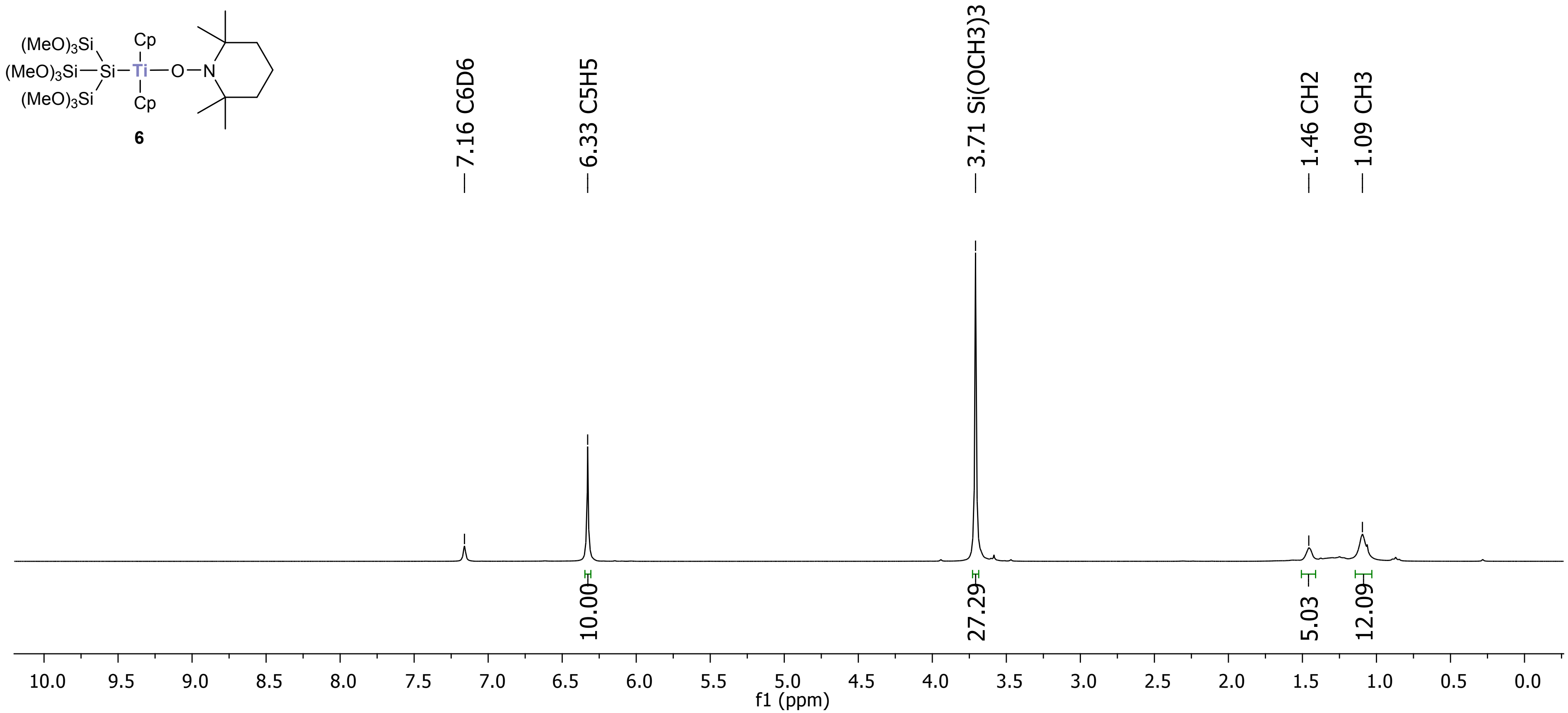

Figure S11: ^{29}Si-NMR spectra of (η^5-dicyclopentadienyl)(1,1,1,3,3,3-hexamethoxy-2-(trimethoxysilyl)trisilan-2-yl)((2,2,6,6-tetramethylpiperidin-1-yl)oxy)titanium (**6**) (C_6D_6 solution, 39.73 MHz, RT, ppm)

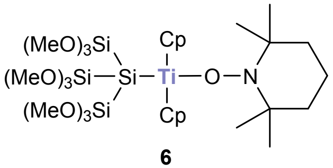

6

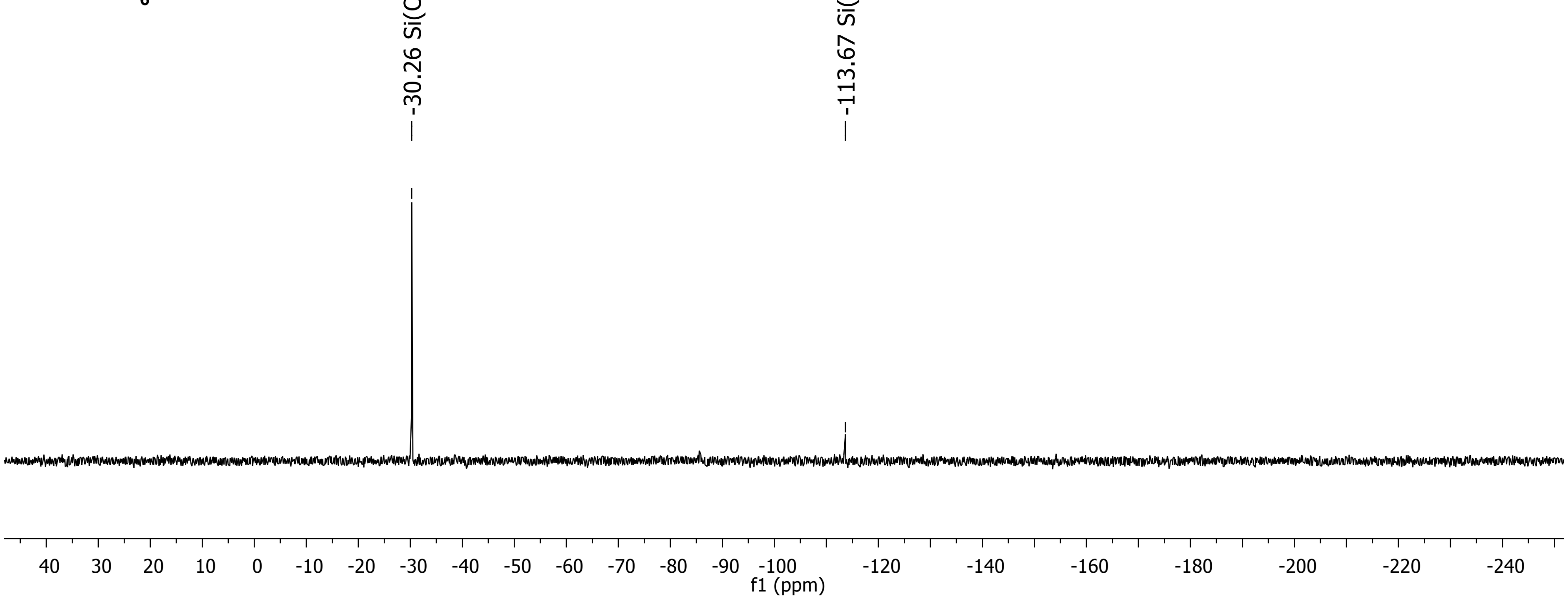

Figure S12: ^{13}C-NMR spectra of (η^5-dicyclopentadienyl)(1,1,1,3,3,3-hexamethoxy-2-(trimethoxysilyl)trisilan-2-yl)((2,2,6,6-tetramethylpiperidin-1-yl)oxy)titanium (**6**) (C_6D_6 solution, 75.43 MHz, RT, ppm)

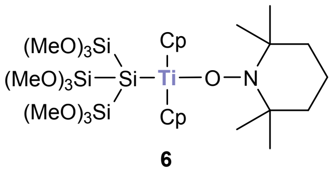

6

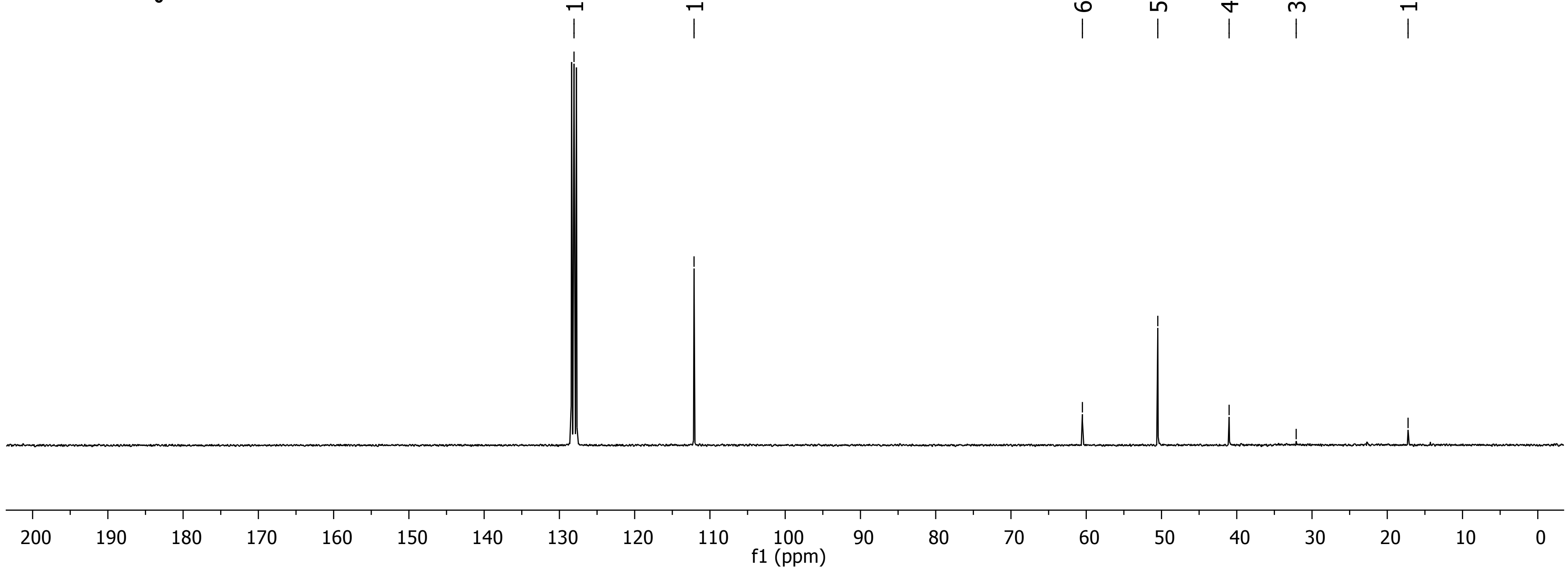

Figure S13: ^{1}H-NMR spectra of (η^5-dicyclopentadienyl)(1,1,1,3,3,3-hexamethoxy-2-(trimethoxysilyl)trisilan-2-yl)titanium(IV) bromide (**7**) (C_6D_6 solution, 299.95 MHz, RT, ppm)

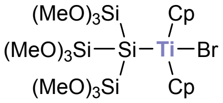

7

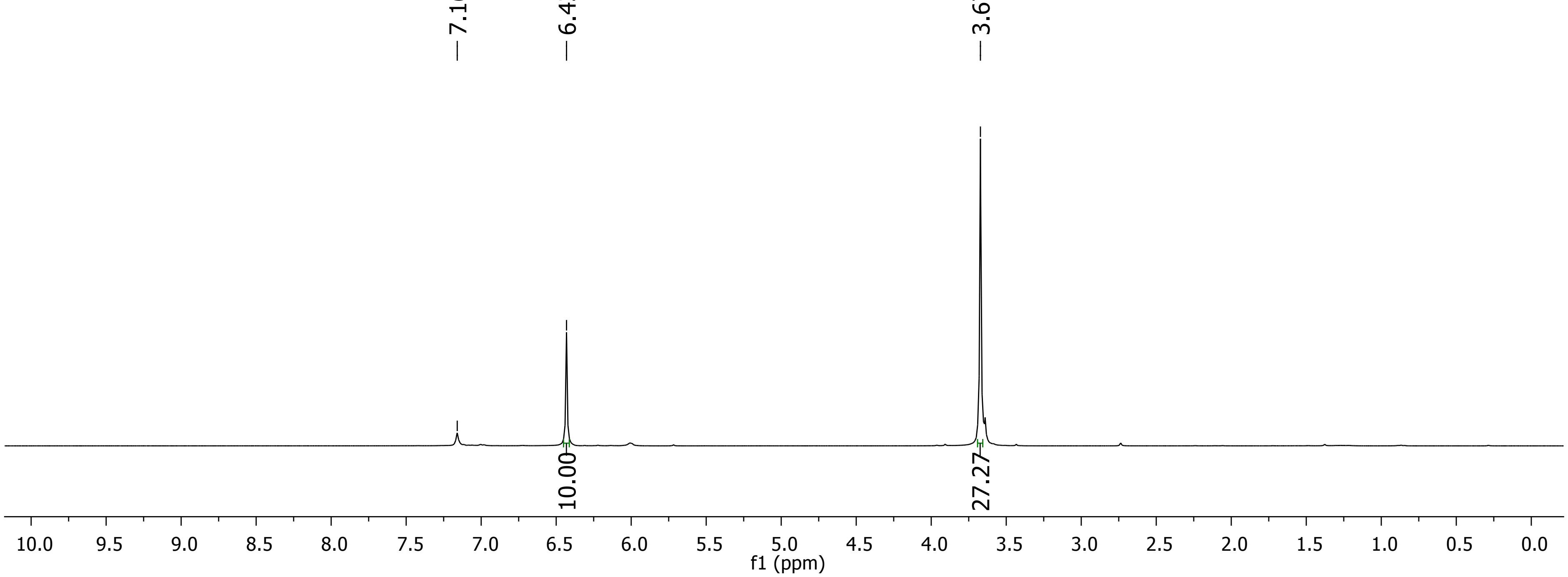

Figure S14: ^{29}Si-NMR spectra of (η^5-dicyclopentadienyl)(1,1,1,3,3,3-hexamethoxy-2-(trimethoxysilyl)trisilan-2-yl)titanium(IV) bromide (**7**) (C_6D_6 solution, 39.73 MHz, RT, ppm)

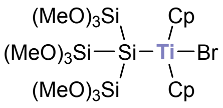

7

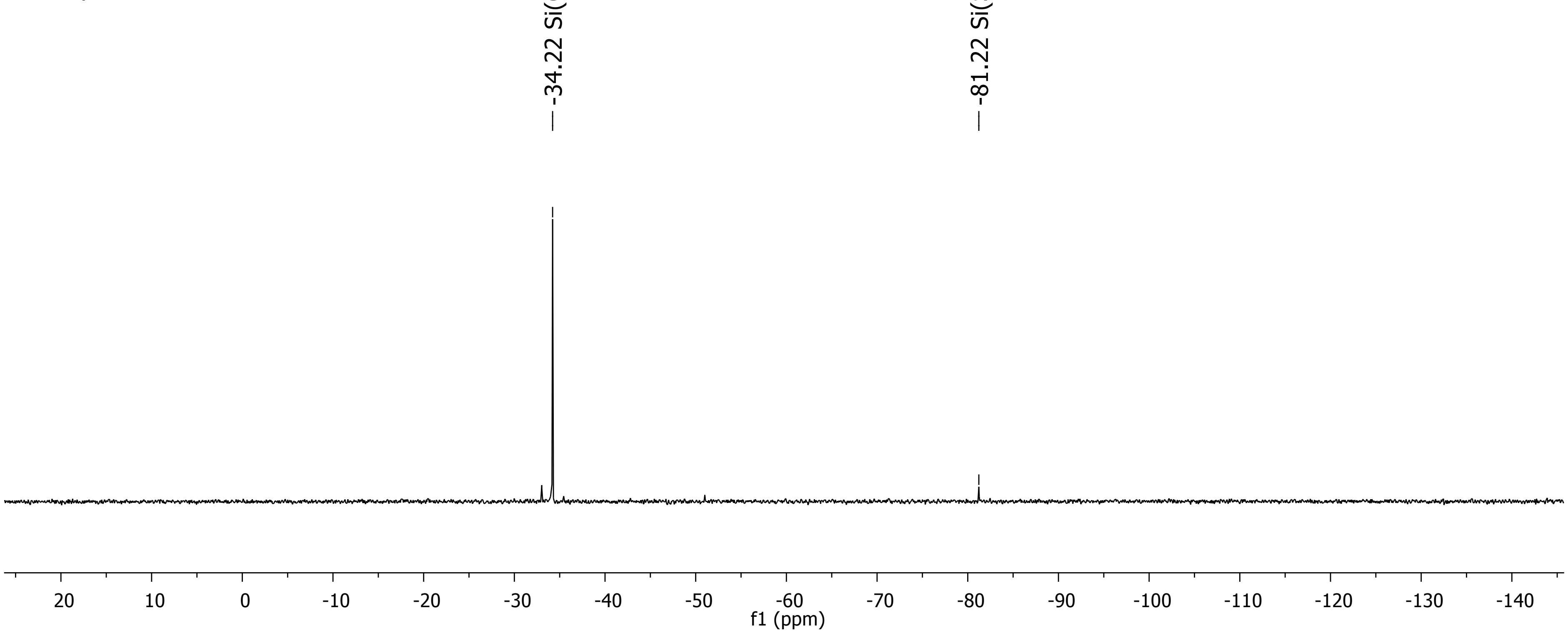

Figure S15: ^{13}C-NMR spectra of (η^5-dicyclopentadienyl)(1,1,1,3,3,3-hexamethoxy-2-(trimethoxysilyl)trisilan-2-yl)titanium(IV) bromide (**7**) (C_6D_6 solution, 75.43 MHz, RT, ppm)

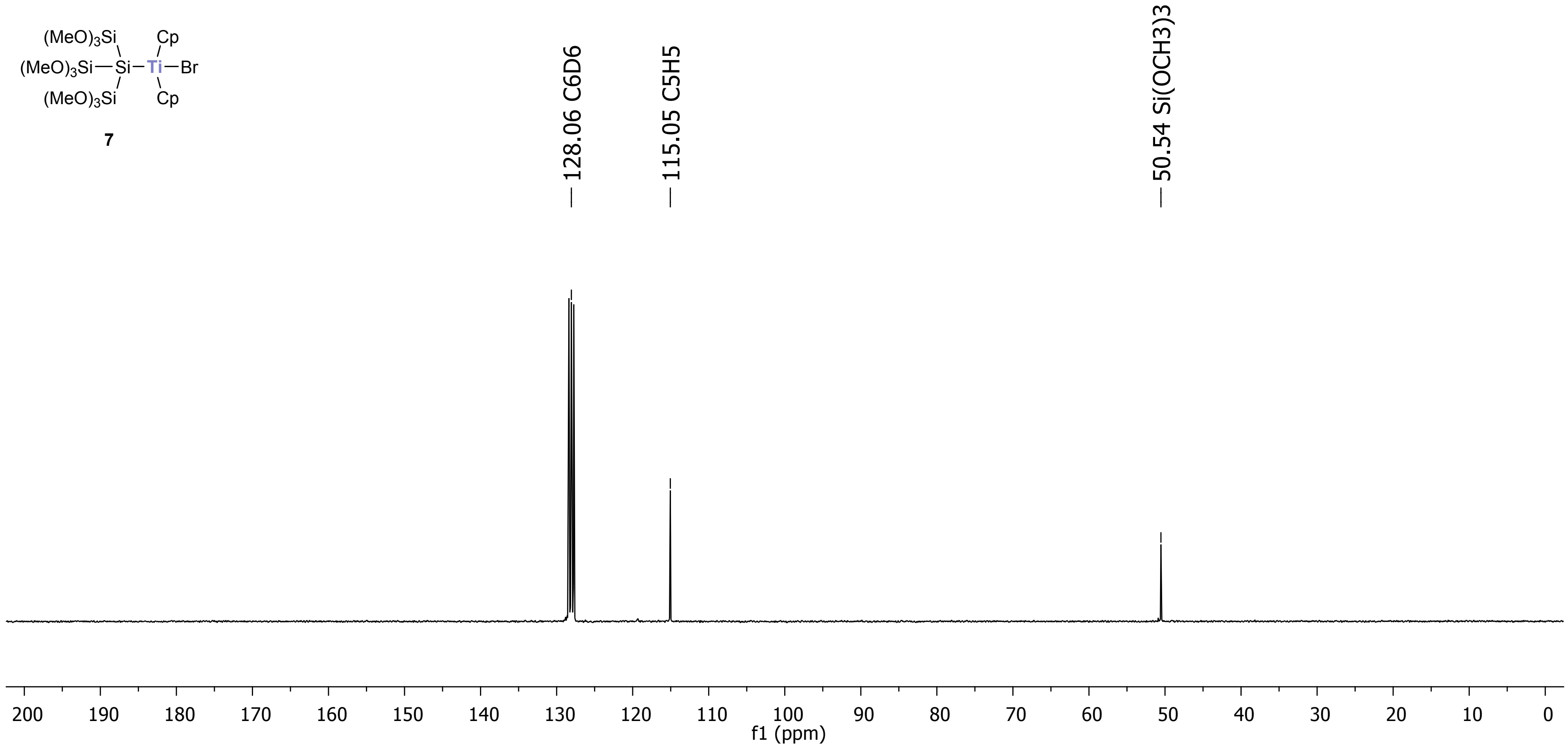

Figure S16: ^{1}H-spectra of 2,2,3,3-tetramethyl-1,1,4,4-tetra(trimethoxysilyl)-1,4-tetradisilanide (**8**) (C_6D_6 solution, 199.97 MHz, RT, ppm)

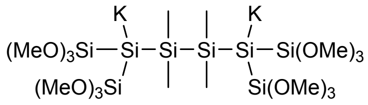

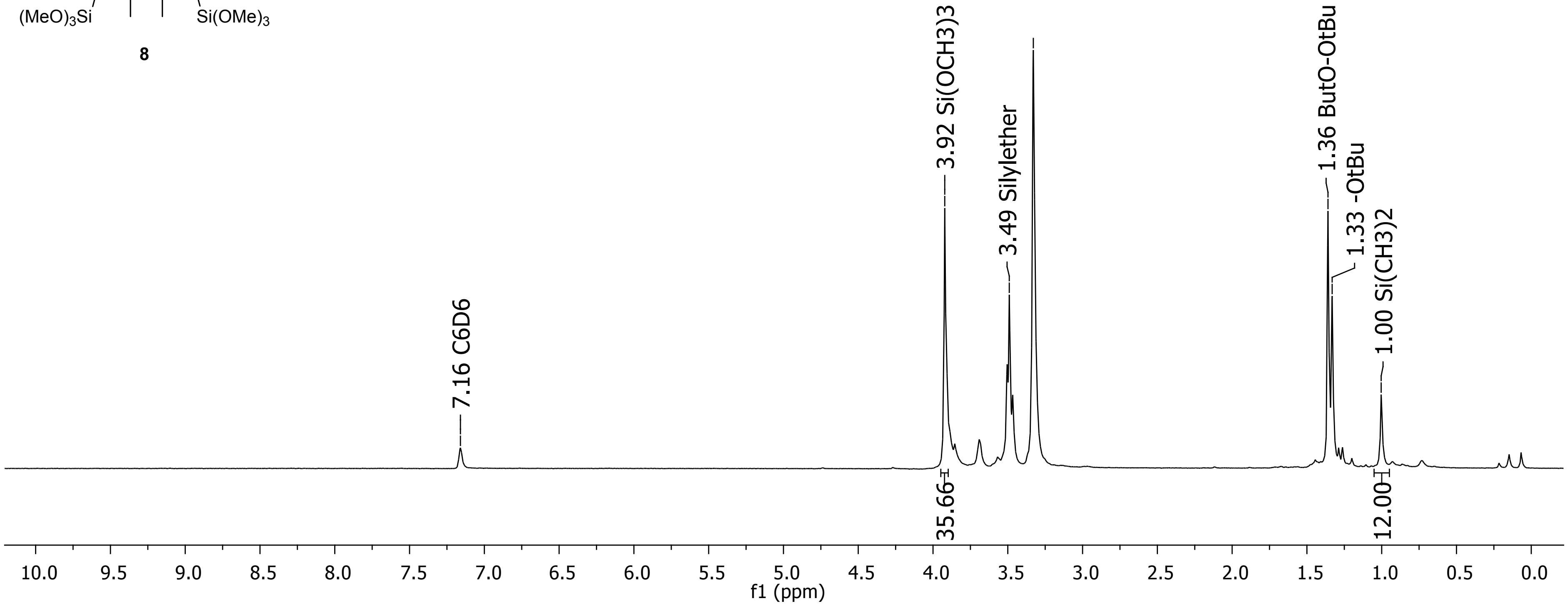

Figure S17: ^{29}Si-NMR spectra of 2,2,3,3-tetramethyl-1,1,4,4-tetra(trimethoxysilyl)-1,4-tetradisilanide (**8**) (C_6D_6 solution, 39.73 MHz, RT, ppm)

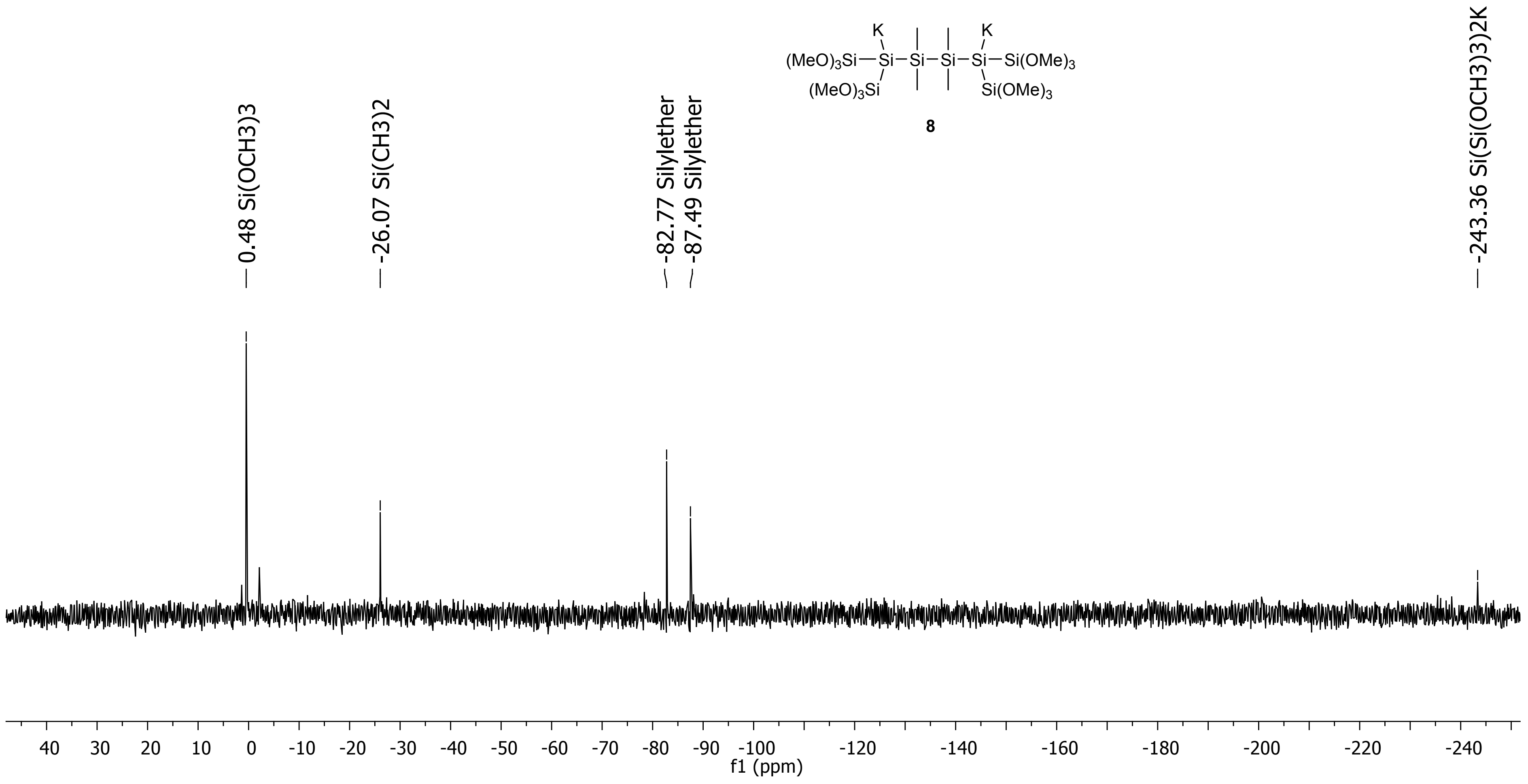

Figure S18: ^{1}H-NMR spectra of (η^{5}-dicyclopentadienyl)-2,2,5,5-tetrakis (trimethoxysilyl)tetramethoxy-1-titanacyclopentasilane (**9**) (C_6D_6 solution, 299.95 MHz, RT, ppm)

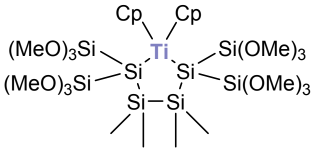

9

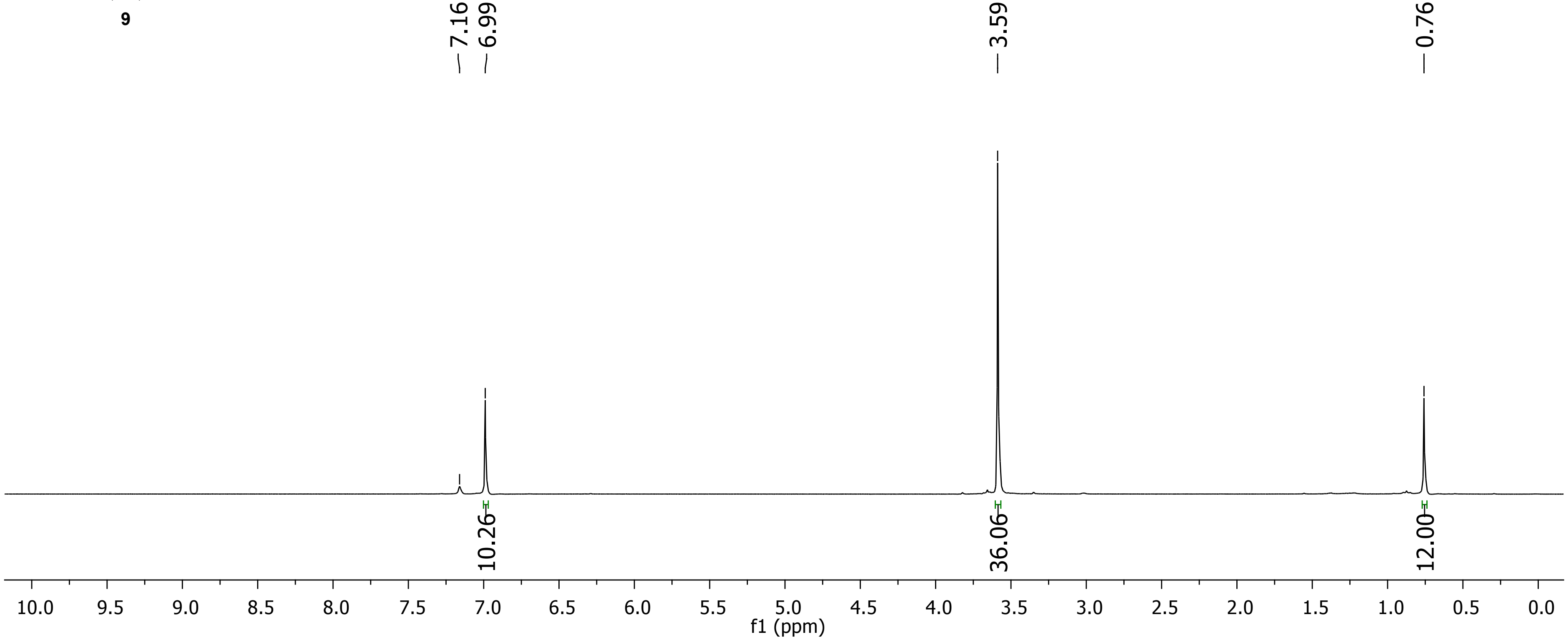

Figure S19: ^{29}Si-NMR spectra of (η^5-dicyclopentadienyl)-2,2,5,5-tetrakis (trimethoxysilyl)tetramethoxy-1-titanacyclopentasilane (**9**) (C_6D_6 solution, 39.73 MHz, RT, ppm)

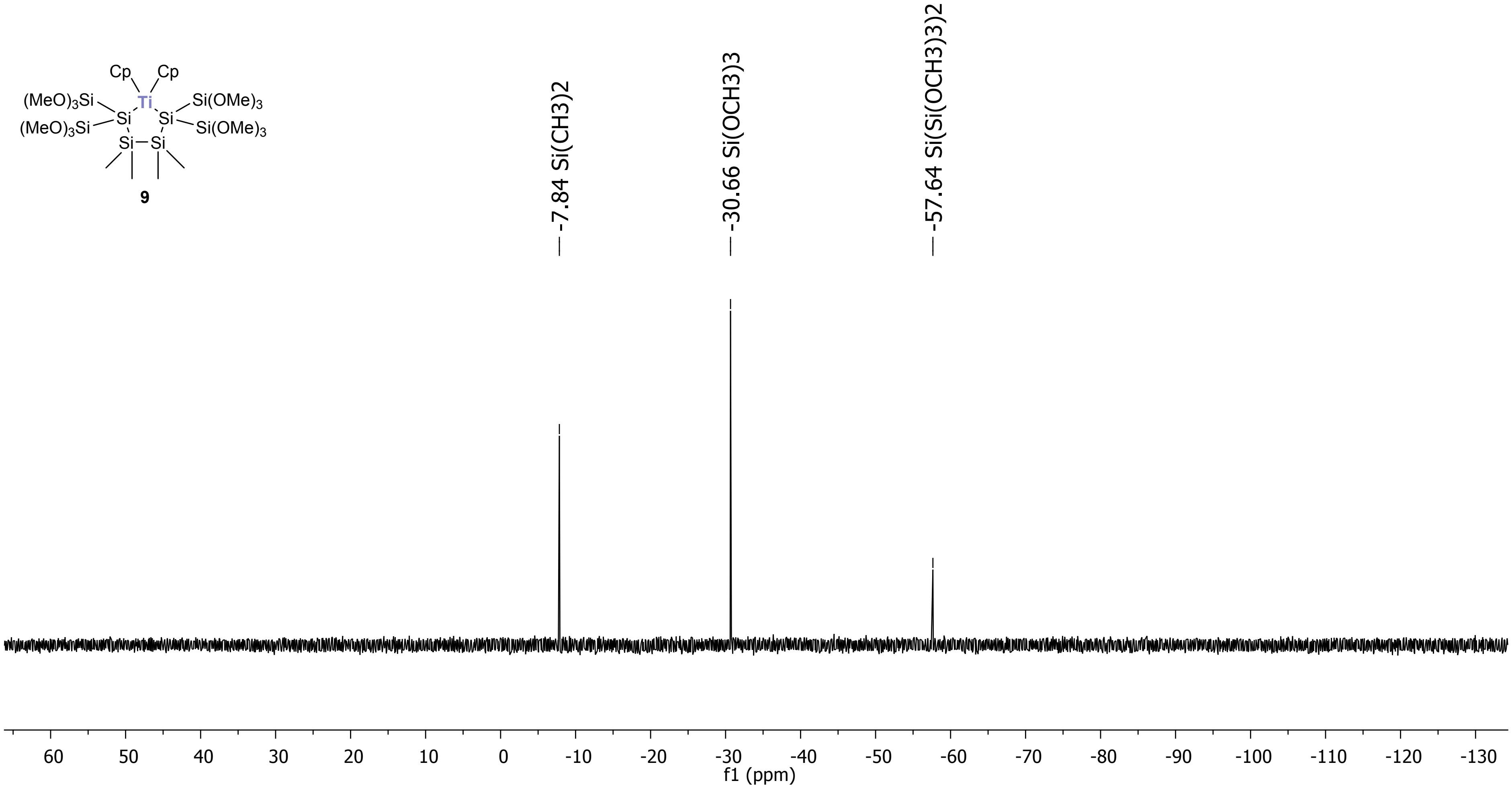

Figure S20: ^{13}C-NMR spectra of (η^5-dicyclopentadienyl)-2,2,5,5-tetrakis (trimethoxysilyl)tetramethoxy-1-titanacyclopentasilane (**9**) (C_6D_6 solution, , 75.43 MHz, RT, ppm)

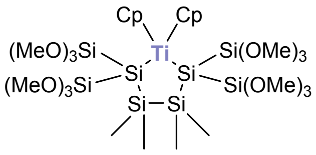

9

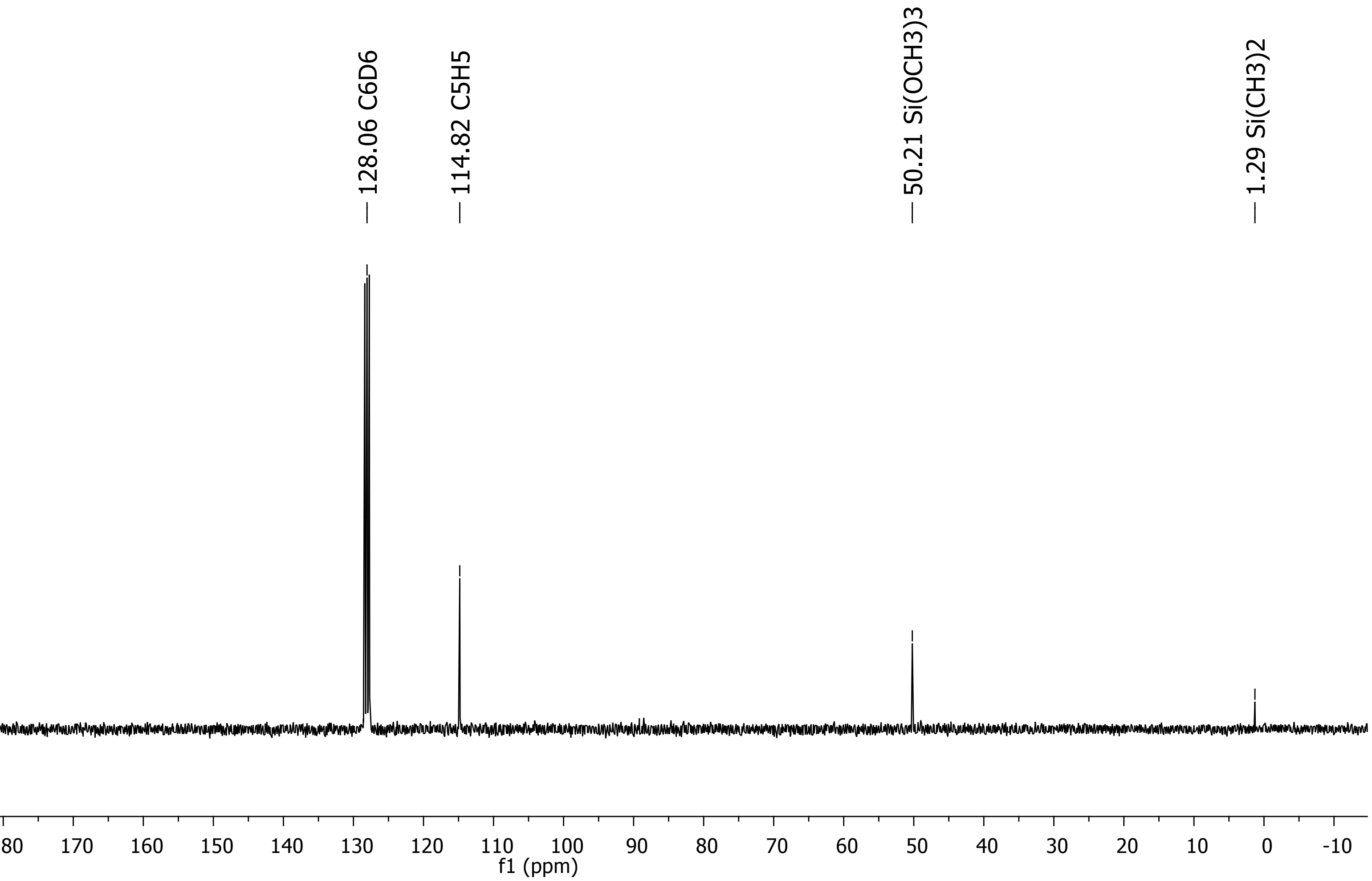

Figure S21: ^{1}H-NMR spectra of (η[5]-dicyclopentadienyl)-2,2,5,5-tetrakis (trimethoxysilyl)tetramethoxy-1-zirconacyclopentasilane (**10**) (C_6D_6 solution, 299.95 MHz, RT, ppm)

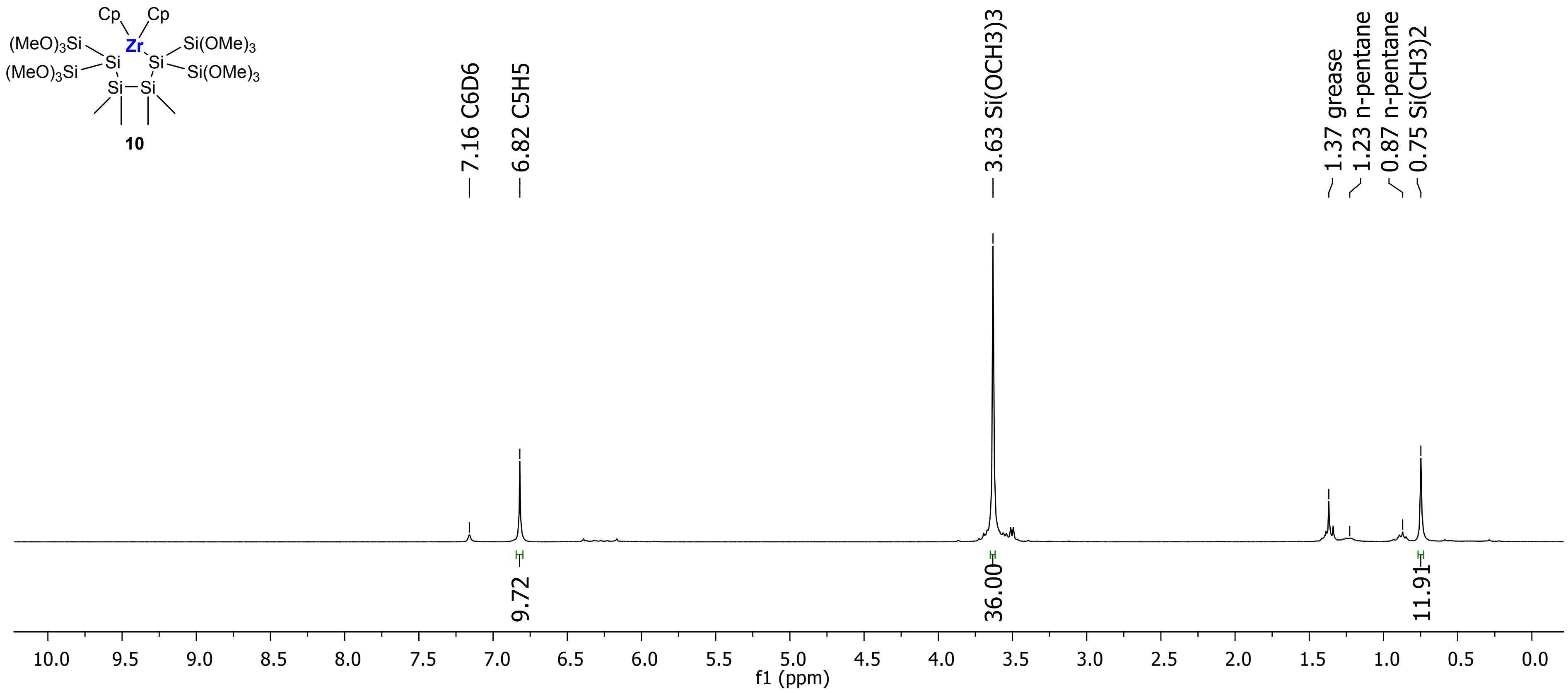

Figure S22: ^{29}Si-NMR spectra of (η^5-dicyclopentadienyl)-2,2,5,5-tetrakis (trimethoxysilyl)tetramethoxy-1-zirconacyclopentasilane (**10**) (C_6D_6 solution, 39.73 MHz, RT, ppm)

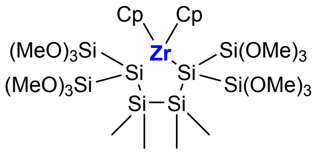

10

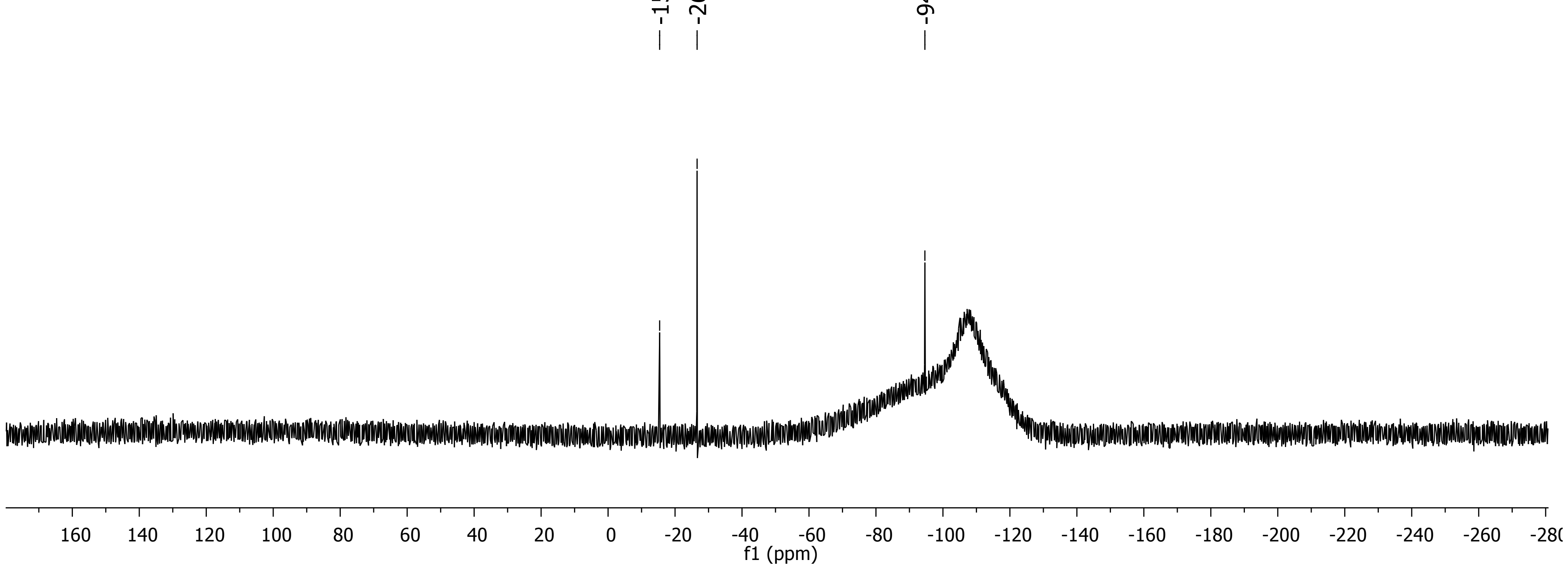

Figure S23: ^{13}C-NMR spectra of (η^5-dicyclopentadienyl)-2,2,5,5-tetrakis (trimethoxysilyl)tetramethoxy-1-zirconacyclopentasilane (**10**) (C_6D_6 solution, 75.43 MHz, RT, ppm)

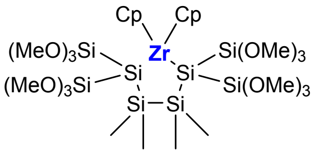

10

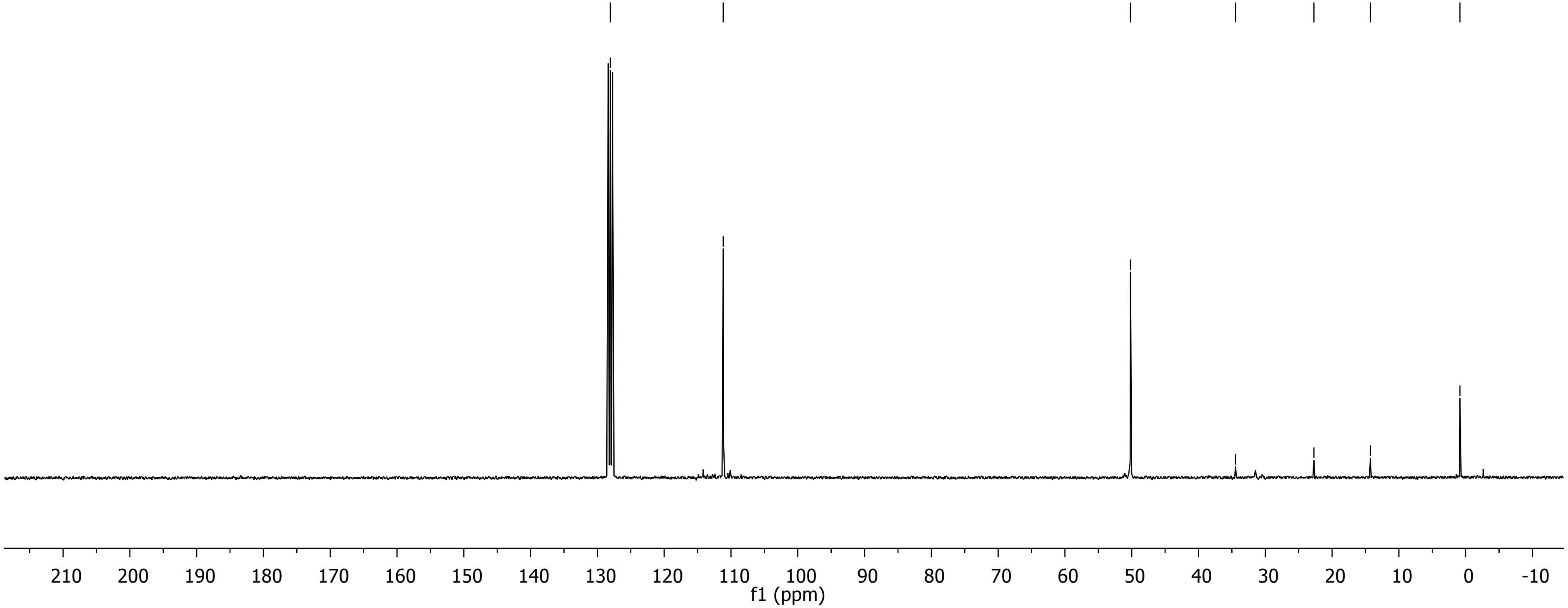

Figure S24: ^{1}H-NMR spectra of (η[5]-dicyclopentadienyl)-2,2,5,5-tetrakis (trimethoxysilyl)tetramethoxy-1-hafnacyclopentasilane (C_6D_6 solution, 299.95 MHz, RT, ppm)

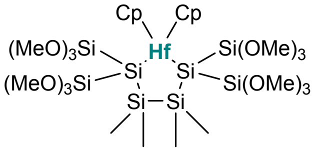

11

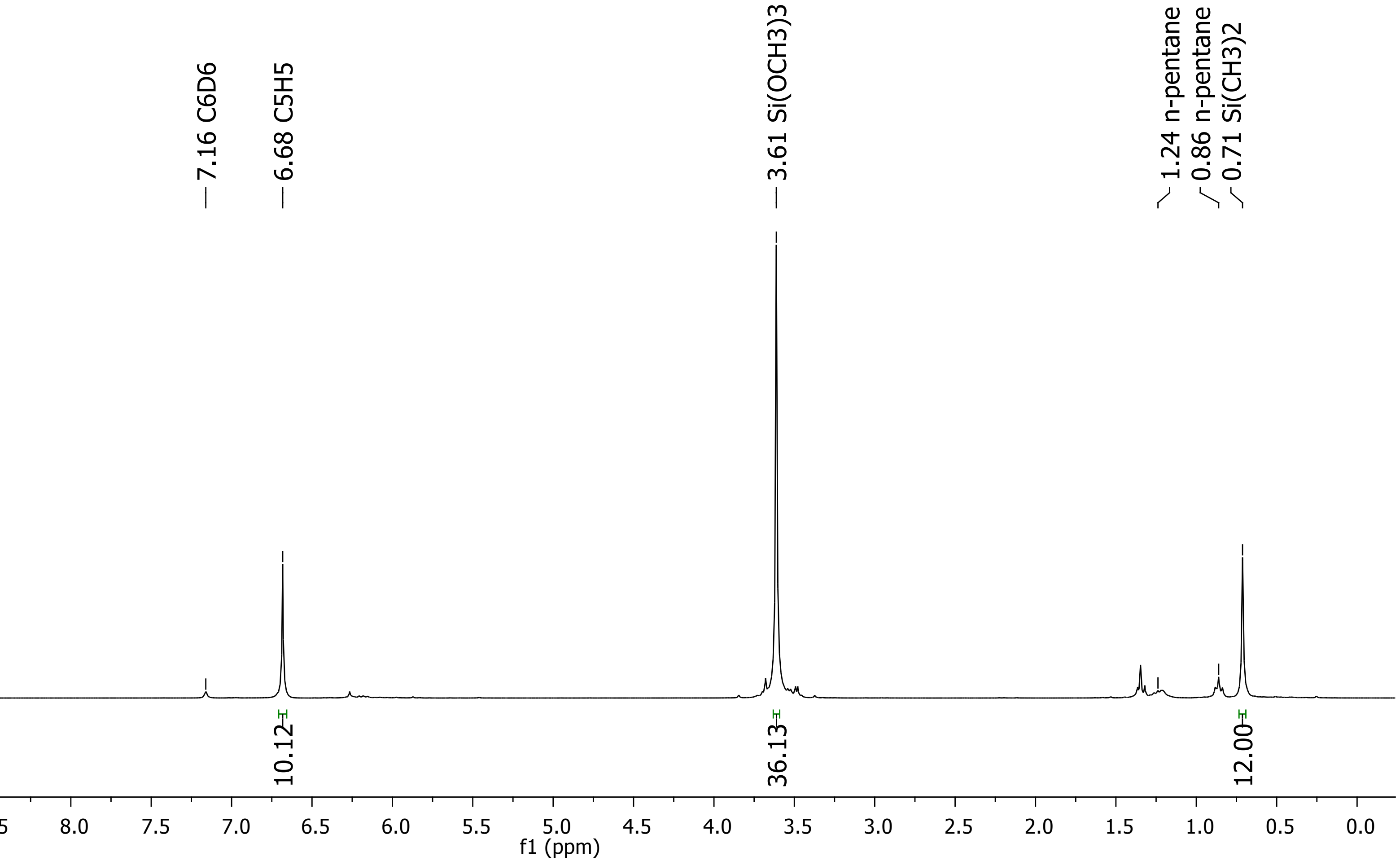

Figure S25: ^{29}Si-NMR spectra of (η^5-dicyclopentadienyl)-2,2,5,5-tetrakis (trimethoxysilyl)tetramethoxy-1-hafnacyclopentasilane (C_6D_6 solution, 39.73 MHz, RT, ppm)

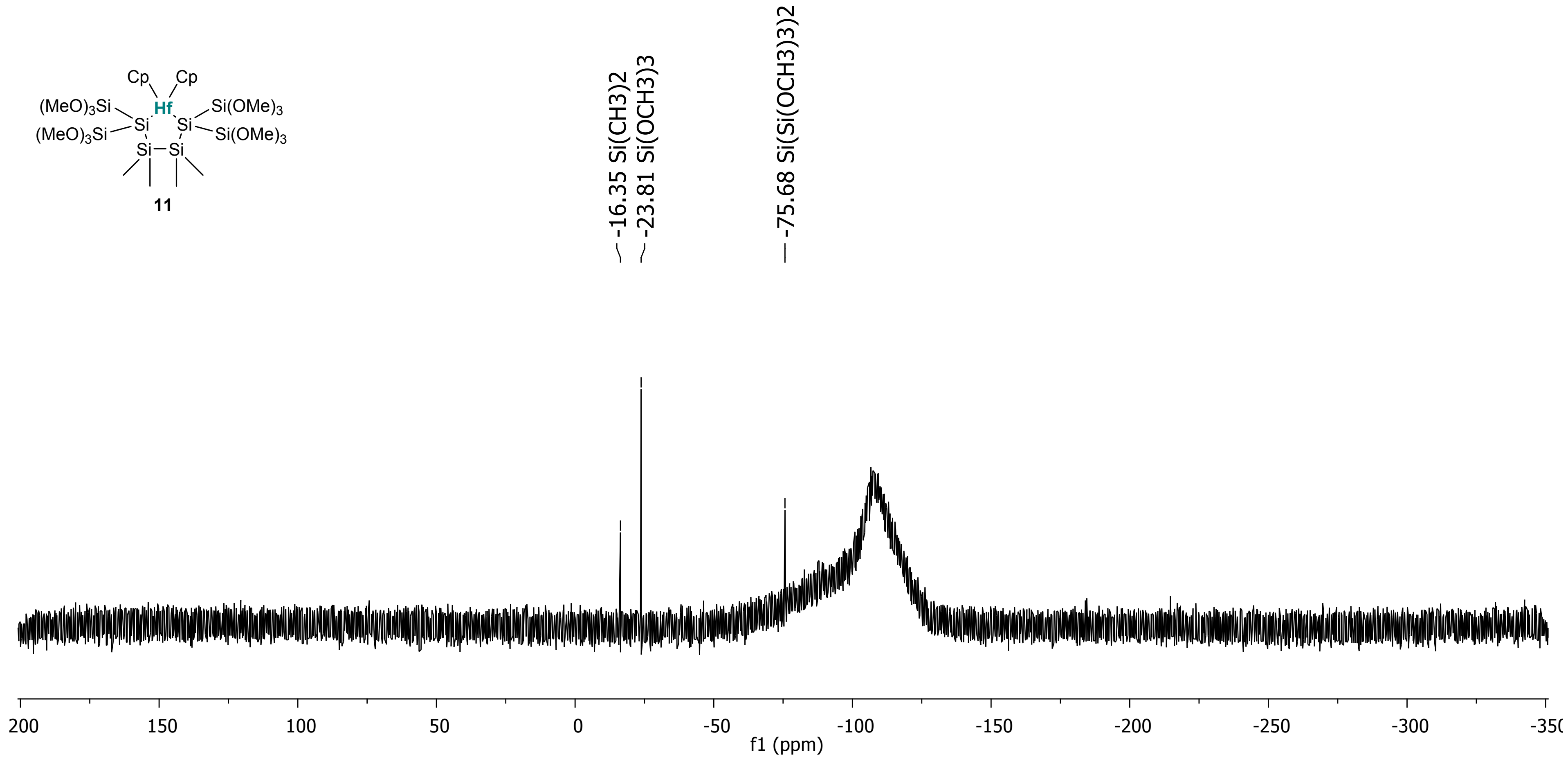

Figure S26: ^{13}C-NMR spectra of (η^5-dicyclopentadienyl)-2,2,5,5-tetrakis (trimethoxysilyl)tetramethoxy-1-hafnacyclopentasilane (C_6D_6 solution, 75.43 MHz, RT, ppm)

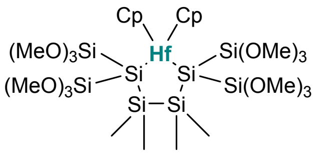

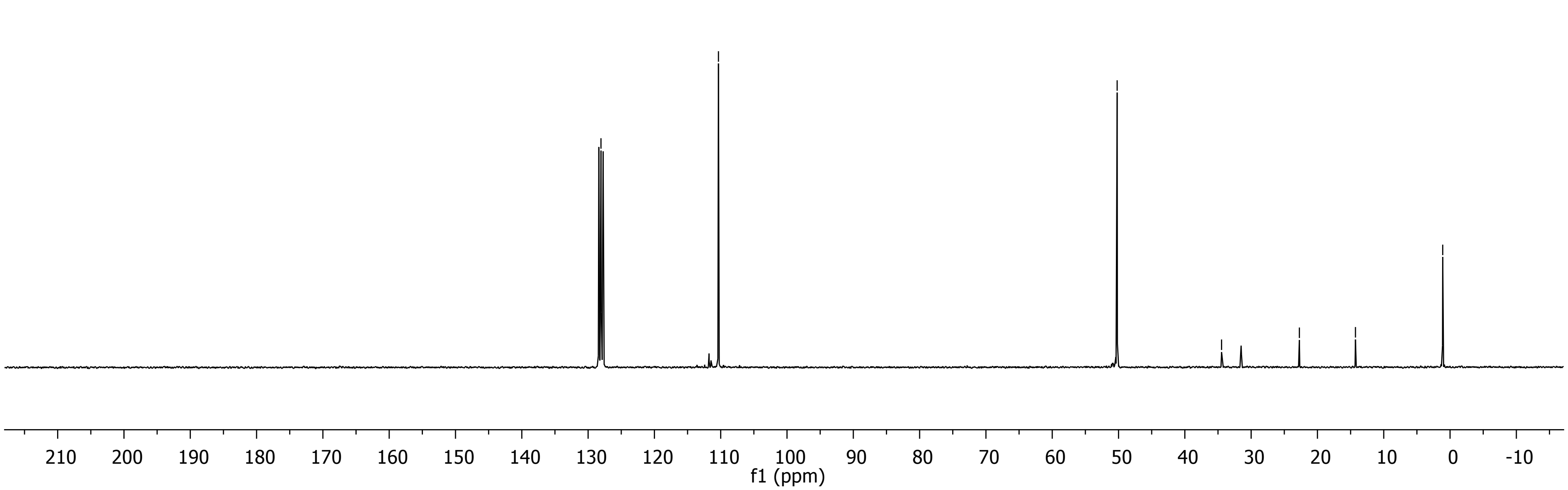

UV-Vis-Spectroscopy

Figure S27: UV-Vis spectra of compound **5** (c = $1*10^{-4}$ mol/L; solvent: *n*-hexane)

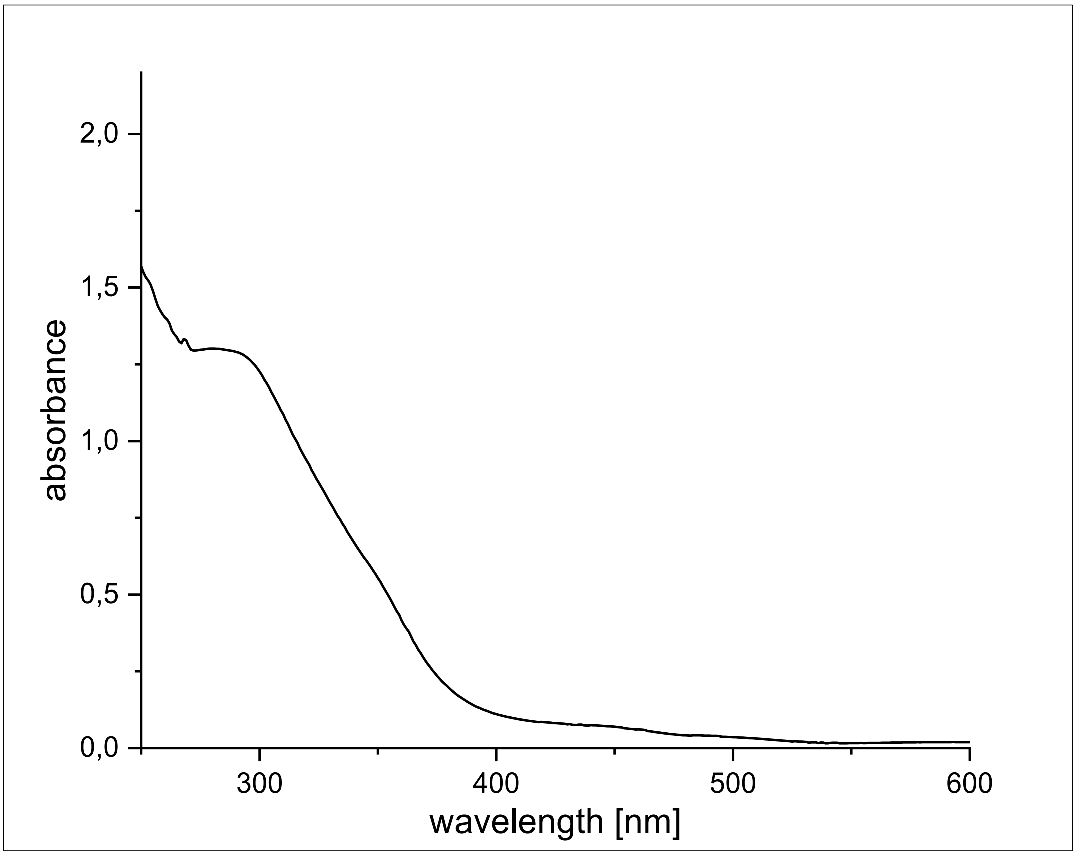

Figure S28 UV-Vis spectra of compound **6** (c = 1*10^{-4} mol/L; solvent: *n*-hexane)

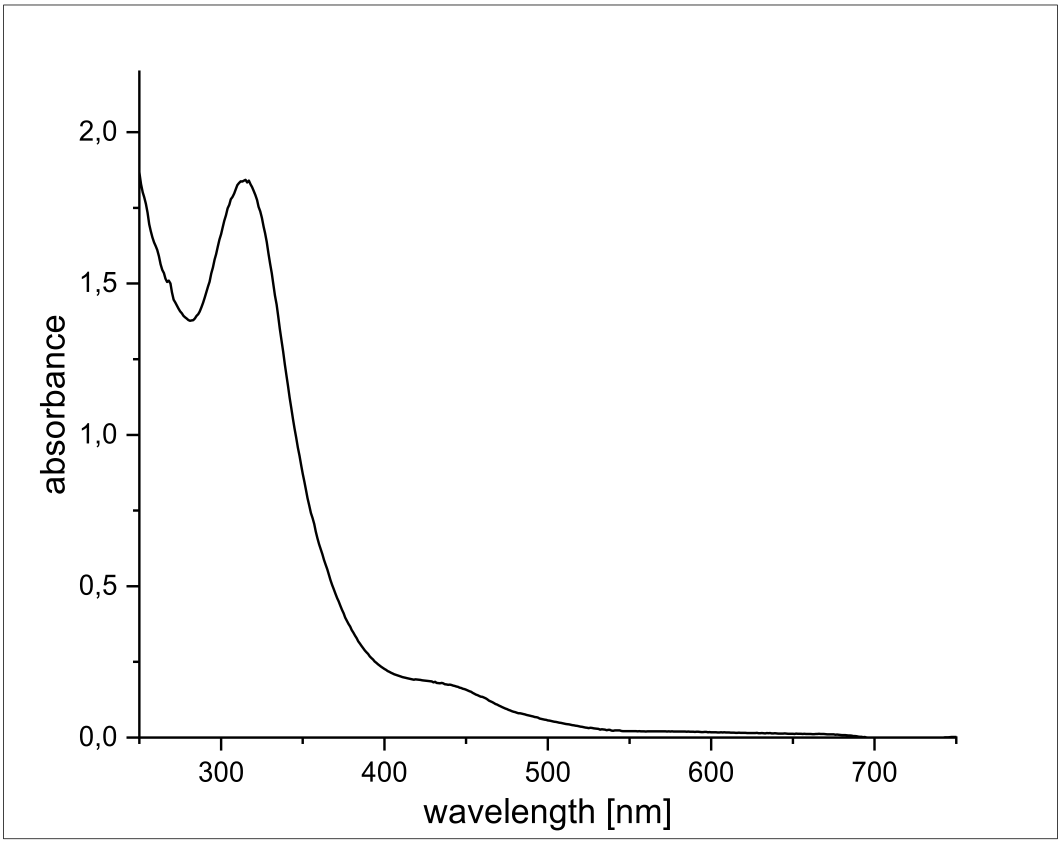

Figure S29: UV-Vis spectra of compounds **7** (c = 1*10^{-4} mol/L; solvent: *n*-hexane)

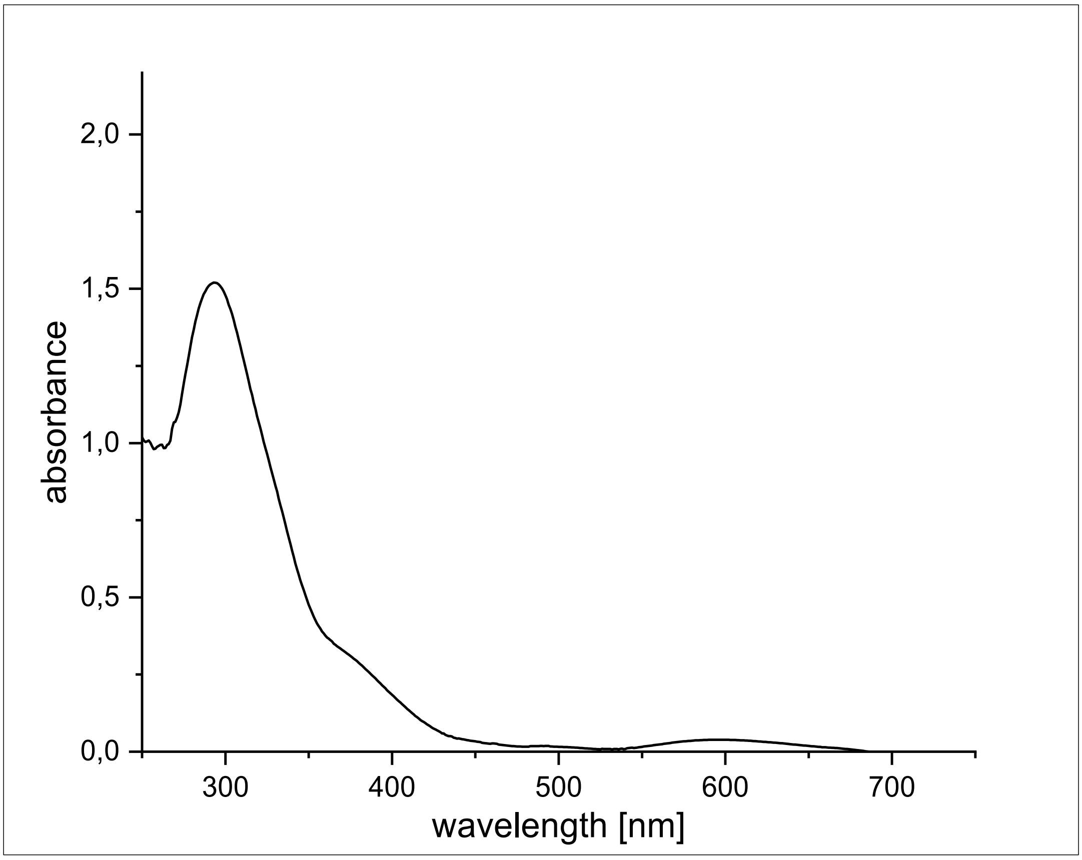

Crystal Structures

Figure S30: ORTEP for compound **3**.

Thermal ellipsoids are depicted at the 50% probability level. Hydrogen atoms are omitted and carbon atoms are wireframed for clarity. Selected bond lengths (Å) and bond angles (deg) with estimated standard deviations: Zr(1)-Cl(1) 2.4310(7), Zr(1)-Si(1) 2.8118(7), Si(1)-Si(2) 2.3407(10), Si(1)-Si(3) 2.3383(10), Si(1)-Si(4) 2.3448(10), Cl(1)-Zr(1)-Si(1) 98.91(2), Si(2)-Si(1)-Zr(1) 107.79(3), Si(4)-Si(1)-Zr(1) 117.69(3), Si(3)-Si(1)-Zr(1) 114.54(3), Si(2)-Si(1)-Si(4) 106.33(4), Si(3)-Si(1)-Si(2) 106.64(4), Si(3)-Si(1)-Si(4) 103.08(4)

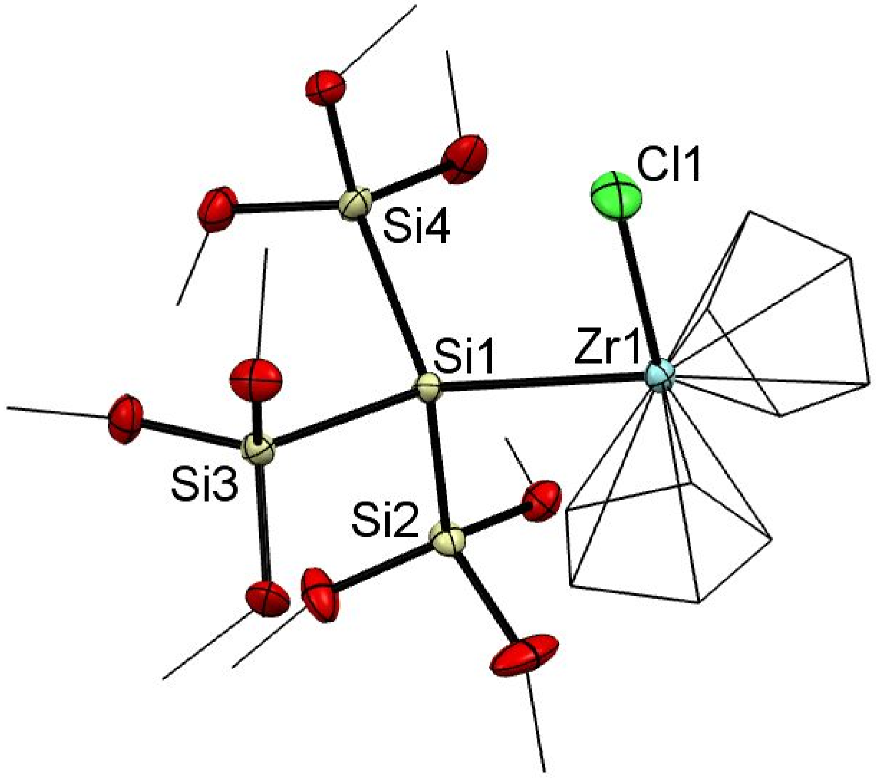

Figure S31: ORTEP for compound **4**.

Thermal ellipsoids are depicted at the 50% probability level. Hydrogen atoms are omitted and carbon atoms are wireframed for clarity. Selected bond lengths (Å) and bond angles (deg) with estimated standard deviations: Hf(1)-Cl(1) 2.4049(14), Hf(1)-Si(1) 2.7770(16), Si(1)-Si(2) 2.337(2), Si(1)-Si(3) 2.344(2), Si(1)-Si(4) 2.335(2), Cl(1)-Hf(1)-Si(1) 97.94(5), Si(4)-Si(1)-Hf(1) 115.40(7), Si(2)-Si(1)-Hf(1) 107.96(8), Si(3)-Si(1)-Hf(1) 117.78(7), Si(4)-Si(1)-Si(2) 105.77(8), Si(4)-Si(1)-Si(3) 102.84(9), Si(2)-Si(1)-Si(3) 106.13(9).

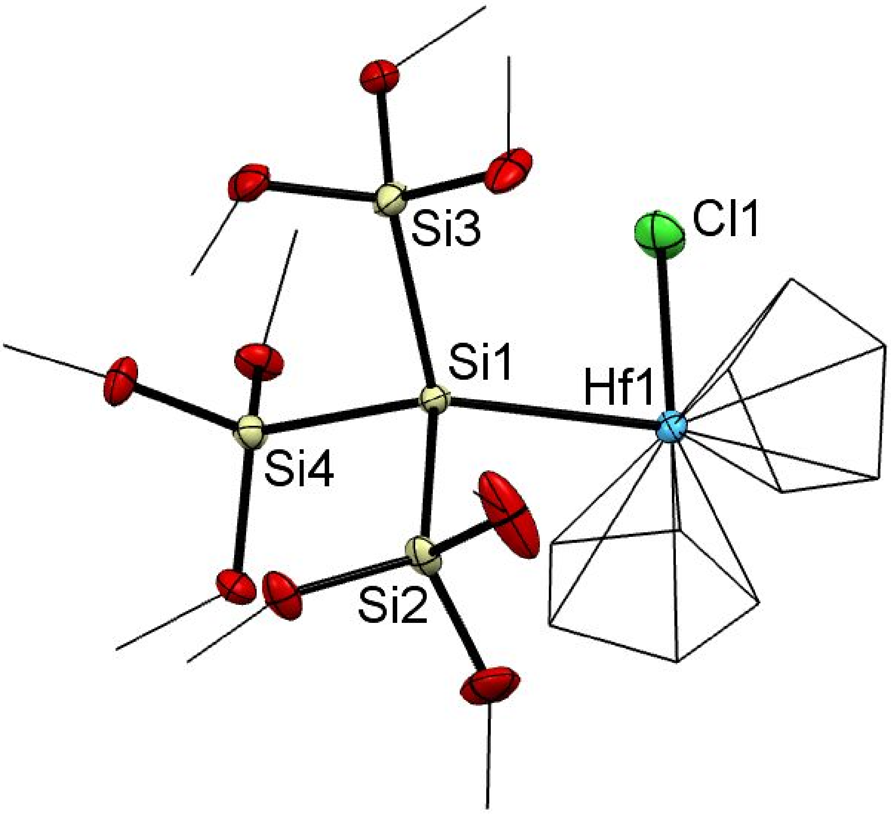

Table S1: Crystallographic data of compounds **2**, **3**, **4**, **5**, **8**, and **9**

**Compound**	**2** (2176007)	**3** (2176008)	**4** (2176009)	**5** (2176010)	**8** (2176011)	**9** (2176012)
Formula	$C_{19}H_{37}ClO_9Si_4Ti$	$C_{19}H_{37}ClO_9Si_4Zr$	$C_{19}H_{37}ClHfO_9Si_4$	$C_{19}H_{37}O_9Si_4Ti$	$C_{40}H_{96}K_2O_{24}Si_8$	$C_{26}H_{58}O_{12}Si_8Ti$
M_r (g mol^{-1})	605.19	648.51	735.78	569.74	1264.08	835.34
a (Å)	9.593 (2)	9.6643 (4)	9.6492 (2)	10.0354 (6)	11.1806 (6)	10.4723 (7)
b (Å)	15.179 (3)	15.3291 (6)	15.3020 (4)	10.1599 (6)	11.8641 (7)	24.2315 (17)
c (Å)	10.428 (2)	10.4668 (4)	10.4693 (3)	14.6335 (9)	12.8170 (7)	26.6220 (19)
α (°)	90	90	90	90.419 (3)	98.247 (3)	78.305 (4)
β (°)	113.482 (9)	113.116 (2)	113.5468(11)	95.127 (3)	101.535 (3)	78.932 (3)
γ (°)	90	90	90	114.831 (2)	104.442 (3)	81.780 (3)
V (Å3)	1392.7(5)	1426.11 (10)	1417.10 (6)	1347.00 (14)	1579.33 (16)	6454.1 (8)
Z	2	2	2	2	1	6
Crystal size (mm)	0.33 × 0.17 × 0.14	0.12 × 0.11 × 0.08	0.19 × 0.10 × 0.07	0.17 × 0.14 × 0.06	0.24 × 0.17 × 0.11	0.39 × 0.17 × 0.07
Crystal system	Monoclinic	Monoclinic	Monoclinic	Triclinic	Triclinic	Triclinic
Space group	$P2_1$	$P2_1$	$P2_1$	P-1	P-1	P-1
d_{calc} (Mg m^{-3})	1.443	1.510	1.724	1.405	1.329	1.290
μ (mm^{-1})	0.619	0.690	3.987	0.540	0.372	0.471
T (K)	100.01	100.04	100.07	99.99	99.94	100.03
2θ range (°)	4.258 to 60.112	4.232 to 60.214	4.244 to 60.068	4.422 to 60.39	3.312 to 51.998	4.034 to 56
F (000)	636.0	672.0	736.0	602.0	678.0	2664.0
R_{int}	0.0425	0.0505	0.0551	0.1000	0.1134	0.0869
No. of measured, and independent [I > 2σ(I)] reflections	83599, 8134	58055, 8242	18000, 7866	37738, 7847	12355, 6196	456949, 31104
No. of parameters, restraints	336, 1	336, 1	316, 1	327, 36	382, 12	1433, 354
Δ›max, Δ›min (e Å^{-3})	0.28/-0.26	0.81/-0.43	2.19/-2.11	0.77/-0.92	0.44/-0.41	1.84/-1.28
R1, wR2 (all data)	R1 = 0.0281 wR2 = 0.0589	R1 = 0.0311 wR2 = 0.0601	R1 = 0.0368 wR2 = 0.0663	R1 = 0.0829 wR2 = 0.1460	R1 = 0.1319, wR2 = 0.1133	R1 = 0.0698 wR2 = 0.1420
R1, wR2 (>2σ)	R1 = 0.0254 wR2 =0.0578	R1 = 0.0281 wR2 =0.0591	R1 = 0.0329 wR2 =0.0647	R1 = 0.0587 wR2 =0.1315	R1 = 0.0628 wR2 =0.0993	R1 = 0.0501 wR2 =0.1287

Density Functional Theory Computations

Figure S32: Calculated absorption spectrum for compound **2**.

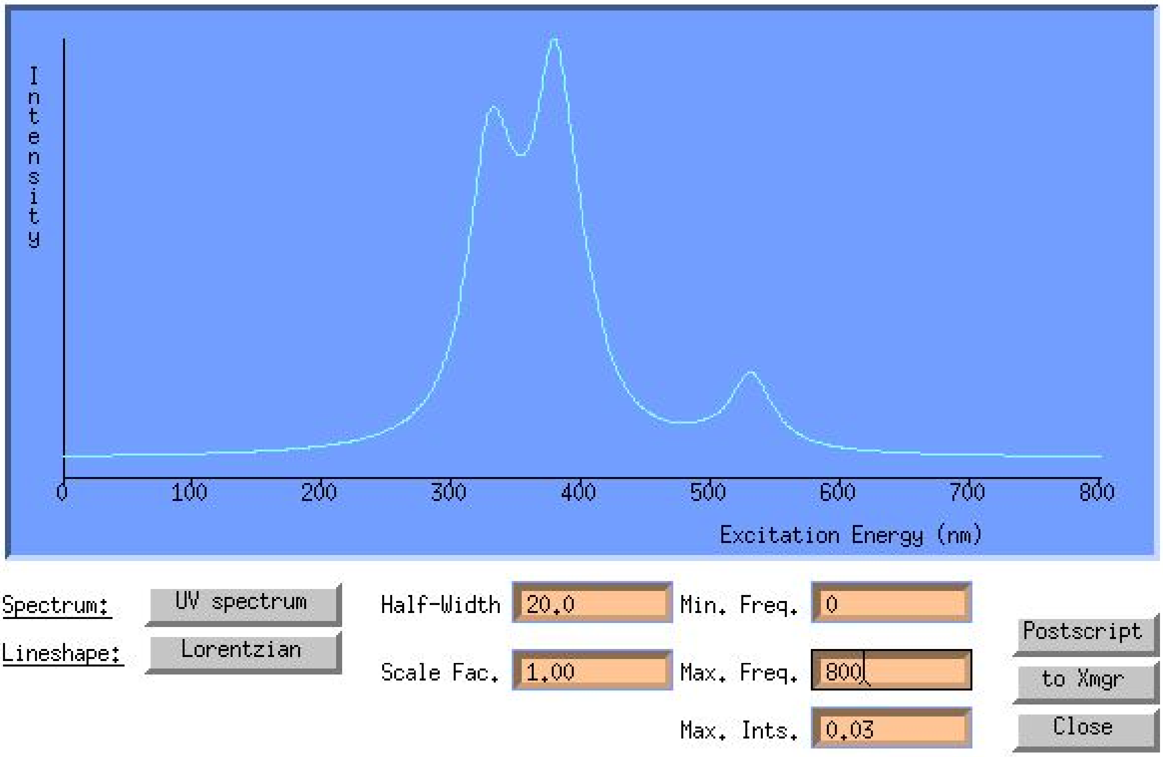

Band at λ = 332 nm, 379 nm, 531 nm

Figure S33: HOMO and LUMO for compound **3**.

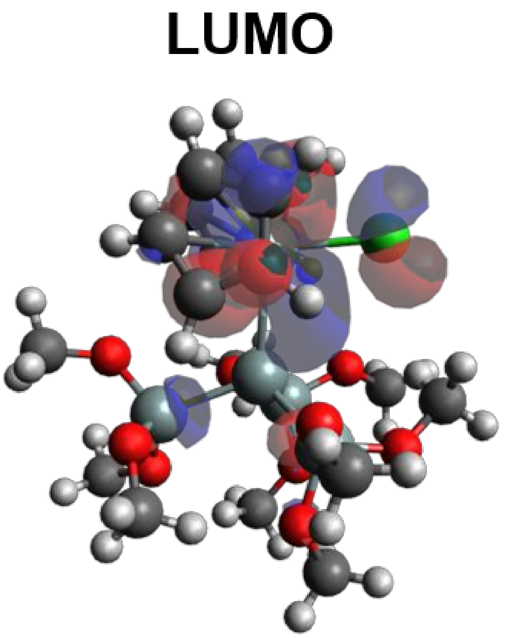

Figure S34: Calculated absorption spectrum for compound **3**.

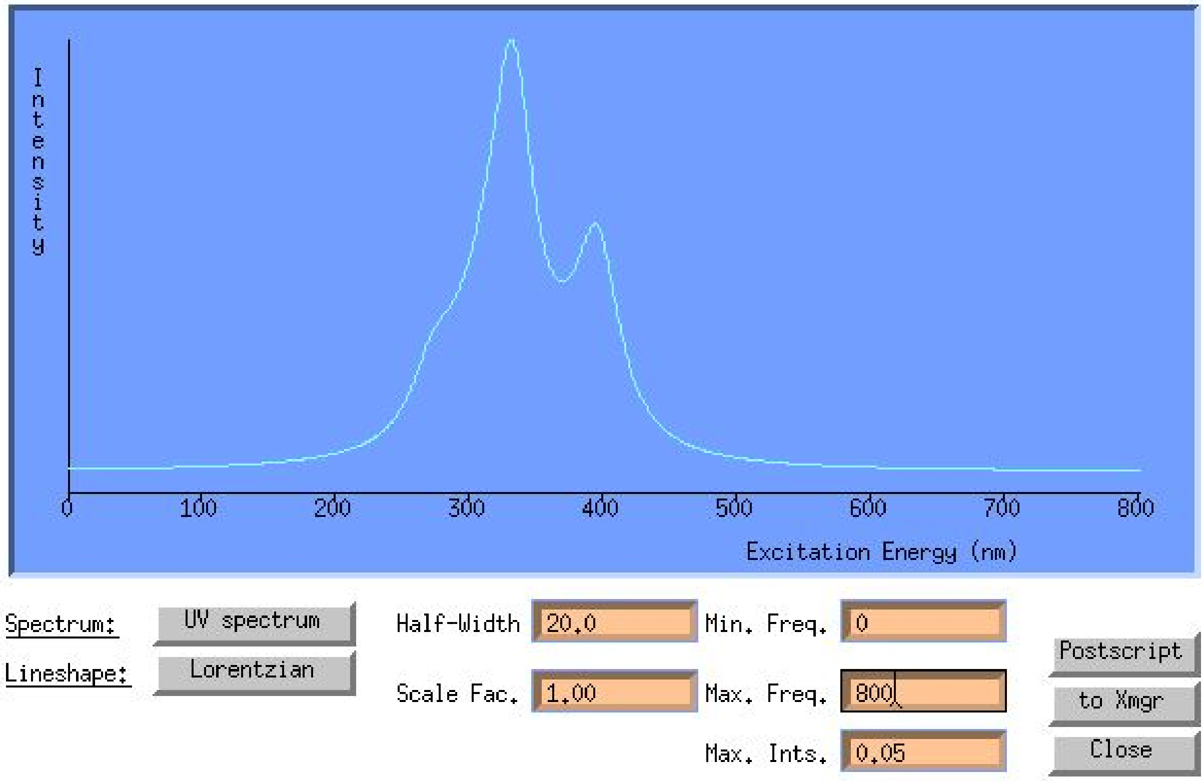

Band at λ = 273 nm, 333 nm, 395 nm

Figure S35: HOMO and LUMO for compound **4**.

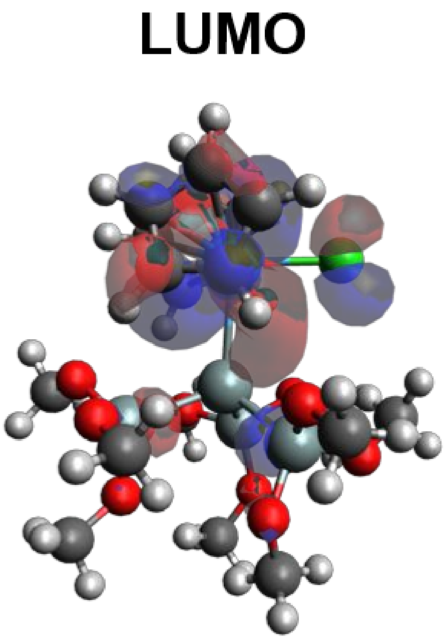

Figure S36: Calculated absorption spectrum for compound **4**.

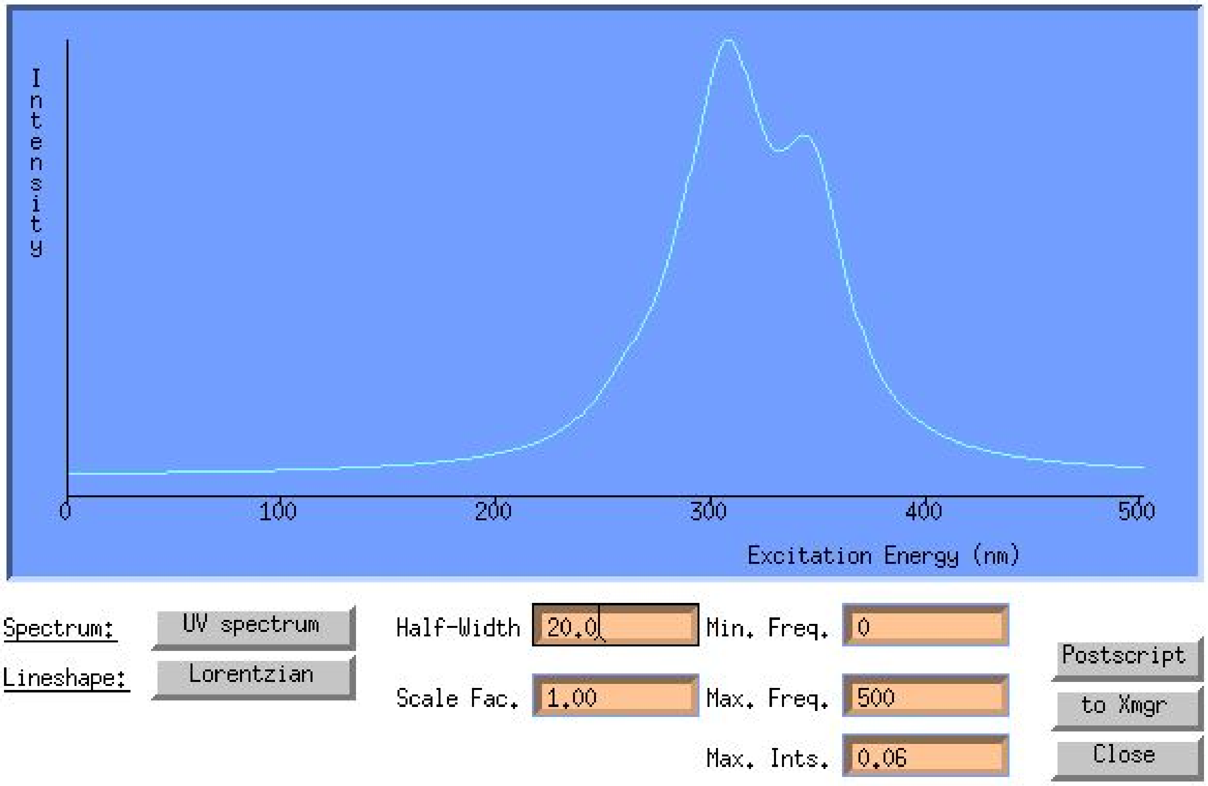

Band at λ = 310 nm, 347 nm

Figure S37 Calculated absorption spectrum for compound **9**.

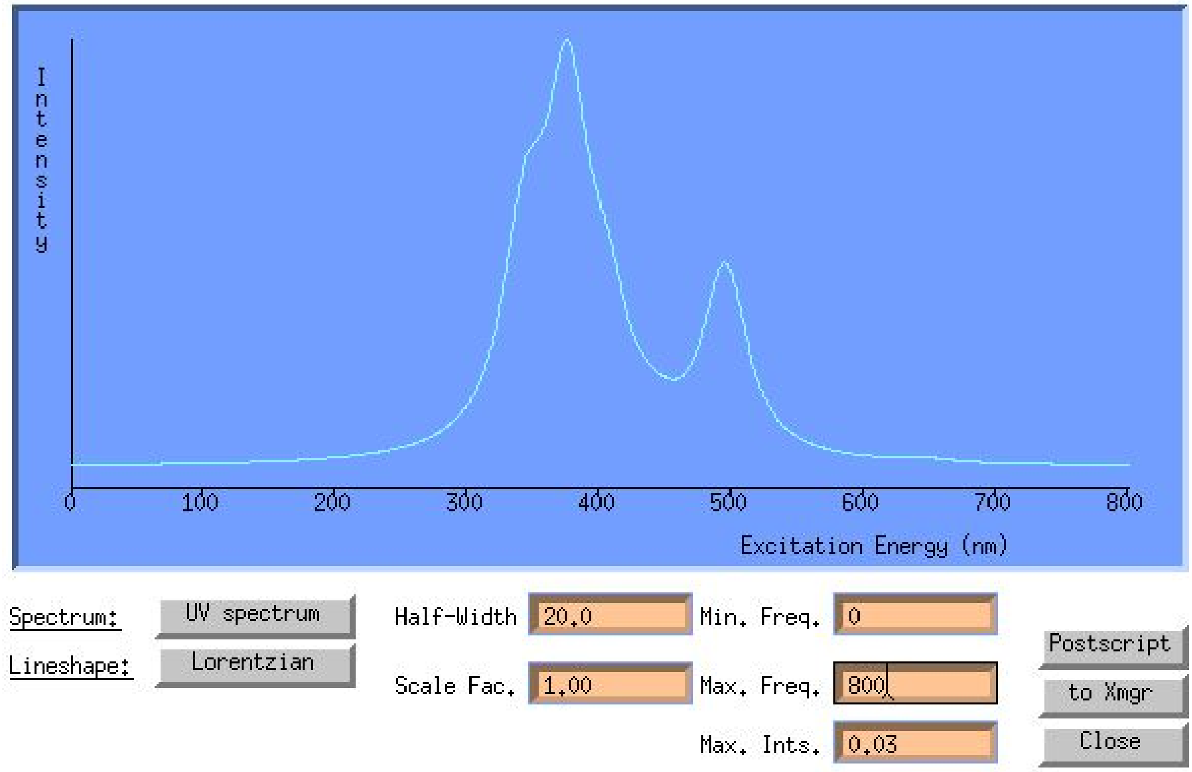

Band at λ = 345 nm, 376 nm, 495 nm

Figure S38: HOMO and LUMO for compound **10**.

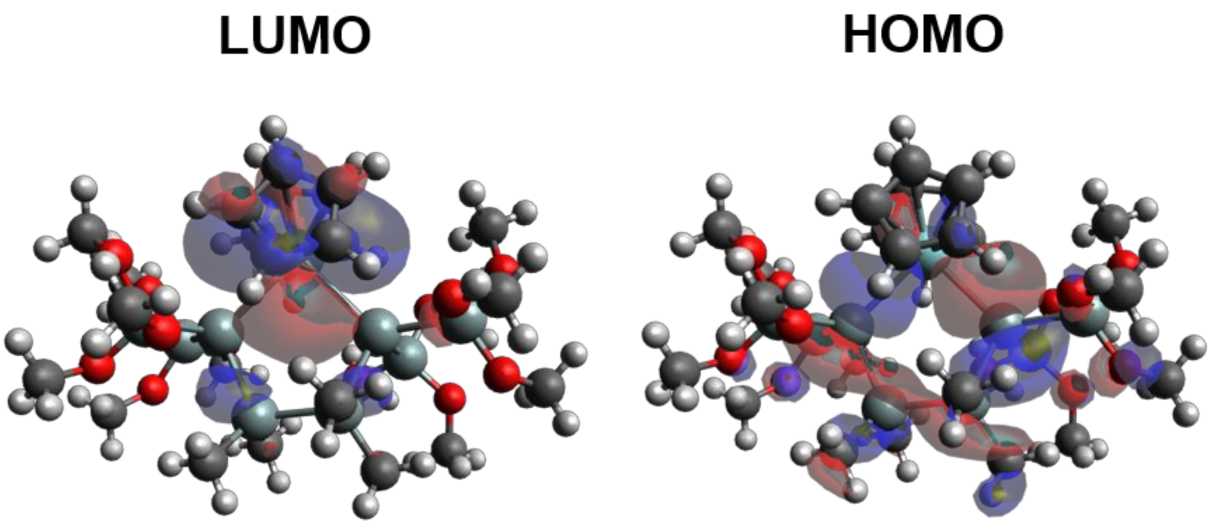

Figure S39: Calculated absorption spectrum for compound **10**.

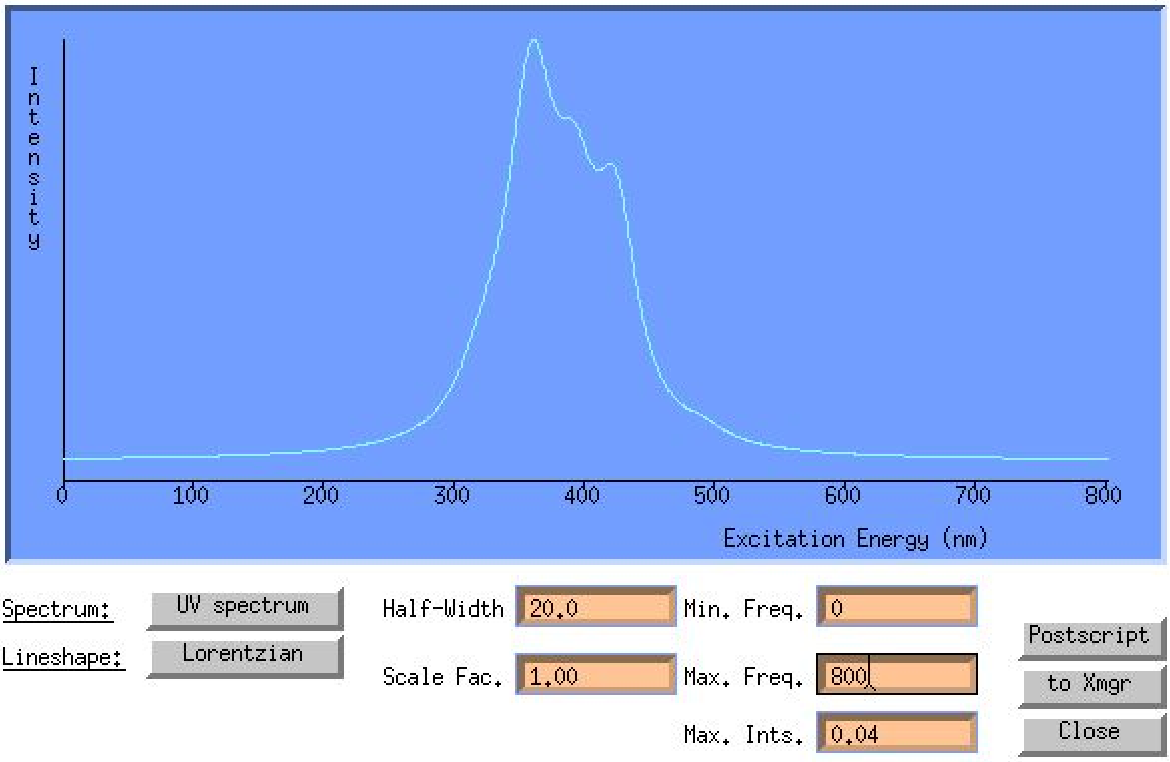

Band at λ = 360 nm, 392 nm, 423 nm

Figure S40: HOMO and LUMO for compound **11**.

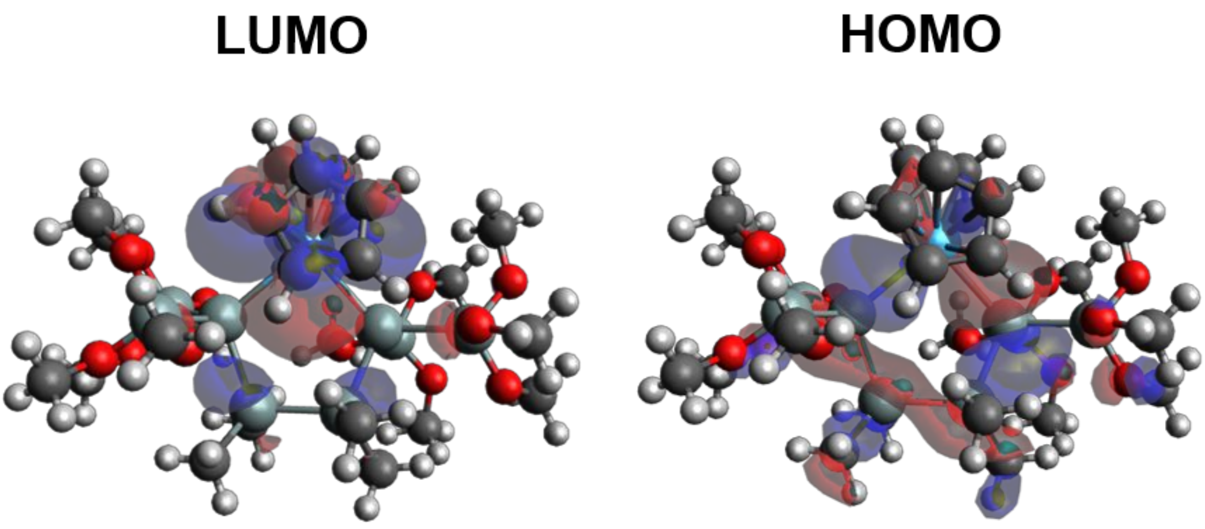

Figure S41: Calculated absorption spectrum for compound **11**.

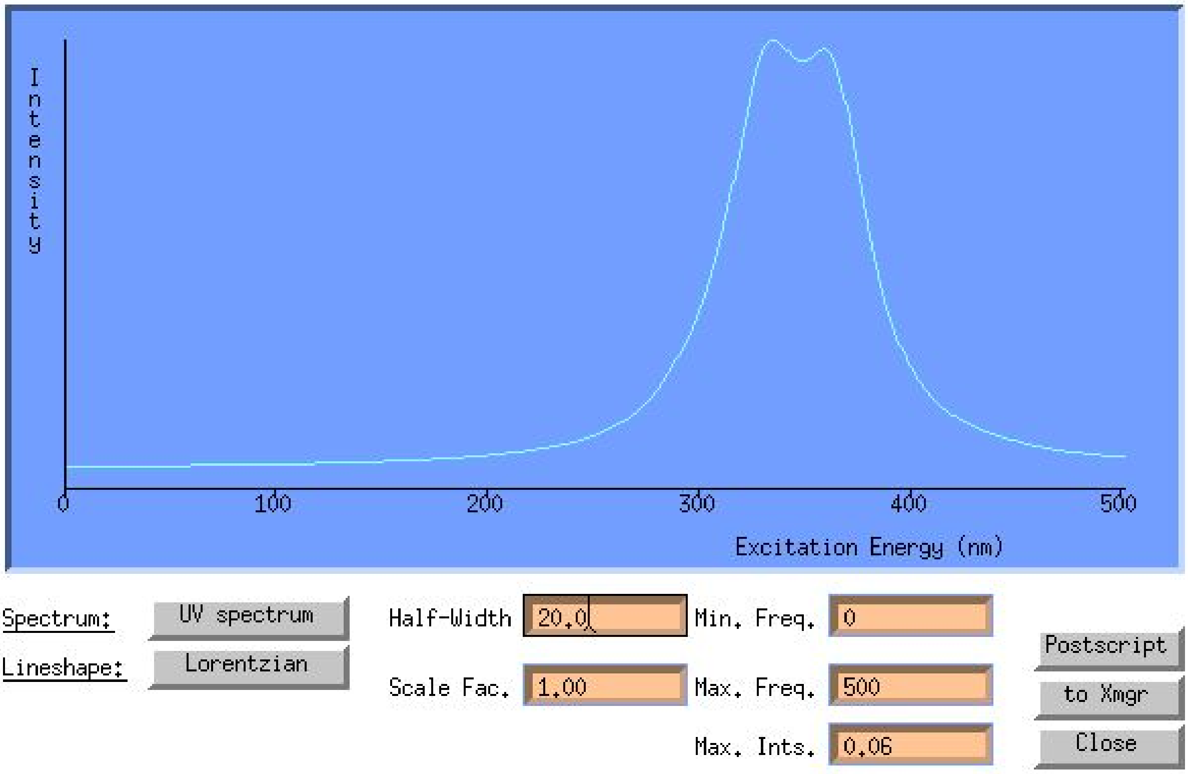

Band at λ = 334 nm, 362 nm
